# Agricultural land degradation consequences as a migration driver in Egypt

**DOI:** 10.1371/journal.pone.0353721

**Published:** 2026-07-17

**Authors:** Muhammad Hussien, Abdelrahman Ali, Yanwen Tan, Mohamed Abdelhameed

**Affiliations:** 1 Department of Geography, Faculty of Arts, Fayoum University, Fayoum, Egypt; 2 College of Public Administration, Institute of Area Studies & College of Economics and Management, South China Agricultural University, Guangzhou, China; 3 Department of Agricultural Economics, Faculty of Agriculture, Fayoum University, Fayoum, Egypt; Flinders University, AUSTRALIA

## Abstract

Environmental migration is a global phenomenon in which increasing soil salinity reduces productivity and income, thereby increasing migration intention (MI) in affected regions. This study investigates how agricultural land degradation (ALD), particularly soil salinity, reduces productivity and influences MIs in Egypt. Employing Protection Motivation Theory (PMT) as a framework, we analyzed primary data from 1,782 respondents using a multinomial logistic model. The empirical findings revealed that socioeconomic characteristics, agricultural engagement, and ALD significantly shape and determine MI. Furthermore, over 35.8% of respondents reported a decline in land productivity in the past decade due to environmental change, with 22.4% actively seeking relocation. Among those willing to migrate, the capital Cairo (40.2%) and international destinations (29.4%) were the most desired. Additionally, gender, age, and income adequacy were identified as crucial predictors in migration dynamics. The findings challenge conventional demographic assumptions by revealing a significant propensity to migrate among older populations and women, groups often considered less mobile. The primary motivations (employment and income) are directly exacerbated by ecological pressures, such as declining land productivity. Therefore, effective policy must address this nexus to reduce threat appraisal by implementing sustainable land and water management and investing in both environmental adaptation (e.g., soil restoration) and human capital (e.g., education, local economic opportunities) to mitigate displacement pressures and foster sustainable rural resilience.

## 1. Introduction

### 1.1. Background

**Fig 1:**
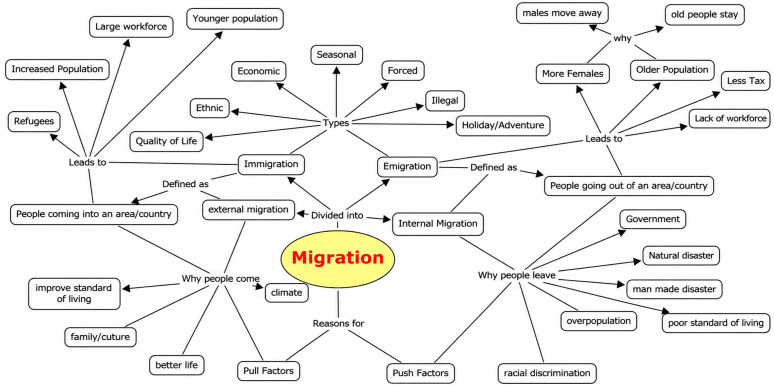
Migration pull and push drivers, causes, and their impacts. People migrate in search of a better life or due to natural disasters, climate change, and environmental degradation. The migration streams have numerous consequences for both the sender and receiver regions (Migration concept map https://cmapspublic3.ihmc.us/rid=1295355828203_878224076_18749/Migration%20Concept%20Map.cmap).

Environmental stresses often exacerbate socioeconomic vulnerability, leading to the erosion of household resources and encouraging migration as a survival strategy for many rural residents [1,2]. Migration has been considered a global challenge in recent years, in which the interaction between environmental changes and socioeconomic factors shapes migration decisions and moves vulnerable communities from one region to another, particularly in developing countries [3,4]. Climate change (CC) is projected to negatively affect from 50 million to one billion people from different regions in 2050, increasing the number of people in movement and migration, and putting pressure on the natural resources in many areas [1,5]. However, environmental migration has been considered a simple indicator of communities’ failure to adopt CC; nevertheless, migrants could support their local families left behind (in the origin areas) through remittances, which will be invested in enhancing infrastructure and improving their families’ lives [6,7]. The pull and push drivers, causes, impacts, and types of migration have been presented in **Fig 1**.

Global migration and population redistribution are increasingly influenced by socioeconomic changes and environmental challenges such as conflict, climate change, water scarcity, pollution, land degradation, and desertification [2,8,9]. People often migrate in search of improved well-being, quality of life, and economic opportunities, with their decisions shaped by social comparisons and external influences [10,11]. Migration outcomes result from complex interactions among socioeconomic, political, and environmental factors [12]. In regions where livelihoods depend heavily on agriculture, land degradation can significantly increase migration pressures [8]. Therefore, efforts to address regional and international migration should include measures to combat land degradation and protect rural livelihoods [13].

In 2019, Egypt was the most prominent African country in terms of migrants, according to the number of people (~4 million citizens) living abroad as migrant labor in the Gulf countries, mainly in Saudi Arabia and the United Arab Emirates [14]. However, the number of people coming to Egypt from surrounding Arab countries is increasing annually due to ongoing civil conflicts in these countries, which is putting pressure on food demand and food security in Egypt, as it is one of the region’s highest food importers [15]. Several empirical studies across various regions have highlighted the link between land degradation and rural-urban migration [1,4,8]. In Africa, countries such as Ethiopia and Niger demonstrate that severe land degradation and recurring droughts compel rural populations to migrate to urban centers in search of better opportunities [16]. In India, studies indicate that land degradation, exacerbated by monsoon variability and water scarcity, contributes to rural out-migration, particularly among smallholder farmers [17]. Additionally, in Mexico, deforestation and soil erosion in rural areas have been identified as significant drivers of migration to urban areas, including to the United States [18].

Furthermore, some case studies provide detailed insights into the mechanisms by which land degradation drives migration. In the Sahel Region of West Africa, land degradation due to overgrazing and unsustainable farming practices has led to significant rural-urban migration, and migrants often move to urban centers or coastal countries seeking employment [19]. In Northern China, desertification in Inner Mongolia has led to large-scale rural displacement. Migrants typically move (temporarily or permanently) to capital cities such as Beijing and Tianjin, seeking better living conditions [20,21]. In Bangladesh, riverbank erosion and soil salinity in coastal areas have forced many rural inhabitants to migrate to urban slums in Dhaka, highlighting the link between environmental degradation and urban poverty [16,22].

The novelty of the current work could be highlighted through the following points: (1) our focus is to analyze how the interaction between socioeconomic characteristics and environmental change leads to migration intention in developing countries based on primary data. We apply a construct framework, combining the principles of the Protection Motivation Theory (PMT) with a multinomial logistic model, to predict the willingness to migrate in the face of potential ALD, which significantly contributes to increased migration streams in developing countries. PMT is a novel and creative theoretical lens for migration; we consider complementing PMT with the livelihoods vulnerability framework and/or Push-Pull models to enhance explanatory power. While existing research has examined the relationship between economic factors and migration [23], few studies have specifically analyzed how ALD influences migrants’ decisions, particularly regarding migration timing [1,3,4,8]. Most prior research has focused on general migration drivers [13,24]. In contrast, our study sheds light on the specific ways climate change determines when migrants will move from one region to another, based on the degree of soil fertility and water availability in their rural areas of origin. (2) At the same time, we used the constructed framework to develop evidence-based recommendations about the specific push regions grounded on the degree of access to climate change mitigation and adaptation tools in rural areas in Egypt, which could contribute to future migration-reduction interventions. The results of the current study could best inform policymakers’ and international donors’ understanding of environmental migration dynamics, thereby enabling the design of a strategy to address environmentally induced migration resulting from soil and land degradation in developing countries.

The structure of this paper is as follows: the next section is the literature review, and Section 3 is the methodology and data source. In Section 4, we presented the results and discussion, concluding with the main findings and policy implications.

### 1.2. Literature review

Rural-to-urban migration is a worldwide issue affecting both developing and developed countries, with significant social, economic, and political consequences [21,25]. Understanding the factors that encourage or prevent migration is important for researchers, practitioners, and policymakers seeking to address its causes and develop effective interventions. The following section reviews and critiques existing studies on how environmental challenges—such as salinity, water scarcity, and changes in crop patterns—reduce agricultural income and contribute to both rural-to-urban and international migration.

A. Land degradation as a rural-urban migration driver

Land degradation, caused by factors such as deforestation, overgrazing, unsustainable farming practices, climate change, and soil salinity, reduces land quality and agricultural productivity, threatening rural livelihoods [4,8]. Globally, about one-quarter of land is considered degraded, leading to the loss of fertile land, lower crop yields, and population displacement (GALDI, 2018) (https://www.isric.org/projects/global-assessment-land-degradation-and-improvement-glada). Soil salinity is particularly harmful in arid and semi-arid regions, including Egypt and North Africa, where it decreases soil fertility and farm income [26]. As agricultural conditions worsen, many farmers abandon their land and migrate to urban areas in search of better opportunities. In Egypt, environmental degradation and salinity have contributed to increased migration of rural youth to major cities such as Cairo and Alexandria [27], placing additional pressure on urban infrastructure and services [28,29].

Water access plays a significant role in global migration [30], while water deficit and scarcity account for about 10% of rent global migration (1 billion migrants) [31,32]. Although floods are a significant driver of migration (recent floods have forced millions of people to leave their homes), the negative consequences are lower than those of drought as a migration push factor [32]. More than 25% of the global population suffers from extreme water stress, especially in the developing world (more than 85% of people affected by rainfall variability live in low- or middle-income countries) [30]. As Egypt is located in the North African dry region, agricultural water shortages in rural areas push populations towards urban centers, where more than 80% of water is used for agriculture [33]. Water scarcity is another critical driver of rural-urban migration, as over-extraction of groundwater, inefficient irrigation practices, and climate change exacerbate water shortages (mmon in the North African region), reducing sustainable water availability for agricultural activities [34]. As a result, crop yields decline, incomes fall, and rural residents are forced to migrate to cities in search of better livelihoods [35]. This appears in the statistics, which show a high-migration region, mostly from the dry and semi-dry regions [31].

Changes in crop patterns are often driven by environmental degradation and/or market forces, leading to decreased agricultural productivity and income, and farmers may be forced to switch from high-value to low-value crops due to changing climatic conditions or soil degradation [2,36]. This shift reduces profitability and makes farming unsustainable (in many cases), prompting rural residents to migrate to urban areas [34]. Some studies analyzed the impacts of the Green Revolution on migration, noting how changes in crop patterns due to environmental stressors affect rural incomes and migration patterns [37]. The same case is evident in the study area in Fayoum, which was the first city in Egypt to cultivate rice since 640 A.D. [38]. Rice was the main crop for farmers in Fayoum. Still, due to water shortages in this region since the 2000s, the Egyptian government issued a new regulation in 2008 to prohibit rice cultivation in Fayoum, aiming to ensure water sufficiency for other field crops in other regions. While *Morton* (2007) explores the impact of climate change on smallholder agriculture, highlighting how shifts in crop patterns driven by climate variability lead to decreased income and increased migration due to their vulnerability to adapt or mitigate these negative impacts [39].

B. Socioeconomic drivers and impacts of migration

Socioeconomic factors also play a crucial role in understanding migration decisions; in addition to education, better healthcare, and improvements in lifestyle, urban areas often offer greater access to these services and opportunities, attracting people from rural areas who want to improve their quality of life [40,41]. In rural-urban migration, economic opportunity is considered the main driver, with migrants seeking better employment opportunities in urban centers, improved living conditions, higher wages, and more diverse jobs that are typically unavailable in rural areas [40]. The lack of sustainable livelihoods and economic opportunities in rural areas, such as insufficient arable agricultural land and limited viable off-farm activities that generate additional income beyond agriculture, can be an incentive for individuals to seek new, higher-income employment opportunities in urban areas [42]. Furthermore, the perceived economic prospects and greater availability of public services in urban areas compared to rural ones may provide a strong incentive for rural residents to move to urban areas [21,43]. According to the International Migration Report, migration can contribute positively to economic growth by addressing labor shortages, particularly in sectors with high demand but limited local labor supply (e.g., agriculture & construction) [44]. Furthermore, migrants often send remittances back to their home countries, which can significantly contribute to poverty reduction and improve the living standards of families left behind (World Bank, 2021) (https://www.worldbank.org/en/topic/migrationremittancesdiasporaissues/brief/migration-remittances-data).

Environmental changes, such as land degradation, climate change, and natural disasters, often prompt rural populations to migrate to urban areas [1,45]. Environmental stresses often exacerbate socioeconomic vulnerability, eroding household resources and making traditional agricultural livelihoods unsustainable, thereby encouraging many rural residents to migrate as a survival strategy [46]. As highlighted in the literature, the interaction between environmental changes and their socio-economic consequences is a complex process to understand the dynamics of migration [1], in which factors such as environmental changes, available adaptation strategies, and the accessibility of migration options play a crucial role in the decision-making process of the rural population [47].

On the other hand, increased migration can strain public services in urban areas, such as healthcare and education, especially in areas with high migrant concentrations, leading to budgetary challenges for governments (Migration Policy Institute, 2020). Moreover, cultural differences and perceived competition for resources can sometimes lead to social tensions and conflicts within communities and reduce average wages due to migrants’ experience nd the high labor supply [32]. Furthermore, pressure on the infrastructure in the receiver cities will reduce their ability to withstand sudden shocks and increase losses during natural disasters such as floods and storms. At the same time, food producers will decrease, increasing the need for food import vouchers, especially in developing countries like Egypt. Also, it will increase migration from rural to urban areas, potentially reducing economic growth in rural areas over time.

C. Costs of rural-urban migration

Despite the potential benefits of migration, economic barriers such as moving costs, poor creditworthiness, and the risk of unemployment in urban areas can prevent migrants from moving [24,47]. When making a migration decision, potential migrants typically compare the costs of migration with its benefits. Migrants may face different costs, such as travel costs to the destination, subsistence costs while seeking work and housing, and any opportunity costs associated with activities in the area of origin [13,48,49]. In addition, migrants face other costs due to differences in religion, culture, language, and ethnicity, which together shape the migration decision [41]. For this reason, potential migrants prefer destinations with a similar linguistic or ethnic background [49]. On the other hand, migrants living in communities with diverse ethnic backgrounds may be able to integrate better than those living in neighborhoods with few ethnic groups [50].

Rural-urban migration often reduces the agricultural workforce in rural areas, thereby impacting farm productivity and food security [51]. In urban areas, migrants may compete with locals for jobs, potentially driving down wages in specific sectors and contributing to higher unemployment rates among low-skilled native workers [51]. Furthermore, rapid urbanization due to migration strains urban infrastructure such as housing, transportation, water, and sanitation services, leading to inadequate living conditions in some cities. Moreover, increased urbanization and migration contribute to environmental degradation, including air and water pollution, deforestation, and biodiversity loss [45,52]. Migration also affects one’s physical and mental well-being. For instance, a recent study reveals that younger migrants from rural to urban areas of China report worsening health, which they attribute to shifts in social trust and emotional states [53]. In addition, older parents of migrants who settled in rural areas are more likely to experience health problems [54]. Migration can also result in social disintegration within rural communities as families are separated, leading to breakdowns in traditional support systems [55].

### 1.3. Theoretical framework

The conceptualization of migration studies has been historically grounded in *Ravenstein’s* ‘Laws of Migration’ (1885) [56], which first identified systematic patterns of migration flows, and later expanded by *Everett Lee’s* Push-Pull Theory (1966) [57], which emphasized the interaction of push factors in origin areas and pull factors in destination areas. These classical theories provide the intellectual foundation for more recent approaches, including the Protection Motivation Theory (PMT), which explains environmentally induced migration, as described below.

The migration theories investigate the social change processes interlinked with human mobility, so many theories and approaches have been used to understand the root causes, motivations, and benefits of migration. For example, De Haas has investigated the aspirations and capabilities of migration within given sets of perceived geographical opportunity structures and differentiates between human mobility’s instrumental (means-to-an-end) and intrinsic (directly wellbeing-affecting) dimensions [58,59]. This yields a vision in which moving and staying are seen as complementary manifestations of migratory agency and in which human mobility is defined as people’s capability to choose where to live, including the option to stay, rather than as the act of moving or migrating itself [59]. Furthermore, labor policies in destination countries play a significant role in facilitating the immigration of highly skilled labor to areas with risky environmental conditions, where land degradation and the negative impacts of climate change push migrants to move to safer areas. At the same time, rural citizens may move to nearby cities or migrate to major capital cities, and some may even immigrate to seek opportunities in another country. Climate change and natural disasters often prompt rural populations to migrate [1,45].

This study adopted the Protection Motivation Theory (PMT) to interpret the impact of environmental changes and stresses, such as land degradation, as drivers of migration. The PMT was developed by Rogers in 1975 and modified in 1983 as a framework for analyzing how individuals react to perceived threats [60,61]. PMT initially focused on health-related behaviours, but it can also be applied to migration decisions, especially when individuals encounter environmental, economic, or social challenges.

**Fig 2:**
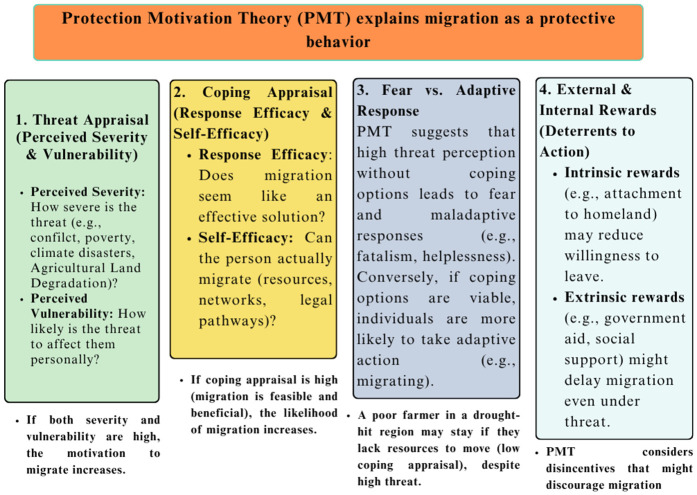
Protection Motivation Theory (PMT) explains migration as a protective behavior.

PMT is a novel and creative theoretical lens for migration; we consider complementing PMT with the livelihoods vulnerability framework and/or Push-Pull models to enhance explanatory power. The following outlines how the primary elements of PMT—threat appraisal and coping appraisal—can clarify migration behavior, **as shown in Fig 2**:

1. **Threat appraisal (perceived severity and vulnerability)**

Migration often happens once individuals perceive a substantial threat to their safety, well-being, or livelihood. For example, farmers facing ALD may perceive yield reductions as a severe threat and believe they will face socioeconomic vulnerability if they remain in their origin regions, which encourages many rural residents to migrate [38]. Furthermore, the interaction between environmental changes and their socioeconomic consequences is a complex process [1], in which factors such as environmental changes, available adaptation strategies, and the accessibility of migration options play crucial roles in the decision-making of the rural population [46], as shown in **Fig 2**.

2. **Coping appraisal (response efficacy and self-efficacy)**

Although the threat is perceived as severe, the decision to migrate depends on the individual’s beliefs about how to respond effectively. For example, farmers facing ALD may feel able to migrate if relocation to another place offers better income and living conditions, and they have legal pathways and resources (savings and relatives abroad), whereas poor growers may not.

3. **Fear vs. adaptive response**

Despite the high threat to the poor farmers in the region with ALD, they may stay if they have insufficient resources to relocate (low coping appraisal).

4. **External & Internal Rewards (Deterrents to Action)**

PMT also considers disincentives that may restrict, delay, or reduce the willingness to migrate under threat, including government aid, social support, and attachment to one’s homeland.

**Fig 3:**
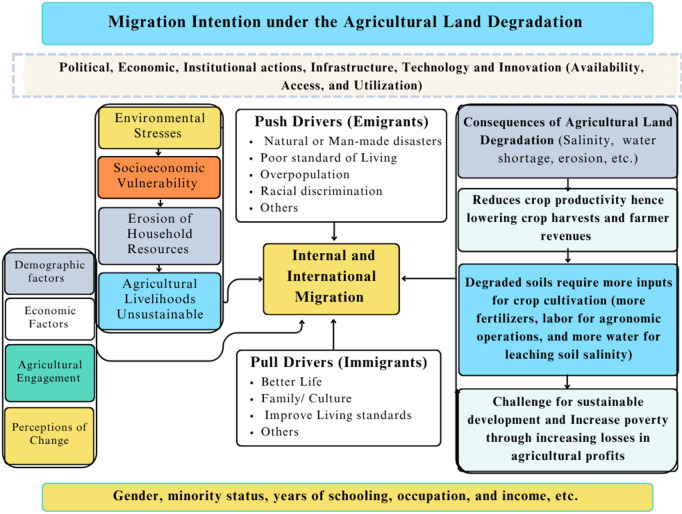
Migration drivers: the push-pull factors of migration under agricultural land degradation, besides the potential consequences of environmental change.

We summarize the potential consequences of environmental change, particularly the ALD, to highlight the interaction between socioeconomic and environmental stress as a driver of migration. As illustrated in **Fig 3**, the consequences of agricultural land degradation (ALD) on migration intention can be interpreted through the Protection Motivation Theory (PMT). Specifically, ALD increases threat appraisal by raising farmers’ perceived vulnerability (e.g., declining productivity, income loss) and the severity of threats (e.g., livelihood insecurity). At the same time, migration as a coping response depends on coping appraisal, where individuals evaluate their ability to relocate successfully given available resources, skills, and networks. The interaction of these processes, heightened threat appraisal alongside varying levels of coping appraisal, shapes the willingness to migrate depicted in the figure. Thus, **Fig 3** integrates PMT dimensions with empirical evidence of ALD-induced migration intentions.

## 2. Materials and methods

### 2.1. Study area and sampling strategy

**Fig 4:**
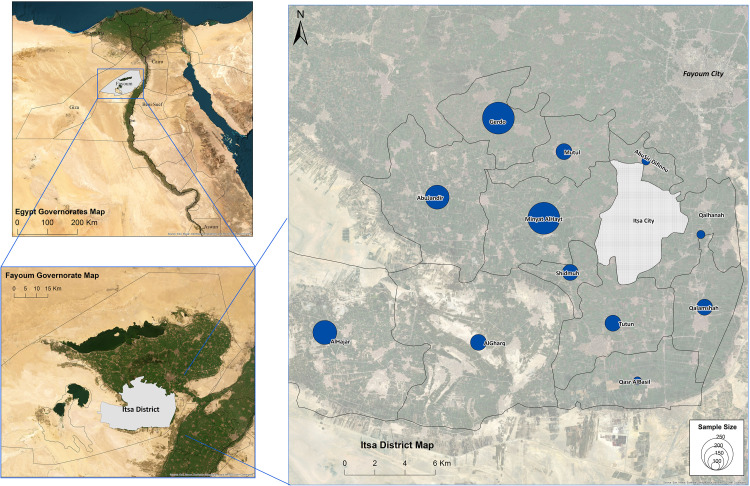
Study area map and distribution of the selected respondents. Sources: Esri, Fayoum Governorate, and Itsa district maps. (December 25, 2024). https://www.arcgis.com/home/item.html?id=09c9d0af95d24bc7a1b6d8859b59e428.

The current study adopted a multi-stage approach (cluster sampling followed by systematic random household selection within each cluster), ensuring that the sample adequately represents the demographic and agricultural diversity of the selected community, making the findings generalizable to the broader rural population. We first chose Fayoum Governorate, Egypt (Fig 4), which is often described as a microcosm of Egypt due to its geographic, agricultural, and demographic characteristics. Fayoum, as an oasis in the desert, suffers from water shortages because it lies on the downstream branch of the Nile River (Bahr Youssef canal), the only water source (for agriculture and drinking), which plays a role similar to that of the Nile River for Egypt. Its waters discharge into *Lake Qarun* and *Wadi El-Rayan* areas, which resemble Egypt’s Mediterranean outlets. Fayoum is one of the leading exporters of both internal and international migrants in Egypt. Many of its villages are well known for exporting international migrants, such as *Tatoun city (*to Italy*) and Shidmu city (*to France*).* The governorate is predominantly rural, with about 26.4% of its population living below the poverty line in 2019. The average percentage of the rural population in Egypt was 57.6% in 2017; this percentage rose to 76.7% in Fayoum Governorate, and within Fayoum Governorate itself, the percentage of rural people rose to more than 90.4% in the *Itsa* district (CAPMAS, 2017).

Fayoum is considered a representative of Egypt in the water shortage issue (downstream of the Nile River Basin), where water shortages in upstream countries will negatively affect Egypt, a country located in a dry region and suffering from groundwater limitations, exacerbating the problem, especially for agricultural water. This water shortage resulted in many issues, including high soil salinity, decreased agricultural productivity and income, and changes in crop patterns over the last decades (to mitigate the negative impacts of this water shortage). Previously (before 2008), rice was the main crop in Fayoum (*Itsa* districts*)*. However, after the prohibition on rice cultivation, soil salinity increased in this region, and many farmers left the land or saw their agricultural income decline as their primary source of income.

For the geographical distribution of the selected sample, we chose *Itsa* district as the study area, as the last district in the *Yousef canal* branch, and it is the largest district in terms of both area and population (708,780 people, about 20% of the governorate’s population in 2017) (CAPMAS, 2017) (CAPMAS (Central Agency for Public Mobilization and Statistics, Egypt) www.capmas.gov.eg). In total, 1,782 households were investigated. One adult respondent was randomly selected from each sampled household. The overall sample size was also cross-validated using Cochran’s formula (1977) at a 95% confidence level and a 5% margin of error [62], and was adjusted for potential non-response. To ensure representativeness, a cluster sampling strategy was adopted. The twelve local villages of *Itsa* district were divided into four geographically contiguous clusters, each forming a sub-region within the district, as shown in **S1 Appendix**. Sample sizes in each cluster were calculated using Thompson’s formula and cross-checked with Krejcie and Morgan’s (1970) sampling tables to meet minimum requirements [63].

A structured questionnaire was developed and pretested to ensure its accuracy in achieving the study objectives. We interviewed the respondents face-to-face in 2023, and they self-reported their responses to the proposed questions.

### 2.2. Data analysis and empirical approach

Many econometric models have been employed to understand the effects of socioeconomic demographics on the likelihood of changing residence, moving from rural to urban areas, or immigrating abroad. In this study, the Multinomial Logistic Regression (MLR) model was employed to analyze the impact of socioeconomic demographic characteristics on the ability and willingness to migrate.

Logistic regression (LR) is used when the dependent variable is nominal (or categorical). For Binary logistic regression (two dependent variables) and multinomial logistic regression, there are more than two [64]. In LR, a logistic transformation of the odds (referred to as logit) serves as the dependent variable [64,65]:


$$\begin{array}{c} \text{log}\left(odds)=\text{logit }(\text{P}\right)=\text{ln}\left(\frac{P}{\text{1}-P}\right)=a+{\beta_{\text{1}}x}_{\text{1}}+{\beta_{\text{2}}x}_{\text{2}}+{\beta_{\text{3}}x}_{\text{3}}\ldots \;{\beta_{\text{n}}x}_{\text{n}} \end{array}$$ (1)

Or


$$\begin{array}{c} \text{P}=\frac{exp(a+{\beta_{\text{1}}x}_{\text{1}}+{\beta_{\text{2}}x}_{\text{2}}+{\beta_{\text{3}}x}_{\text{3}}\ldots \ldots )\;}{\text{1}+exp(a+{\beta_{\text{1}}x}_{\text{1}}+{\beta_{\text{2}}x}_{\text{2}}+{\beta_{\text{3}}x}_{\text{3}}\ldots \ldots )} \end{array}$$ (2)

Where:

p = the probability that a case is in a particular category,

exp = the exponential (approx. 2.72),

a = the constant of the equation and,

b = the coefficient of the predictor or independent variables.

Logits or Log Odds

Odds value ranges from zero to infinity and tells us “how much more likely it is that an observation is a member of the target group rather than a member of the other group” [50].


$$\begin{array}{c} \text{odds}=\text{p}/(\text{1}-\text{p}) \end{array}$$

The odds ratio (OR) estimates the change in the odds of membership in the target group for a one-unit increase in the predictor [64]. It is calculated using the predictor’s regression coefficient as the exponent (exp).

In our case, the Y (dependent variable) is the desire to change the place of residence and leave the village (Migrate), where “0” is “No, I don’t want”, “1” is “Maybe I want”, and “2” is “Yes, I want”. The explanatory variables include demographic, economic, and agricultural factors, as presented in **S2 Appendix**. For the perceptions of change based on available data that include (age, family size, agriculture share of income, gender, marital status, educational status, monthly income, income adequacy, working in the agricultural sector, ownership of agricultural land, changes in land ownership during the last 10 years, changes in the farming land during the previous 10 years). The cultivation of rice and beet crops, changes in land productivity, the farmer’s living conditions now compared to 10 years ago, and current village conditions compared to 10 years ago have been considered.

The reference category for gender is “Males”, marital status is “Married”, educational Status is “high-school”, and monthly income is “from 2000 to 4000 LE. (1 $USA = 30.743 LE (Egyptian pound as an average 2023) based on the Central Bank of Egypt data. https://www.cbe.org.eg/en)”, income adequacy is “Somewhat enough”, working in the agricultural sector is “Yes”, ownership of agricultural land is “Yes”, Changes in land ownership during the last 10 years is “Area has not changed”, Changes in the farming land (cultivated area) during the last 10 years is “Area has not changed”, Rice crop cultivation is “No cultivation”, Beet crop cultivation is “No cultivation”, Changes in land productivity is “Now better”, Living conditions of the farmer now compared to 10 years ago “Now better”, and Village conditions now compared to 10 years ago is “Now better”.

Finally, we used the weighted percentage to rank the motivations and barriers of migration under the ALD as follows: Weighted percentage % = $\frac{\text{Total marks}}{\text{N}*\text{3}}$ *100, where N represents the sample size. We used the same model to assess the impact of respondents’ socioeconomic characteristics on motivation and barriers to migration intention using a composite variable.

## 3. Results and discussion

The following analysis is guided by the Protection Motivation Theory (PMT), which frames migration intention as an outcome of both threat appraisal and coping appraisal. In this study, threat appraisal is reflected in how farmers perceive the severity and vulnerability associated with agricultural land degradation, while coping appraisal is captured by their resources, education, and access to alternative livelihood strategies. Accordingly, each set of results is interpreted in relation to these theoretical dimensions to ensure consistency between the framework and empirical findings. Environmental changes, such as land degradation, climate change, and natural disasters, often prompt rural populations to migrate to urban areas [1,45]. In the following section, we will discuss the main results, including the interaction between socioeconomic factors and environmental stress as drivers for migration.

### 3.1. Descriptive statistics

The respondents’ socioeconomic characteristics are presented in S2 and S3 Appendix. More than two-thirds of the respondents (83%) were males and married (68.9%), and most were educated (80%). However, more than 40% of them don’t own agricultural land, and around one-quarter (26%) depend on agriculture as a primary source of income. The descriptive statistics reveal significant trends in land ownership and farming areas. Nearly half of the respondents (46.3%) reported no change in land ownership over the past decade, while 53.3% indicated stability in the area they currently cultivate. This reflects limited land-market activity and the role of inheritance in maintaining land-ownership structures. This result is consistent with Deininger and Jin, who noted that land tenure systems in developing countries often restrict land redistribution, leading to long-term stability in ownership [66].

Regarding crop structure, rice remains a dominant crop, with 60.4% of respondents cultivating it only within the past 10 years, highlighting its importance in local agricultural systems in the investigated area. This finding highlights the difficulty of changing the cropping system, which aligns with Elmpghazy and Elshenawy, who reported that rice is a staple in rural Egypt due to its profitability and long-standing farmers’ experience, and that it is a main crop in most northern Egyptian governorates [38]. For sugar beet cultivation, the distribution is more balanced, with 36.9% cultivating beet both currently and a decade ago, suggesting the role of crop rotation and adaptation to market demands [3].

A concerning trend is the decline in land productivity, as reported by 35.8% of respondents. This decrease may be attributed to factors such as soil degradation, water shortages, and climate change. Mahmoud (2019) found similar patterns in Egypt, where environmental stressors have contributed to declining agricultural productivity, lower farm incomes, and increased migration rates [33]. Economic challenges further exacerbate this, as the study shows that 43.4% of respondents earn between 2000 and 4000 EGP (less than $80) per month. This range limits financial stability and motivates people to migrate in search of a better life for their families. Moreover, only 26.6% of respondents considered their income sufficient, reinforcing findings from Radwan et al. (2022), who highlighted persistent rural poverty in Egypt due to limited off-farm employment opportunities [67].

The study also captures perceptions of agricultural production and living conditions. A significant proportion of respondents (44.2%) believe that agricultural production has degraded over the past decade. Zohry (2005) attributes this decline to increased production costs, limited access to quality inputs, and environmental challenges. Similarly, more than half (53.4%) of respondents reported that living conditions have worsened over the last 10 years due to higher living costs and a decrease in income sufficiency [29]. This trend is supported by Abdelwahed et al. (2020), who found that rural communities in Egypt have struggled due to inflation and economic reforms that have not equally benefited all social classes [28]. This highlights the significant role of social protection and women’s empowerment in reducing the streams of migration from the vulnerable areas.

Migration trends show that 45.2% of respondents do not wish to leave their community, while 22.4% actively seek relocation, a topic discussed in the motivation and barriers section later. Among those willing to migrate, Greater Cairo (40.2%) and destinations outside Egypt (29.4%) were the most desired. These patterns reflect broader economic migration trends, where rural populations move to urban centers or abroad in search of better opportunities for their families [29]. The primary motivations for migration were access to suitable job opportunities (70.1%) and higher wages (66.8%), consistent with Abdelwahed et al. (2020), who emphasized that economic factors are the strongest drivers of rural-to-urban migration in Egypt [28]. This highlights the importance of empowering farmers to use advanced technology, thereby enhancing their incomes and reducing migration from vulnerable rural areas to major cities or abroad.

Despite the desire to migrate, several barriers exist, with the most cited obstacles being the difficulty of moving (54.1%) and the high cost of relocation (52.7%). Our findings align with those of Abdelwahed et al. (2020), who found that limited financial resources and the lack of affordable housing often deter rural residents from moving. Similarly, inadequate services in rural areas also influence migration decisions, with 48.5% of respondents dissatisfied with both educational and healthcare services [28]. These findings align with Zohry (2005), who reported that rural Egypt faces severe challenges in access to quality education and healthcare due to underinvestment in infrastructure [29]. This underscores the essential need for equitable economic development across regions to reduce migration pressure and prioritizes vulnerable areas for public investment in education, health, and infrastructure.

### 3.2. Determinants of migration intention

The results of the migration determinants that play a significant role in motivating or inhibiting people from migrating from one region to another are presented in **Table 1 and Table 2**. The interrelationship between these variables and willingness to relocate is represented as (yes, I will relocate) or (maybe). This includes the demographic, economic, and farming systems of the selected respondents, presented as follows;

**Table 1:** Overall effect of the main variables on the migration intention.

Likelihood Ratio Tests			
Effect	Model Fitting Criteria	Likelihood Ratio Tests	
	−2 Log Likelihood of Reduced Model	Chi-Square	df
Intercept	3272.342^a^		
Age	3389.866	117.523^***^	2
Family Size	3278.914	6.572^**^	2
Agricultural share of total income	3276.911	4.569	2
Gender	3290.362	18.020^***^	2
Marital Status	3277.869	5.527	6
Educational Status	3294.111	21.769^**^	10
Working in the agricultural sector	3277.028	4.686^*^	2
Agricultural land ownership	3273.606	1.264	2
Changes in Agricultural land ownership in the last 10 years	3274.118	1.776	6
Changes in total cultivated area in the past 10 years	3276.300	3.958	6
Monthly Income	3315.200	42.858^***^	6
Income adequacy	3289.551	17.209^**^	4
Changes in land productivity	3281.266	8.924^*^	4
Living conditions of the farmer now (compared to 10 years ago)	3280.092	7.750	4
Changes in village conditions compared to 10 years ago	3275.188	2.846	4
**Model Fitting Information**			
**Model**	**Model Fitting Criteria**	**Likelihood Ratio Tests**	
	**−2 Log Likelihood**	**Chi-Square**	**df**
Intercept only	3772.331		
Final	3272.342	499.989^**^	80
**Pseudo R-Square**			
Cox and Snell	0.245		
Nagelkerke	0.278		
McFadden	0.132		

The chi-square statistic is the difference in −2 log-likelihoods between the final model and a reduced model. The reduced model is formed by omitting an effect from the final model. The null hypothesis is that all parameters of that effect are 0.

a. This reduced model is equivalent to the final model because omitting the effect does not increase the degrees of freedom.

*** Values are significant at P = 0.01, ** Values are significant at P = 0.05, * Values are significant at P = 0.1

**Table 2:** The willingness of the respondents to migrate from their current place (results of the multinomial regression models).

	(1) Maybe		(2) Yes	
Do you want to change your place of residence and leave the village? (Maybe or yes)	Std. Error	Exp(B)	Std. Error	Exp(B)
Intercept	0.366		0.420	
Changes in land productivity = 2] As it was 10 years ago			0.226	0.559**
Age	0.006	0.944**	0.007	0.951***
Family Size	0.032	1.078*	0.035	1.068**
Agriculture’s share of income	0.218	0..662	0.006	1.018
[Gender=1] Females	0.185	0.521***	0.194	0.495***
[Educational Status=1] Illiterate	0.191	1.038	0.219	0.753
[Educational Status=2] Reads and writes	0.186	1.161	0.219	0.918
[Educational Status=3] Basic education	0.280	1.072	0.331	0.900
[Educational Status=4] University and post-university	0.200	1.760**	0.213	2.089**
[Educational Status=5] Institute (above intermediate)	0.261	1.681*	0.314	1.240
[Do you work in the agricultural sector?=0] No	0.166	0.725*		
[Income (monthly)=1] (less than 2000)	0.173	0.573***	0.173	1.735**
[Income (monthly)=2] (4000- 6000)	0.156	0.826	0.193	0.783
[Income (monthly)=3] (more than 6000)	0.206	0.828	0.231	1.013
[Income adequacy=1] Not enough	0.144	1.014		
[Income adequacy=2] Enough	0.155	0.597***		
Living conditions of the farmer now compared to 10 years ago= (Now worse)	0.171	1.439**		
Living conditions of the farmer now compared to 10 years ago = (As it was 10 years ago)	0.200	1.083		

*** Values are significant at P = 0.01, ** Values are significant at P = 0.05, * Values are significant at P = 0.1

#### 3.2.1. Model fitting and Pseudo R-square.

The multinomial logistic regression model was evaluated using several key metrics, beginning with model-fitting information. The final model had a significantly lower −2 Log Likelihood value (3272.3) than the intercept-only model (3772.3), indicating a better fit for the full model. The Chi-Square statistic for the model was 499.98 with 80 degrees of freedom, which is statistically significant (p < 0.001). This suggests that the model as a whole is a good fit for the data.

Pseudo R-square values, including Cox and Snell (0.245), Nagelkerke (0.278), and McFadden (0.132), further indicate the model’s explanatory power. While these values do not have the same interpretation as R-squared in linear regression, they do provide a measure of the proportion of variance explained by the model. In this case, the Nagelkerke R-square value of 0.278 suggests that the model explains about 27.8% of the variance in the dependent variable, which is moderately strong for social science data.

#### 3.2.2. Demographic factors.

**Age**: The results show that age is a significant predictor (p < 0.001) across all categories, with an odds ratio (Exp(B)) less than 1 (0.944) for “maybe” and 0.951 for “yes”. This suggests that with increasing age, the respondent’s willingness to migrate and leave the village will decrease. This is consistent with previous studies’ findings that younger individuals were more inclined to migrate than older individuals under the same risk factors. A recent study found that young Egyptians (18–29 years) are more motivated to migrate, depending on age, marital status, and employment status [28]. Although the employment status was insignificant as a migration driver after the Arab Spring of 2011, compared with before it (2008), the remaining factors still significantly drive internal and international migration [28]. For instance, especially in rural areas, older adults are generally more inclined to stay in their current residences due to established social networks and greater attachment to their communities [28].

Furthermore, the youth could be more aware of the negative impacts of climate change, thereby increasing their willingness to move from the most affected regions (rural) and sectors (agriculture) to other service sectors inities [41]. Based on the PMT assumptions, our results confirm that the youth are more motivated to relocate due to their higher perceived severity and vulnerability, and lower restrictions on delaying relocation, compared to older people with families. However, the willingness to migrate will be lower under the government aid, social support, and attachment to one’s homeland, which should be considered for future intervention.

**Family Size** also emerged as a significant predictor for the “maybe” category (p = 0.016, Exp(B) = 1.078), suggesting that larger families are more likely to consider moving as a whole or send some family members to capital cities or to abroad as well to secure more income for the family members, which consistency with Abdelwahed et al. (2020) [28]. This could be because larger families might seek better living conditions or more opportunities, which aligns with previous research highlighting the impact of family size on migration decisions, such as the study by Mulder and Cooke (2009), who observed that households with more members may seek relocation to improve living conditions or access better services [68]. Due to the nature of social networks in Fayoum, a tribal society, most families prefer to have more children, especially males who will later immigrate to EU countries (it is considered a social status in this society). Larger families might also relocate to urban areas to access better educational opportunities for their children, a common trend observed in rural-to-urban migration studies. At the same time, changes in the crop pattern in Fayoum could be another reason. The previous crop rotation, which included rice and cotton, resulted in a labour surplus due to the need for intensive human labour in farm operations and during harvesting. The fragmentation of agricultural land (under the limitation of reclaiming new lands) has another impact on reducing per capita land share and decreasing the rural income due to the land and water limitations in the study area, where the literature revealed that the fragmentation of agricultural land decreases the profitability of farms in the rural areas and leads to migration [69].

**Gender** significantly affects the decision to move, with males being less likely to want to change residence (p < 0.001; Exp(B) = 0.521 for “maybe” and 0.495 for “yes”). This gender difference is consistent with the literature, which suggests that men often have stronger ties to land and agriculture, making them less inclined to migrate. This supports findings from studies such as those by Mulder and Cooke (2009) [68], who noted that women, particularly in patriarchal societies, may seek to relocate for better employment opportunities and greater autonomy. This pattern reflects broader gender dynamics in which women may perceive urban areas as offering greater freedom and opportunities. However, given the nature of Egyptian social norms, it would be expected that women have a lower willingness to migrate; however, due to globalization and access to ICT, the new generation of women is more willing to migrate than [55]. This could go back to land ownership in rural areas in Egypt, which is mostly registered in the names of male householders rather than women, increasing women’s desire for greater empowerment that is more readily available in urban regions.

Furthermore, the gender differences observed are significant, highlighting the ongoing gender disparities in rural areas where men might have more opportunities locally. At the same time, women perceive urban areas as avenues for greater autonomy and employment. This is consistent with the literature, which shows that rural women often migrate to escape restrictive social norms and pursue independent livelihoods [70]. On the other hand, women seeking greater empowerment prefer to leave the village to pursue better educational and employment opportunities, as well as a higher standard of living, in an urban area [71].

**Educational Status**: Education level was significant for both categories (yes, maybe), particularly at higher levels (p < 0.001 in some categories). Individuals with higher education were more likely to consider moving (Exp(B) = 1.760 for “maybe” and 2.089 for “yes”). This finding supports previous studies demonstrating that education increases awareness of opportunities outside rural areas, thereby increasing the likelihood of migration [71,72]. For example, education is noted to significantly predict immigration intentions, particularly among younger adults seeking professional development [72]. The recent literature indicates that education has a significant impact on motivating young people to migrate; at the same time, those who have already migrated invest more in supporting their families and providing their children with a good education [32,7]. A recent study found that young Egyptian laborers have more opportunities in Arab countries when they have higher education than uneducated people [28]. In the same vein, highly skilled and educated individuals are more likely to immigrate, particularly if they have already relocated from their birth village (internally) to pursue education in the city or abroad.

Our findings indicate that demographic and social factors, particularly age, family size, gender, and education, play a crucial role in determining the likelihood of relocating. Based on the PMT assumptions, our results suggest that older individuals are more inclined to remain in the village, possibly because of stronger ties to the community or a reduced propensity to change. In contrast, higher educational attainment and greater income levels increase the likelihood of wanting to leave, possibly reflecting aspirations for better opportunities elsewhere [45,73]. These results align with the broader literature on migration and residence decisions, particularly in rural contexts [73]. The significant role of the combination (age, family size, gender, and education) reflects well-documented trends in the migration literature. For instance, younger individuals and those with higher levels of education are typically more mobile, seeking better opportunities in urban areas [28,71]. Our results confirm the PMT assumptions, indicating that higher perceived severity and vulnerability increase motivation to relocate, while lower restrictions on delaying relocation are observed among older people with families. Nevertheless, the inclination to migrate is likely to decrease due to government assistance, social support, and a strong connection to one’s homeland, all of which should be taken into account for future interventions.

#### 3.2.3. Economic factors.

**Income and income adequacy**: Both monthly income and perceived income adequacy were significant predictors. Interestingly, lower income was associated with a higher likelihood of wanting to leave the village, whereas perceptions of income adequacy showed a complex relationship, consistent with [74]. Lower income groups were more likely to want to move (p < 0.001, Exp(B) = 0.573 for “maybe” and Exp(B) = 1.735 for “yes”). The significant role of income in migration decisions aligns with economic theories of migration and PMT assumptions, such as those proposed by [75], which posit that individuals are more likely to move when they perceive better economic opportunities elsewhere. However, the complex relationship with income adequacy reflects findings from studies such as [74], which suggest that subjective perceptions of economic well-being can be as crucial as actual income in migration decisions. Furthermore, the political economy of EU-Egypt migration has been discussed to curb irregular flows to EU countries across the Mediterranean Sea by people seeking high-wage jobs that aren’t available in their domestic labour markets (Egypt) [23,76]. These studies confirmed that the Egyptian labor market can export skilled labor to the EU or other Arab countries by establishing training centers to enhance the skills of those people willing to migrate.

#### 3.2.4. Agricultural system factors.

**Agricultural engagement**: Working in the agricultural sector was negatively associated with the desire to move from villages (among landowners), though the association was not always significant (p = 0.053). This could indicate a strong attachment to agricultural livelihoods among those employed in this sector. These findings are consistent with the study of Gray et al., who found that individuals engaged in agriculture were less likely to migrate, as they were more attached to their land and agricultural activities [9]. However, we observed that the young rural people in the selected area were mainly willing to migrate due to the negative impacts of climate change and environmental stress on the agricultural sector, which puts this sector at high risk. Another study has discussed how households with strong agricultural livelihoods often exhibit lower migration rates, particularly in contexts where agriculture remains a viable livelihood strategy [77]. Most of the farmers in the selected area depend on agricultural production as their primary income source, where the shortage of water increases the salinity of the soil, decreasing the productivity and income of those farmers and forcing them to migrate (in case they don’t own land or their ownership is a small area).

**Changes in land productivity** or farming systems due to climate change and other socioeconomic factors push many people to migrate, prompting stakeholders to seek different employment opportunities in urban areas. This encourages rural-urban migration; this finding is consistent with the recent literature [40,41]. The recent literature revealed that rural people in Africa will face a decline in agricultural production due to the negative impacts of climate change and land degradation, and they will be the most affected and exposed to migration [78], especially in vulnerable areas like the study area (Fayoum). Unfortunately, most young people in the selected area are either willing to migrate to EU countries illegally or to move (temporarily or permanently) to find work in capital cities like Cairo or Alexandria, which decreases the labour supply during the seasonal demand for agricultural labour in this area. Salinity reduces soil fertility in the selected area, thereby lowering crop yields and forcing farmers to change their cropping patterns, ultimately decreasing agricultural income. This degradation compels farmers to abandon their land and seek better opportunities in urban areas [26]. Our findings align with recent research, which found that desertification in the Inner Mongolia (Northern China) region has resulted in large-scale rural displacement [20]. Also, in Bangladesh, riverbank erosion and soil salinity in coastal areas have forced many rural inhabitants to migrate to urban slums in Dhaka, highlighting the link between environmental degradation and urban poverty [16,22]. This highlights that increasing soil salinity leads to decreased productivity and income, and, subsequently, an increase in future migration willingness. The intervention should focus more on technologies and farming practices that enhance soil fertility under water scarcity and soil salinity, to reduce future migration flows.

#### 3.2.5. Perceptions of change.

**Perception of living conditions**: Individuals who believed that living conditions for farmers had improved over the last decade were less likely to consider moving (p = 0.033). This highlights the importance of perceived improvements in quality of life in retaining rural populations. This finding supports previous studies demonstrating that perceived improvements in local living conditions, particularly in rural areas, can reduce the likelihood of migration by enhancing satisfaction with local opportunities and quality of life [79]. The migration decision depends on the interaction between a set of variables, including migrants’ socioeconomic characteristics and environmental change, that should be considered in the design of interventions to tackle migration. The real example in this case is the national initiative in China (rural revitalization), which aims to support rural communities in reducing rural-urban migration and securing food products for local self-sufficiency [79].

Although some variables included in the current study have been statistically insignificant, e.g., marital status and ownership of agricultural land, these factors remain influential in understanding and explaining migration intention and decision-making, as confirmed in the literature [13,48,49]. Future studies could therefore investigate the interactions among these sets of variables across different regions to understand migration motivations.

### 3.3. Motivations and barriers of migration intention

#### 3.3.1. Motivations of migration.

The primary driver of migration is the availability of suitable job opportunities in another governorate, with 70.1% of respondents agreeing, ranking it first with a weighted percentage of 84.3%, as shown in **Table 3**. This result aligns with Radwan et al. (2022), who found that the lack of job opportunities in rural areas significantly pushes internal migration in Egypt [80]. Similarly, the pursuit of higher wages or salary increases ranked second (66.8%) and accounted for 83.0% of the weighted percentage, confirming that economic incentives play a crucial role in migration decisions [28]. The possibility of starting a project with higher income in destination areas ranked third (62.4%) and had a weighted percentage of 81.3%, reflecting the aspiration for better income-generating activities. This finding aligns with Abdelwahed et al. (2020), who reported that rural populations often seek self-employment opportunities as an alternative to low agricultural incomes [28]. Furthermore, the inadequacy of current income (52.9%) emerged as another significant driver, with a weighted percentage of 77.3%, consistent with Xu et al. (2020), who highlighted that low income is one of the primary factors motivating rural-to-urban migration [21].

**Table 3:** Weighted percentage for the migration motivations under land degradation.

Why do you want to change your current accommodation place?	Scale						Total marks	Weighted percentage%	Rank
	Agree		Neutral		Disagree				
	F	%	F	%	F	%			
To find a suitable job opportunity in another governorate	1249	70.1	220	12.3	313	17.6	4500	84.3%	1
To transfer my job to another governorate	893	50.1	425	23.8	464	26	3993	74.7%	8
To find a higher wage or a salary increase there	1191	66.8	271	15.2	320	18	4435	83.0%	2
To open a project with a higher income there	1112	62.4	346	19.4	324	18.2	4352	81.3%	3
The cost of living here is expensive	691	38.8	523	29.3	568	31.9	3687	69.0%	12
I can’t find a job in my village	857	48.1	434	24.4	491	27.6	3930	73.7%	11
My salary or monthly income in the village is not enough for me	943	52.9	472	26.5	367	20.6	4140	77.3%	4
I opened a project in the village, and its income is not good	633	35.5	461	25.9	688	38.6	3509	65.7%	13
I don’t know how to work more than one job in my village	859	48.2	456	25.6	467	26.2	3956	74.0%	10
To find clean housing with reasonable prices in another governorate	876	49.2	436	24.5	470	26.4	3970	74.3%	9
The educational services in the village are not good	865	48.5	493	27.7	424	23.8	4005	75.0%	7
The health services in the village are not good	864	48.5	514	28.8	404	22.7	4024	75.3%	6
The entertainment services in the village are not good	937	52.6	438	24.6	407	22.8	4094	76.7%	5

$\text{Weighted percentage}\%=\frac{\text{Total marks}}{\text{N}*\text{3}}*\text{1}\text{0}\text{0}$, where the sample size is (N=1782)

The quality of public services also affects migration decisions. Poor educational services (48.5%) and health services (48.5%) in villages ranked among the top reasons, with weighted percentages of 75.0% and 75.3%, respectively. These results are supported by Selod et al. (2021), who found that inadequate infrastructure and services in rural regions push many families to seek better living conditions in urban areas [41].

#### 3.3.2. Barriers to migration.

Despite the desire to migrate, several obstacles prevent rural residents from moving, as shown in **Table 4**. The most significant barrier was the difficulty of moving to a new place, reported by 54.1% of respondents, with a weighted percentage of 78.0%. This finding aligns with Selod et al. (2021) observation that rural households face logistical and financial challenges when relocating [41]. Family objection (54.2%) and high moving costs (52.7%) were the next most prominent obstacles, both ranked third with a weighted percentage of 76.3%. These results are consistent with Zohry (2005), who found that family ties and financial constraints are key barriers to migration in rural Egypt [29].

**Table 4:** Weighted percentage for the migration barriers under land degradation.

What might be an obstacle for you to leave your village and work or live in another province?	Scale						Total marks	Weighted percentage%	Rank
	agree		Neutral		Disagree				
	F	%	F	%	F	%			
Difficulty of moving to the new place	964	54.1	452	25.4	366	20.5	4162	78.0%	1
Lack of suitable housing in the new place	919	51.6	482	27	381	21.4	4102	76.7%	2
Cost of moving	940	52.7	425	23.8	417	23.4	4087	76.4%	3
Family objection	966	54.2	360	20.2	456	25.6	4074	76.2%	4
I might feel isolated and alone in the new place	916	51.4	447	25.1	419	23.5	4061	76.0%	5
Lack of friends and relatives in the new place	896	50.3	465	26.1	421	23.6	4039	75.7%	6
Distance of travel	831	46.6	517	29	434	24.4	3961	74.0%	7
Lack of continuous transportation to the new place	827	46.4	498	27.9	457	25.6	3934	73.7%	8

Other significant obstacles included the lack of suitable housing (51.6%) and the fear of social isolation (51.4%), with weighted percentages of 76.7% and 76.0%, respectively. These findings align with those of Xiang et al. (2016), who reported that rural migrants often struggle to integrate into new communities due to a lack of social networks and affordable housing [54].

#### 3.3.3. Motivations and barriers behind migration decisions.

We estimated the impact of respondents’ demographic and economic characteristics on their willingness to relocate using PMT assumptions, highlighting the motivations and barriers behind migration decisions. Therefore, we constructed a composite variable for motivation and another for barriers, each with three levels (high, medium, and low); we set medium as the reference in our estimations. Using this variable, we estimated respondents’ perceived intention to relocate due to environmental change and low agricultural income in the selected area, based on their socioeconomic characteristics. The determinants that influence whether individuals are motivated to migrate or face barriers to relocation are summarized in **Table 5 and Table 6**. The results reveal that both demographic and economic characteristics significantly affect migration intentions, although the magnitudes and directions of these effects differ across the motivation and barrier models.

**Table 5:** Overall effect of the main variables on migration intention (motivation and barriers).

Effect	Motivation			Barriers		
	Model Fitting Criteria (−2 Log Likelihood of Reduced Model)	Chi-Square	df	Model Fitting Criteria (−2 Log Likelihood of Reduced Model)	Chi-Square	df
Intercept	1845.308^a^	0.000		1935.884^a^	0.000	
Age	1873.284	27.976^***^	2	1935.884^a*^	0.000	2
Gender	1854.039	8.732^**^	2			
Educational Status	1865.923	20.615^**^	10			
Job	1863.314	18.006^**^	8			
Income Adequacy	1867.576	22.268^***^	4			
Monthly income				1944.038^**^	8.154	2
Marital Status				1950.504^**^	14.620	6
Change in land productivity.				1951.118^***^	15.234	4
Change in village conditions now compared to 10 years ago.				1944.920^*^	9.036	4
Model Fitting (−2 Log Likelihood)	1845.31	191.46^***^	62	1935.88	129.97^***^	62
	**Pseudo R-Square**					
Cox and Snell	0.167			0.117		
Nagelkerke	0.195			0.136		
McFadden	0.094			0.063		

*** Significant at p < 0.01 ** Significant at p < 0.05 * Significant at p < 0.10

The chi-square statistic is the difference in the −2 log-likelihoods between the final and reduced models. The reduced model is formed by omitting an effect from the final model. The null hypothesis is that all parameters of that effect are 0.

a. This reduced model is equivalent to the final model because omitting the effect does not increase the degrees of freedom.

**Table 6:** Significant parameter estimates for motivation and barriers models.

Parameter Estimates	Motivation				Barriers			
	(1) Low		(3) High		(1) Low		(3) High	
	Std. Er.	Exp(B)	Std. Er.	Exp(B)	Std. Er.	Exp(B)	Std. Er.	Exp(B)
Intercept	1.027		0.694		0.963			
Age	0.009	1.046***			0.009	1.023**		
[Income adequacy=1] Not enough	0.306	0.520**	0.216	1.734***				
[Income adequacy=2] Enough	0.257	0.490***						
Agriculture’s share of income			0.276	0.590*				
Monthly income			0.000	1.000*			0.000	1.0***
[Gender=1] Females			0.266	1.589*				
[Educational Status=1] Illiterate			0.304	0.497**				
[Educational Status=4] University and post-university			0.248	0.424***				
[Educational Status=5] Institute (above intermediate)			0.368	0.349***				
[Job=3] government employee and work in agriculture			0.373	0.513*				
[Job= 7] Private sector and work in agriculture	0.454	0.328**						
Agricultural share of total income					0.387	0.357***		
[Changes in land productivity =2] As it was 10 years ago					0.303	2.228***		
[Living conditions of the farmer now compared to 10 years ago= 1] Now worse					0.268	0.643*	0.196	0.660**
[Living conditions of the farmer now compared to 10 years ago= 2] As they were 10 years ago							0.252	0.598**
[Change in village conditions now compared to 10 years ago =2.00] As it was 10 years ago					0.321	0.541*	0.216	0.635**
[Agri-land ownership =0] No							0.693	3.815*
[Changes in land productivity =1] Now, less than 10 years ago							0.210	1.678**
[Changes in land productivity =2] As it was 10 years ago							0.225	1.456*

a. The reference category is: 2.

b. This parameter is set to zero because it is redundant.

*** Values are significant at P = 0.01, ** Values are significant at P = 0.05, * Values are significant at P = 0.1

**Model Fit:** Both models demonstrated acceptable goodness-of-fit. The Motivation model reported −2 Log Likelihood = 1845.31 and Chi-Square = 191.46 (df = 62, p < 0.001), with Pseudo R² values of Cox & Snell = 0.167, Nagelkerke = 0.195, and McFadden = 0.094. The Obstacles model yielded −2 Log Likelihood = 1935.88 and Chi-Square = 129.97 (df = 62, p < 0.001), with Pseudo R² values of Cox & Snell = 0.117, Nagelkerke = 0.136, and McFadden = 0.063. These results indicate a moderate explanatory capacity, appropriate for social-science datasets.

Demographic Factors, Age emerged as a significant predictor of migration intention in both models. In the *Motivation* model, the odds ratio (Exp(B) = 1.046, p < 0.01) indicates that as respondents grow older, their likelihood of considering migration slightly increases. Similarly, in the *Obstacles* model, Exp(B) = 1.023 (p < 0.05) shows that older individuals perceive fewer barriers to migration. These results contrast with earlier evidence suggesting that younger people are generally more mobile and willing to migrate for better opportunities. At the same time, older individuals tend to remain in their communities because of established livelihoods and social networks [28,68]. However, in rural Egypt, age can also reflect financial independence and accumulated capital, which may facilitate migration rather than deter it. Gender significantly influenced migration obstacles. In the *Obstacles* model, females were more likely to experience migration motivation (Exp(B) = 1.589, p < 0.10). This pattern aligns with studies highlighting that women in rural areas often view migration as a pathway to greater autonomy and improved employment opportunities [81,82]. Although men usually maintain stronger ties to land ownership and agricultural work, limited local opportunities for women often drive them toward urban or overseas migration [83]. These findings emphasize that gendered access to resources and employment remains a central determinant of migration behavior in rural Egypt.

Education was a significant determinant across several categories in the *Obstacles* model. Respondents with university or post-university education exhibited a lower Exp(B) = 0.424 (p < 0.01), while those with intermediate-level qualifications (institutes) recorded Exp(B) = 0.349 (p < 0.01). Conversely, illiterate respondents showed Exp(B) = 0.497 (p < 0.05), suggesting that lower levels of education are associated with greater obstacles to migration. These results indicate that higher educational attainment facilitates migration decisions, consistent with findings that education enhances awareness of opportunities and mobility [81]. Similar trends have been reported among Egyptian youth, where educated individuals pursue migration for professional development or improved income prospects [28].

Economic Factors include income and income adequacy, with income adequacy being significant in both models. In the *Motivation* model, households reporting “not enough” income had Exp(B) = 0.520 (p < 0.05), while those reporting “enough” income recorded Exp(B) = 0.490 (p < 0.01). In the *Obstacles* model, inadequate income substantially increased the likelihood of migration (Exp(B) = 1.734, p < 0.01). These findings highlight that both objective and perceived income conditions influence migration, consistent with Gray et al. (2012) and Restelli (2023) [16,83]. Monthly income was also significant (Exp(B) = 1.000, p < 0.01), indicating that higher income slightly reduces the probability of facing migration obstacles. Such results align with classical migration theory (Todaro, 1969), which links income disparities and job opportunities to mobility decisions [75]. In Egypt, low-income households often rely on migration to diversify income sources, while skilled workers are pulled abroad by higher wages in the EU and Gulf countries [84].

Agricultural Engagement: Employment in agriculture and its share of household income negatively influenced migration intention. In the Obstacles model, the agricultural income shares yielded Exp(B) = 0.357 (p < 0.01), implying that stronger agricultural dependence reduces migration propensity. Similarly, employment in private agricultural work (Exp(B) = 0.328, p < 0.05) limited migration motivation. These results are consistent with Gray et al. (2012), who observed that individuals closely tied to farming are less likely to migrate due to attachment to land and local livelihoods [16]. However, environmental pressures—such as declining soil fertility and water scarcity—can offset this stability, especially among younger farmers [16,84].

Changes in Land Productivity: The Obstacles model showed that changes in land productivity significantly influenced migration barriers. Respondents reporting lower productivity than 10 years ago were more likely to face migration pressures (Exp(B) = 1.678, p < 0.05). At the same time, those whose productivity remained stable also exhibited a moderate increase in migration likelihood (Exp(B) = 1.456, p < 0.10). These findings suggest that any stagnation or decline in agricultural productivity acts as a push factor, heightening migration intentions. Conversely, productivity improvements strengthen local economic resilience and reduce migration risks. Similar evidence has been documented in Egypt and Bangladesh, where soil salinity, desertification, and land degradation have undermined rural livelihoods and triggered out-migration [16]. Perceived improvements in living standards and village conditions significantly lowered migration obstacles. For example, respondents reporting worse living conditions recorded Exp(B) = 0.660 (p < 0.05), while those perceiving conditions as unchanged showed Exp(B) = 0.598 (p < 0.05). These results highlight the importance of subjective well-being and satisfaction in migration decisions. Similar findings in China’s Rural Revitalization program show that improving infrastructure and rural services reduces out-migration [77]. Overall, demographic (age, gender, education), economic (income, income adequacy), agricultural (engagement and productivity), and perceptual factors jointly determine migration intentions in rural Egypt. The results confirm that migration decisions stem from intertwined socio-economic and environmental dynamics rather than single determinants [16,28,84].

Overall, the analysis of migration intentions in Fayoum can be clearly understood within the Protection Motivation Theory (PMT) framework. Agricultural land degradation significantly increases threat appraisal by raising farmers’ perceived severity and vulnerability, as declining productivity and livelihood insecurity heighten the risk of staying. At the same time, socio-economic resources such as education, remittances, and land assets serve as coping appraisal factors, shaping households’ capacity to adapt locally or migrate successfully. The identified drivers of migration—such as environmental stress and unemployment—correspond to dimensions of threat appraisal, while the barriers—including limited resources, family obligations, and lack of skills—reflect coping constraints. Taken together, these findings demonstrate that migration decisions emerge from the interaction between threat and coping appraisals. This provides an integrated explanation of why some households choose to migrate. In contrast, others remain, underscoring the value of PMT as a guiding theoretical framework for analyzing environmentally induced migration in developing-country contexts.

## 4. Conclusions and policy implications

The complex interrelationships among population dynamics, agricultural systems, and environmental change pose significant challenges for understanding the drivers of migration. Within the Protection Motivation Theory (PMT) framework, our findings show that the two principal PMT dimensions—threat appraisal and coping appraisal—have now been explicitly operationalized and systematically linked to the empirical analysis. Threat appraisal is represented through respondents’ perceptions of declining land productivity, livelihood insecurity, worsening living conditions, and environmental stress associated with agricultural land degradation. Coping appraisal is reflected through socioeconomic resources such as education, income adequacy, agricultural assets, migration opportunities, and adaptive capacity.

While absolute income was a significant predictor, perceptions of income adequacy showed a more nuanced association with migration intentions, suggesting a mediating role for subjective well-being. These results affirm the explanatory sufficiency and robustness of PMT in this context. Furthermore, they underscore the need to integrate socio-economic and institutional dimensions into the PMT framework to build a comprehensive model. Consequently, effective intervention strategies should aim to reduce threat appraisal by implementing sustainable land and water management, poverty alleviation programs, and enhanced rural services. Concurrently, coping appraisal can be strengthened through expanded access to education and vocational training, as well as initiatives that support women’s entrepreneurship. Such integrated approaches would not only mitigate the drivers of migration but also foster inclusive and sustainable rural development.

The policy implications from the empirical findings of both models emphasize the need to design integrated rural development policies that directly address the root causes of migration in Egypt’s rural regions. Since migration intention is powerfully shaped by demographic, economic, and agricultural factors, interventions should prioritize youth employment creation, women’s empowerment, and agricultural modernization. Policies that enhance rural education, vocational training, and entrepreneurship, particularly for young and educated individuals, can convert migration pressure into local productivity gains.

Strengthening income adequacy through diversified livelihoods, access to credit, and value-added agribusiness initiatives can mitigate economic push factors. Moreover, the significant influence of land productivity and living conditions underscores the need to invest in climate-smart agriculture, irrigation efficiency, and soil salinity management to stabilize agricultural income and reduce migration driven by environmental degradation. Gender-sensitive measures are equally critical: supporting women’s land ownership, improving their access to markets, and promoting their participation in decision-making can reduce the gendered drivers of migration. Overall, adopting a comprehensive rural revitalization framework, like China’s model, would help retain rural populations by improving infrastructure, public services, and local governance, thereby ensuring that migration becomes a choice driven by opportunity rather than necessity. As a limitation of the current study, some diagnostic procedures, including formal testing of the Independence of Irrelevant Alternatives (IIA) assumption and robustness checks for multicollinearity, were not fully explored in the original analysis and should be considered in future studies to further strengthen model reliability.

## Supporting information

S1 Appendix Selected villages in the *Itsa* districts. (DOCX)

S2 Appendix Descriptive statistics of the sample. (DOCX)

S3 Appendix Farming system characteristics and migration intention of the selected respondents N= (1782). (DOCX)
