# Supplementary material for: Agricultural land degradation consequences as a migration driver in Egypt
Source: PLoS One. 2026 Jul 17;21(7):e0353721. doi: 10.1371/journal.pone.0353721 (PMC13379038; doi:10.1371/journal.pone.0353721)
Supplement: S1 Appendix — (DOCX) [file pone.0353721.s001.docx]

**Appendix**

**Table A.1:** Selected villages in the *Itsa* districts

| **Cluster** | **Villages** | **Total population** | **Selected respondents (N)** | **Selected sample (%)** |
| --- | --- | --- | --- | --- |
| Cluster 1 (Desert Hinterland) | *Qalhana, Qolamsha, Qasr El-Basil, and Tatoun* | 166878 | 431 | 24.77% |
| Cluster 2 (*Bahr Youssef* Delta) | *Gerdo, Matoul, and Abousir* | 197868 | 488 | 28.05% |
| Cluster 3 (*Bahr El-Nazla* and *Bahr El-Banat* lands) | Abogendeer and Menyet El-Heet | 183884 | 430 | 24.71% |
| Cluster 4 (*El-Gharq El-Soltany* Basin) | *Shidmoh, El-Gharq, El-Hagar*, and *Anak* | 170524 | 391 | 22.47% |
| Total |  |  |  | 100 % |
