# Supplementary material for: Agricultural land degradation consequences as a migration driver in Egypt
Source: PLoS One. 2026 Jul 17;21(7):e0353721. doi: 10.1371/journal.pone.0353721 (PMC13379038; doi:10.1371/journal.pone.0353721)
Supplement: S2 Appendix — (DOCX) [file pone.0353721.s002.docx]

**Appendix**

**Table A.2:** Descriptive statistics of the sample

| **Sample Characteristics (n= 1782)** | | **F** | **% N** |
| --- | --- | --- | --- |
| **Gender** | Female | 303 | 17.00% |
|  | Male | 1479 | 83.00% |
| **Rural Unit** | *Qalhanah* | 98 | 5.50% |
|  | *Qalamshah* | 128 | 7.20% |
|  | *AbuJandir* | 189 | 10.60% |
|  | *AlHajar* | 162 | 9.10% |
|  | *Tutun* | 132 | 7.40% |
|  | *Minyat_AlHayt* | 251 | 14.10% |
|  | *Gerdo* | 247 | 13.90% |
|  | *AlGharq* | 121 | 6.80% |
|  | *Mutul* | 146 | 8.20% |
|  | *AbuSir-Difinnu* | 107 | 6.00% |
|  | *Shidmuh* | 117 | 6.60% |
|  | *Qasr_AlBasil* | 84 | 4.70% |
| **Marital Status** | under 18 years old | 39 | 2.20% |
|  | Single (never married) | 426 | 23.90% |
|  | Married | 1228 | 68.90% |
|  | Divorced | 30 | 1.70% |
|  | Widower | 59 | 3.30% |
| **Educational Status** | Illiterate | 377 | 21.70% |
|  | Reads and writes | 299 | 17.20% |
|  | Basic | 98 | 5.60% |
|  | Secondary | 497 | 28.60% |
|  | Institute | 106 | 6.10% |
|  | University and post-university | 363 | 20.90% |
| **Do you work in agriculture or elsewhere?** | I work in agriculture only | 472 | 26.50% |
|  | Government worker, and I work in agriculture | 50 | 2.80% |
|  | Government worker, and I do not work in agriculture | 59 | 3.30% |
|  | Government employee (specialist - teacher - pharmacist - doctor - etc.), and does not work in agriculture | 67 | 3.70% |
|  | Government employee, and I work in agriculture | 91 | 5.10% |
|  | Private sector (trader, shop owner, driver, etc.), and I do not work in agriculture | 283 | 15.90% |
|  | Private sector, working in agriculture | 326 | 18.30% |
|  | I do not work | 434 | 24.40% |
| **Do you own agricultural land?** | No | 738 | 41.40% |
|  | Yes | 1044 | 58.60% |
