# Supplementary material for: Agricultural land degradation consequences as a migration driver in Egypt
Source: PLoS One. 2026 Jul 17;21(7):e0353721. doi: 10.1371/journal.pone.0353721 (PMC13379038; doi:10.1371/journal.pone.0353721)
Supplement: S3 Appendix — (DOCX) [file pone.0353721.s003.docx]

**Appendix**

**Table A.3:** Farming system Characteristics and migration intention of the selected respondents N= (1782)

| **Items** | **Responses** | **N** | **%** |
| --- | --- | --- | --- |
| **Has the area of land you own now changed from 10 years ago?** | The area is now less than 10 years ago | 248 | 22.9% |
|  | The area has not changed | 502 | 46.3% |
|  | The area is now larger than 10 years ago. | 335 | 30.9% |
| **Has the area of ​​land you cultivate changed now compared to 10 years ago?** | The area is now less than 10 years ago | 309 | 22.1% |
|  | The area has not changed | 744 | 53.3% |
|  | The area is now larger than 10 years ago. | 343 | 24.6% |
| **How about the rice cultivation in your crop pattern?** | Within the crop structure of only 10 years | 171 | 60.4% |
|  | Within the current crop structure and for 10 years | 66 | 23.3% |
|  | Within the current crop composition only | 46 | 16.3% |
| **How about the sugar beet cultivation in your crop pattern?** | Within the crop structure of only 10 years | 158 | 35.3% |
|  | Within the current crop structure and for 10 years | 165 | 36.9% |
|  | Within the current crop composition only | 124 | 27.7% |
| **Has there been a change in the productivity of the land you cultivate?** | Productivity is now less than 10 years ago | 503 | 35.8% |
|  | Productivity has not changed | 516 | 36.8% |
|  | Productivity is now larger than 10 years ago. | 385 | 27.4% |
| **Total income (monthly)** | less than 2000 LE | 419 | 23.5% |
|  | 2000 - 4000 | 774 | 43.4% |
|  | 4000- 6000 | 378 | 21.2% |
|  | more than 6000 | 211 | 11.8% |
| **Income adequacy** | Not enough | 624 | 35.0% |
|  | Somewhat enough | 684 | 38.4% |
|  | Enough | 474 | 26.6% |
| **What do you think of the current state of agricultural production?** | Now worse | 788 | 44.2% |
|  | As it was 10 years ago | 321 | 18.0% |
|  | Now better | 673 | 37.8% |
| **What do you think about the living conditions of the farmer now compared to 10 years ago?** | Now worse | 952 | 53.4% |
|  | As it was 10 years ago | 257 | 14.4% |
|  | Now better | 573 | 32.2% |
| **What do you think of the village now compared to 10 years ago?** | Now worse | 588 | 33.0% |
|  | As it was 10 years ago | 295 | 16.6% |
|  | Now better | 899 | 50.4% |
| **Have you ever changed your place of residence (for 6 months or more)?** | No | 1485 | 83.3% |
|  | Yes | 297 | 16.7% |
| **Have you worked outside Fayoum Governorate (for 6 months or more)?** | No | 1079 | 60.5% |
|  | Yes | 703 | 39.5% |
| **Do you want to change your place of residence and leave the village?** | No | 805 | 45.2% |
|  | Maybe | 577 | 32.4% |
|  | Yes | 400 | 22.4% |
| **I plan to change residence to ….** | Greater Cairo | 314 | 40.2% |
|  | Alexandria | 47 | 6.0% |
|  | Cities of the Suez Canal, Sinai and the Red Sea | 31 | 4.0% |
|  | Delta Governorates | 27 | 3.5% |
|  | Upper Egypt Governorates | 12 | 1.5% |
|  | Fayoum City | 120 | 15.4% |
|  | Outside Egypt | 230 | 29.4% |
| **Do you want to change your job location or look for work outside your village?** | No | 681 | 38.2% |
|  | Maybe | 512 | 28.7% |
|  | Yes | 589 | 33.1% |
| **Change of job location or look for work to** | Greater Cairo | 303 | 37.5% |
|  | Alexandria | 48 | 5.9% |
|  | Cities of the Suez Canal, Sinai and the Red Sea | 28 | 3.5% |
|  | Delta Governorates | 29 | 3.6% |
|  | Upper Egypt Governorates | 9 | 1.1% |
|  | Fayoum City | 94 | 11.6% |
|  | Outside Egypt | 296 | 36.7% |
| **If legal travel is not available, is it possible to travel illegally?** | No | 1138 | 63.9% |
|  | Maybe | 275 | 15.4% |
|  | Yes | 369 | 20.7% |
| **If you leave the village and work in another governorate, you will take your family with you.** | I won't take them at all | 439 | 24.6% |
|  | I might take them, and I might not | 323 | 18.1% |
|  | I'll take them after I settle in | 123 | 6.9% |
|  | I'll take some of them to help me there | 386 | 21.7% |
|  | I'll take them all, of course | 511 | 28.7% |
| **If you work in another governorate, will you change your place of residence on your official ID?** | I won't change it at all | 431 | 24.2% |
|  | I won't change it unless I have to | 357 | 20.0% |
|  | I might change it, and I might not | 377 | 21.2% |
|  | I will change it after it settles | 337 | 18.9% |
|  | I will change it of course | 280 | 15.7% |
| **If you moved to another governorate and liked the way you lived there, would you advise others to move there?** | No | 360 | 20.2% |
|  | Maybe | 800 | 44.9% |
|  | Yes | 622 | 34.9% |
