# Supplementary material for: Agricultural land degradation consequences as a migration driver in Egypt
Source: PLoS One. 2026 Jul 17;21(7):e0353721. doi: 10.1371/journal.pone.0353721 (PMC13379038; doi:10.1371/journal.pone.0353721)
Supplement: Attachment — Submitted filename: Reviewers Comments.pdf [file pone.0353721.s004.pdf]

Agricultural Land Degradation Consequences as a Migration Driver in Developing Countries

Reviewer Comments

This manuscript explores the role of Agricultural Land Degradation (ALD), especially soil salinity, as a driver of rural-urban migration in Egypt. The topic is timely and important, contributing to the growing literature on climate-induced migration in the Global South. The manuscript is based on a robust sample and applies Protection Motivation Theory (PMT) and a multinomial logistic regression model to analyze migration intentions.

However, the paper would benefit from some moderate revisions to improve conceptual clarity, methodological rigor, and academic writing. Below are detailed comments.

Title: Consider making it more specific by including "Egypt" as the country case study.

Abstract: Remove redundancy (e.g., repeating "migration willingness") and clarify the novel contribution.

Introduction: Better delineate the knowledge gap the paper addresses. Please clarify better how this study extends existing literature beyond identifying the problem. Eg….While existing research has explored the relationship between economic factors and migration, few studies have specifically analyzed how ALD influences migrants' decisions, particularly in terms of migration timing. [Reference these works] Most prior research has focused on general migration drivers, whereas our study sheds light on the more specific…[Reference these works]

Literature Review: Whilst the literature engages with a broad set of sources from multiple geographies, it is too descriptive. More analytical synthesis is needed—compare, contrast, and critique existing works. Include more regionally comparative insights, especially from other North African or Middle Eastern contexts.

5. Theoretical Framework: PMT is a novel and creative theoretical lens for migration, but Better justify why PMT is appropriate in this migration context; originally used in health behavior studies. Consider complementing PMT with Livelihoods Vulnerability Framework or Push-Pull models to enhance explanatory power.

6. Methodology: More detail is needed on how variables were operationalized (e.g., how is "migration intention" coded?). Consider adding a table summarizing variable definitions and expected relationships. Explain potential biases and limitations, e.g., is Itsa District representative of broader Egypt? Include details on ethical clearance and informed consent.

Results and Discussion: Well-structured and includes strong descriptive and inferential statistics.

Writing and Language: The manuscript needs significant language polishing. Some sentences are overly long or grammatically incorrect. Examples: “the capital Cairo (40.2%) and international destinations (29.4%) were the most desired.” → “Among those willing to migrate, Cairo (40.2%) and international destinations (29.4%) were preferred.” Use of commas, conjunctions, and passive voice should be corrected throughout.

Policy and Practical Implications: The discussion on policy implications is promising but too brief and generic. Expand on Targeted adaptation strategies, Links to SDGs, especially SDG 13 (Climate Action) and SDG 10 (Reduced Inequalities), Role of government, NGOs, and international donors in mitigating ALD.

Conclusion: Whilst the conclusion summarizes findings clearly, consider distilling 2–3 key takeaways for policymakers and academics. Include a brief note on future research directions, e.g., longitudinal studies or gender-specific analyses.
