# Supplementary material for: Agricultural land degradation consequences as a migration driver in Egypt
Source: PLoS One. 2026 Jul 17;21(7):e0353721. doi: 10.1371/journal.pone.0353721 (PMC13379038; doi:10.1371/journal.pone.0353721)
Supplement: Attachment — Submitted filename: PONE-D-25-21257_R1_Florence.pdf [file pone.0353721.s007.pdf]

PLOS ONE

Agricultural Land Degradation Consequences as a Migration Driver in Egypt

--Manuscript Draft--

**Manuscript Number:**	PONE-D-25-21257R1
**Article Type:**	Research Article
**Full Title:**	Agricultural Land Degradation Consequences as a Migration Driver in Egypt
**Short Title:**	Land Degradation and Migration
**Corresponding Author:**	Abdelrahman Ali<br>Fayoum University Faculty of Agriculture<br>Fayoum, Other EGYPT
**Keywords:**	Soil Restoration; Environmental Stresses; Rural-Urban Migration; Protection Motivation Theory; Migration Stream; Rural-urban Displacement
**Abstract:**	Environmental migration is a global phenomenon where increasing soil salinity leads to decreased productivity and income, subsequently increasing migration intention (MI) in the affected regions. This study investigates how agricultural land degradation (ALD), particularly soil salinity, reduces productivity and influences MIs in Egypt. Employing Protection Motivation Theory (PMT) as a framework, we analyzed primary data from 1,782 respondents using a multinomial logistic model. The empirical findings revealed that socioeconomic characteristics, agricultural engagement, and ALD significantly shape and determine MI. Furthermore, over 35.8% of respondents reported a decline in land productivity in the past decade due to environmental change, with 22.4% actively seeking relocation. Among those willing to migrate, the capital Cairo (40.2%) and international destinations (29.4%) were the most desired. Additionally, gender, age, and income adequacy were identified as crucial predictors in migration dynamics. The findings challenge conventional demographic assumptions by revealing a significant propensity to migrate among older populations and women, groups often considered less mobile. The primary motivations (employment and income) are directly exacerbated by ecological pressures like declining land productivity. Therefore, effective policy must address this nexus to reduce threat appraisal by implementing sustainable land and water management and investing in both environmental adaptation (e.g., soil restoration) and human capital (e.g., education, local economic opportunities) to mitigate displacement pressures and foster sustainable rural resilience.
**Order of Authors:**	Muhammad Hussien
	Abdelrahman Ali
	Yanwen Tan, Professor
	Mohamed Abdelhameed
**Response to Reviewers:**	We attached file. Please check
**Additional Information:**	
**Question**	**Response**
**Financial Disclosure**<br><br>Enter a financial disclosure statement that describes the sources of funding for the work included in this submission. Review the submission guidelines for detailed requirements. View published research articles from *PLOS ONE* for specific examples.	Yes

Powered by Editorial Manager® and ProduXion Manager® from Aries Systems Corporation

This statement is required for submission and **will appear in the published article** if the submission is accepted. Please make sure it is accurate.<br><br>**Funded studies**<br>Enter a statement with the following details:<br>• Initials of the authors who received each award<br>• Grant numbers awarded to each author<br>• The full name of each funder<br>• URL of each funder website<br>• Did the sponsors or funders play any role in the study design, data collection and analysis, decision to publish, or preparation of the manuscript?<br><br>Did you receive funding for this work?	
Please add funding details.<br>as follow-up to "**Financial Disclosure**<br><br>Enter a financial disclosure statement that describes the sources of funding for the work included in this submission. Review the submission guidelines for detailed requirements. View published research articles from *PLOS ONE* for specific examples.<br><br>This statement is required for submission and **will appear in the published article** if the submission is accepted. Please make sure it is accurate.<br><br>**Funded studies**<br>Enter a statement with the following details:<br>• Initials of the authors who received each award<br>• Grant numbers awarded to each author<br>• The full name of each funder<br>• URL of each funder website<br>• Did the sponsors or funders play any role in the study design, data collection and analysis, decision to publish, or preparation of the manuscript?<br><br>Did you receive funding for this work?"	This work was supported by The National Natural Science Foundation of China Project: The Economic Transformation and the Development of Regional Agricultural Products Value Chain of ASEAN and China (71961147002).

Please select the country of your main research funder (please select carefully as in some cases this is used in fee calculation).<br>as follow-up to "**Financial Disclosure**<br><br>Enter a financial disclosure statement that describes the sources of funding for the work included in this submission. Review the submission guidelines for detailed requirements. View published research articles from *PLOS ONE* for specific examples.<br><br>This statement is required for submission and **will appear in the published article** if the submission is accepted. Please make sure it is accurate.<br><br>**Funded studies**<br>Enter a statement with the following details:<br>• Initials of the authors who received each award<br>• Grant numbers awarded to each author<br>• The full name of each funder<br>• URL of each funder website<br>• Did the sponsors or funders play any role in the study design, data collection and analysis, decision to publish, or preparation of the manuscript?<br><br>Did you receive funding for this work?"	CHINA - CN
**Competing Interests**<br><br>Use the instructions below to enter a competing interest statement for this submission. On behalf of all authors, disclose any competing interests that could be perceived to bias this work—acknowledging all financial support and any other relevant financial or non-financial competing interests.<br><br>This statement is required for submission and **will appear in the published article** if the submission is accepted. Please make sure it is accurate and that any funding	Each named author has substantially contributed to conducting the underlying research and drafting this manuscript. Additionally, to the best of our knowledge, the named authors have no conflict of interest, financial or otherwise.

sources listed in your Funding Information later in the submission form are also declared in your Financial Disclosure statement.<br><br>View published research articles from *PLOS ONE* for specific examples.<br><br>**NO authors have competing interests**<br><br>Enter: *The authors have declared that no competing interests exist.*<br><br>**Authors with competing interests**<br><br>Enter competing interest details beginning with this statement:<br><br>*I have read the journal's policy and the authors of this manuscript have the following competing interests: [insert competing interests here]*<br><br>* typeset	
**Ethics Statement**<br><br>Enter an ethics statement for this submission. This statement is required if the study involved:<br><br>• Human participants<br>• Human specimens or tissue<br>• Vertebrate animals or cephalopods<br>• Vertebrate embryos or tissues<br>• Field research<br><br>Write "N/A" if the submission does not require an ethics statement.<br><br>General guidance is provided below. Consult the submission guidelines for detailed instructions. **Make sure that all information entered here is included in the Methods section of the manuscript.**	Our Data has analyzed anonymously

**Format for specific study types**<br><br>**Human Subject Research (involving human participants and/or tissue)**<br>• Give the name of the institutional review board or ethics committee that approved the study<br>• Include the approval number and/or a statement indicating approval of this research<br>• Indicate the form of consent obtained (written/oral) or the reason that consent was not obtained (e.g. the data were analyzed anonymously)<br><br>**Animal Research (involving vertebrate animals, embryos or tissues)**<br>• Provide the name of the Institutional Animal Care and Use Committee (IACUC) or other relevant ethics board that reviewed the study protocol, and indicate whether they approved this research or granted a formal waiver of ethical approval<br>• Include an approval number if one was obtained<br>• If the study involved *non-human primates*, add *additional details* about animal welfare and steps taken to ameliorate suffering<br>• If anesthesia, euthanasia, or any kind of animal sacrifice is part of the study, include briefly which substances and/or methods were applied<br><br>**Field Research**<br><br>Include the following details if this study involves the collection of plant, animal, or other materials from a natural setting:<br>• Field permit number<br>• Name of the institution or relevant body that granted permission	
**Data Availability**<br><br>Authors are required to make all data underlying the findings described fully available, without restriction, and from the time of publication. PLOS allows rare exceptions to address legal and ethical concerns. See the PLOS Data Policy and FAQ for detailed information.	Yes - all data are fully available without restriction

Powered by Editorial Manager® and ProduXion Manager® from Aries Systems Corporation

A Data Availability Statement describing where the data can be found is required at submission. Your answers to this question constitute the Data Availability Statement and **will be published in the article**, if accepted.<br><br>**Important:** Stating 'data available on request from the author' is not sufficient. If your data are only available upon request, select 'No' for the first question and explain your exceptional situation in the text box.<br><br>Do the authors confirm that all data underlying the findings described in their manuscript are fully available without restriction?	
**Describe where the data may be found in full sentences. If you are copying our sample text, replace any instances of XXX with the appropriate details.**<br><br>• If the data are **held or will be held in a public repository**, include URLs, accession numbers or DOIs. If this information will only be available after acceptance, indicate this by ticking the box below. For example: *All XXX files are available from the XXX database (accession number(s) XXX, XXX.*).<br>• If the data are all contained **within the manuscript and/or Supporting Information files**, enter the following: *All relevant data are within the manuscript and its Supporting Information files.*<br>• If neither of these applies but you are able to provide **details of access elsewhere**, with or without limitations, please do so. For example:<br><br>*Data cannot be shared publicly because of [XXX]. Data are available from the XXX Institutional Data Access / Ethics Committee (contact via XXX) for researchers who meet the criteria for access to confidential data.*<br><br>*The data underlying the results presented in the study are available from (include the name of the third party*	We attached the data file as a supplementary

and contact information or URL).<br>• This text is appropriate if the data are owned by a third party and authors do not have permission to share the data.<br><br>* typeset	
Additional data availability information:	Tick here if the URLs/accession numbers/DOIs will be available only after acceptance of the manuscript for publication so that we can ensure their inclusion before publication.

Powered by Editorial Manager® and ProduXion Manager® from Aries Systems Corporation

Manuscript

Click here to access/download;Manuscript;Green Manuscript PONE-D-25-21257.docx

Type of the Paper (Article)

Agricultural Land Degradation Consequences as a Migration Driver in Egypt

Muhammad Hussien[1†], Abdelrahman Ali[2, 3*†], Yanwen Tan[2*], Mohamed Abdelhameed[3]

[1] Department of Geography, Faculty of Arts, Fayoum University, 63514, Egypt mhb00@fayoum.edu.eg

[2] School of Economics and Management, South China Agricultural University, Guangzhou, 510642, China aaa31@fayoum.edu.eg

[3] Department of Agricultural Economics, Faculty of Agriculture, Fayoum University, 63514, Egypt maa20@fayoum.edu.eg

* Correspondence: Tan tanyw@scau.edu.cn, Ali aaa31@fayoum.edu.eg

†These authors contributed equally to this work and should be considered co-first authors

Abstract

Environmental migration is a global phenomenon where increasing soil salinity leads to decreased productivity and income, subsequently increasing migration intention (MI) in the affected regions. This study investigates how agricultural land degradation (ALD), particularly soil salinity, reduces productivity and influences MIs in Egypt. Employing Protection Motivation Theory (PMT) as a framework, we analyzed primary data from 1,782 respondents using a multinomial logistic model. The empirical findings revealed that socioeconomic characteristics, agricultural engagement, and ALD significantly shape and determine MI. Furthermore, over 35.8% of respondents reported a decline in land productivity in the past decade due to environmental change, with 22.4% actively seeking relocation. Among those willing to migrate, the capital Cairo (40.2%) and international destinations (29.4%) were the most desired. Additionally, gender, age, and income adequacy were identified as crucial predictors in migration dynamics. The findings challenge conventional demographic assumptions by revealing a significant propensity to migrate among older populations and women, groups often considered less mobile. The primary motivations (employment and income) are directly exacerbated by ecological pressures like declining land productivity. Therefore, effective policy must address this nexus to reduce threat appraisal by implementing sustainable land and water management and investing in both environmental adaptation (e.g., soil restoration) and human capital (e.g., education, local economic opportunities) to mitigate displacement pressures and foster sustainable rural resilience.

Keywords: Soil Restoration; Environmental Stresses; Rural-Urban Migration; Protection Motivation Theory; Migration Stream; Rural-urban Displacement

1. Introduction

1.1 Background

Environmental stresses often cause socioeconomic vulnerability, leading to the erosion of household resources and encouraging migration as a survival strategy for many rural residents[1,2]. Migration has been considered a global challenge in recent years, where the interaction between a set of environmental changes and socioeconomic characteristics plays a role in migration decisions and moving vulnerable communities from one region to another, particularly in developing countries[3,4] . Climate change (CC) is projected to negatively affect from 50 million to one billion people from different regions in 2050, increasing the people in movement and migration, and putting pressure on the natural resources in many areas [1,5]. However, environmental migration has been considered a simple indicator of communities' failure to adopt CC; nevertheless, the migrants could support their local families left behind (in the origin areas) through remittances, which will be invested to enhance the infrastructure and improve the lives of their families. [6,7]. The pull and push drivers, causes, impacts, and types of migration have been presented in **Fig. 1**.

Currently, the global population redistribution is due to migration and socioeconomic changes, where some regions have become less habitable due to conflicts, climate change, water pollution or scarcity, agricultural land deterioration, and desertification, driving people to seek better lives elsewhere [2,8,9]. People in all countries and cultures strive for satisfaction, well-being, and a good quality of life elsewhere [10,11]. This pursuit of a good quality of life changes dynamically as it is influenced by external stimuli, such as peer interaction or social comparisons of the individual's status with others in similar circumstances [12]. Complex interactions between socioeconomic, political processes, and environmental factors could result in heterogeneous potential migration outcomes [13]. Livelihoods in the degraded regions are closely interconnected with migration, particularly where the people mostly depend on agricultural land [8]. Then, combating land degradation should be considered while designing and implementing the interventions to tackle regional or international migration.

In 2019, Egypt was the most prominent African country in terms of migrants, according to the number of people (~4 million citizens) living abroad as migrant labor in the Gulf countries, mainly in Saudi Arabia and the United Arab Emirates [14]. However, the number of people coming to Egypt from the surrounding Arab countries is increasing annually due to the current civil conflicts in these countries, which increases the pressure on food demand and food security in Egypt, as it is one of the region's highest food importers [15]. Several empirical studies across various regions have highlighted the link between land degradation and rural-urban migration [1,4,8]. In Africa, countries such as Ethiopia and Niger demonstrate that severe land degradation and recurring droughts compel rural populations to migrate to urban centers in search of better opportunities [16]. In India, studies indicate that

land degradation, exacerbated by monsoon variability and water scarcity, contributes to rural outmigration, particularly among smallholder farmers [17]. Additionally, in Mexico, deforestation and soil erosion in rural areas have been identified as significant drivers of migration to urban areas, including to the United States [18].

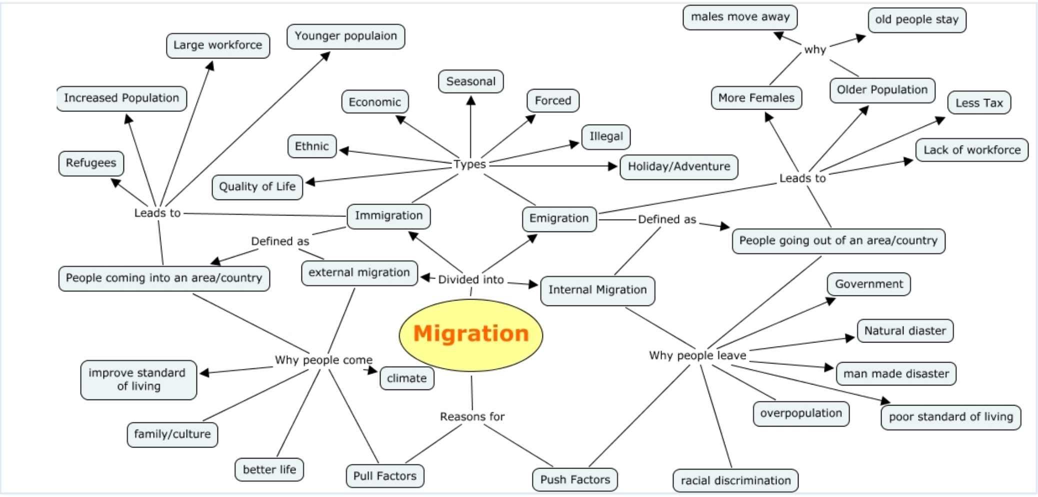

Figure 1 Migration pull and push drivers, causes, and their impacts.[1]
People migrate in search of better lives or as a result of natural disasters, climate change, and environmental degradation. The migration streams have numerous consequences for both the sender and receiver regions.

Furthermore, some case studies provide detailed insights into the mechanisms by which land degradation drives migration. In the Sahel Region, West Africa, land degradation, due to overgrazing and unsustainable farming practices, has led to significant rural-urban migration, and migrants often move to urban centers or coastal countries seeking employment [19]. While in Northern China, desertification in the Inner Mongolia region has resulted in large-scale rural displacement. Migrants typically move (temporarily or permanently) to the capital cities like Beijing and Tianjin, seeking better living conditions [20,21]. In Bangladesh, riverbank erosion and soil salinity in coastal areas have forced many rural inhabitants to migrate to urban slums in Dhaka, highlighting the link between environmental degradation and urban poverty [16,22].

The novelty of the current work could be highlighted through the following points: (1) our focus is to analyze how the interaction between socioeconomic characteristics and environmental change leads to migration intention in developing countries based on primary data. We apply a construct framework, combining the principles of the Protection Motivation Theory (PMT) and a multinomial logistic model, to predict the willingness to migrate under the potential ALD, which significantly contributes to increased migration streams in developing countries. PMT is a novel and creative theoretical lens for migration; we consider complementing PMT with the livelihoods vulnerability framework

[1] Migration concept map https://cmapspublic3.ihmc.us/rid=1295355828203_878224076_18749/Migration%20Concept%20Map.cmap

and/or Push-Pull models to enhance explanatory power. While existing research has explored the relationship between economic factors and migration [23], few studies have specifically analyzed how ALD influences migrants' decisions, particularly in terms of migration timing [1,3,4,8]. Most prior research has focused on general migration drivers [24,25], whereas our study sheds light on the more specific aspects of the role of climate change in determining when migrants will move from one region to another based on the degree of soil fertility and water availability in their origin rural area. (2) At the same time, we used the constructed framework to develop evidence-based recommendations about the specific push regions grounded on the degree of access to climate change mitigation and adaptation tools in rural areas in Egypt, which could contribute to future migration-reduction interventions. The result of the current study could best inform policymakers' and international donors' understanding of the environmental migration dynamics to design a strategy for addressing environmentally induced migration resulting from soil and land degradation in developing countries.

The structure of this paper is as follows: the next section is the literature review, and Section 3 is the methodology and data source. In section 4, we presented the results and discussion, and finally, we highlighted the main conclusion and policy implications.

1.2 Literature Review

Rural-urban migration is a global phenomenon that affects both developing and developed countries and has profound social, economic, and political implications [21,26]. Therefore, examining the factors affecting rural-urban migration, including their motives and barriers, is crucial for researchers, practitioners, and policymakers to understand the migration drivers and develop effective strategies and interventions for migration inhibition. In the following section, we highlight the comparisons and critiques of the existing literature discussing the impacts of (salinity, water shortage, and crop pattern change) as drivers leading to income decrease and consequently rural-urban and international migration.

A. Land degradation as a rural-urban migration driver

Land degradation is characterized by the decline in land quality and productivity due to various factors such as deforestation, overgrazing, and improper agricultural practices, which significantly impact rural citizens [4,8]. In many developing countries, ALD and the inability to sustain rural livelihoods have compelled individuals and families to seek opportunities in urban areas. According to a Global Assessment of Land Degradation and Improvement report, a quarter of the world's land area is now considered degraded. This degradation is caused by a combination of human activity and climate

change, resulting in the loss of fertile land, reduced agricultural productivity, and the displacement of millions of people (GALDI, 2018)[2]. Furthermore, soil salinity (accumulation of soluble salts in the soil) poses a severe threat to agricultural lands, particularly in arid and semi-arid regions like North Africa and Egypt. Salinity reduces soil fertility, thereby decreasing crop yields and agrarian income [27], this degradation compels farmers to abandon their land and seek better opportunities in urban areas. The recent study focused on sustainable land management practices in drylands, highlighting how salinity contributes to land degradation and subsequent migration [28]. Furthermore, soil salinity has a significant impact on agricultural productivity, where the salinity-induced yield losses drive rural populations to migrate to urban centres. This has appeared clearly in many countries in recent years, e.g., Egyptian youth are increasingly migrating from the environmentally affected areas and border villages to the city centers like Cairo and Alexandria, which increases the pressure on the infrastructure in the receiving cities [29,30].

Water access plays a significant role in global migration [31], while water deficit and scarcity are responsible for about 10% of the recent global migration (1 billion migrants) [32,33]. Although the impact of floods as a migration driver (floods have recently forced millions of people to leave their places), its negative consequences are lower than those of drought as a migration push factor [33]. More than 25% of the global population suffers from extreme water stress, especially in the developing world (more than 85% of people affected by rainfall variability live in low- or middle-income countries) [31]. As Egypt is located in the North Africa dry region, the agricultural water shortage in rural areas pushes populations towards urban centers, where more than 80% of the water is used for agriculture[34]. Water scarcity is another critical driver of rural-urban migration, where the over-extraction of groundwater, inefficient irrigation practices, and climate change exacerbate water shortages (which is common in the North African region), reducing sustainable water availability for agricultural activities [35]. As a result, crop yields decline, incomes fall, and rural inhabitants are forced to migrate to cities for better livelihoods [36]. This appears in the statistics, which show the high migration region, mostly from the dry and semi-dry regions [32].

Changes in crop patterns are often driven by environmental degradation and/or market drivers, which lead to decreased agricultural productivity and income, where the farmers may be forced to switch from high-value to low-value crops due to changing climatic conditions or soil degradation [2,37]. This shift reduces profitability and makes farming unsustainable (in many cases), prompting

[2] https://www.isric.org/projects/global-assessment-land-degradation-and-improvement-glada

rural residents to migrate to urban areas [35]. Some studies analyzed the impacts of the Green Revolution on migration, noting how changes in crop patterns due to environmental stressors affect rural incomes and migration patterns [38]. The same case is in the study area in Fayoum, which was the first city to cultivate rice in Egypt since 640 A.D. [39]. Rice was the main crop for farmers in Fayoum. Still, due to the water supply shortage in this region since the 2000s, the Egyptian government issued a new regulation in 2008 to prohibit rice cultivation in Fayoum, aiming to secure water sufficiency for other field crops in different regions. While *Morton* (2007) explores the impact of climate change on smallholder agriculture, highlighting how shifts in crop patterns driven by climate variability lead to decreased income and increased migration due to their vulnerability to adapt or mitigate these negative impacts [40].

B. Socioeconomic drivers and impacts of migration

Socioeconomic factors also play a crucial role in understanding migration decisions; in addition to education, better healthcare, and improvements in lifestyle, urban areas often offer better access to such services and opportunities, which attract people from rural areas who want to improve their quality of life [41,42]. In rural-urban migration, economic opportunity is considered the main driver, with migrants seeking better employment opportunities in urban centers, improved living conditions, higher wages, and more diverse jobs that typically do not exist in rural areas [41]. The lack of sustainable livelihoods and economic opportunities in rural areas, such as the lack of enough arable agricultural land and the lack of viable off-farm activities that generate additional income other than agriculture, can be an incentive for individuals to seek new, higher-income employment opportunities in urban areas [43]. Furthermore, the perceived economic prospects and greater availability of public services in urban areas compared to rural ones may provide a strong incentive for rural residents to move to urban areas [21,44]. According to the International Migration Report, migration can contribute positively to economic growth by filling labor shortages, particularly in sectors with high demand but low local supply of labor (e.g., agriculture & construction) [45]. Furthermore, migrants often send remittances back to their home countries, which can significantly contribute to poverty reduction and improve the living standards of families left behind (World Bank, 2021)[3].

Environmental changes, such as land degradation, climate change, and natural disasters, tend to encourage rural populations to migrate to urban areas [1,46]. Environmental stresses often cause soci-

---

[3] https://www.worldbank.org/en/topic/migrationremittancesdiasporaissues/brief/migration-remittances-data

oeconomic vulnerability that leads to the erosion of household resources by making traditional agricultural livelihoods unsustainable, thereby encouraging migration as a survival strategy for many rural residents [47]. As highlighted in the literature, the interaction between environmental changes and their socio-economic consequences is a complex process to understand the dynamics of migration [1], in which factors such as environmental changes, available adaptation strategies, and the accessibility of migration options play a crucial role in the decision-making process of the rural population [48].

On the other hand, increased migration can strain public services in urban areas such as healthcare and education, especially in areas with high migrant concentration, leading to budgetary challenges for governments (Migration Policy Institute, 2020)[4]. Moreover, cultural differences and perceived competition for resources can sometimes lead to social tensions and conflicts within communities and reduce the average wages due to the experience of the migrants and the high labor supply [33] Furthermore, the pressure on the infrastructure in the receiver cities will reduce their ability to withstand sudden shocks and also increase the losses during natural disasters such as floods and storms. At the same time, the food producers will decrease over time, which will increase the food import vouchers, especially in developing countries like Egypt. Also, it will increase the migration from rural to urban areas, which could reduce the economic growth in the rural areas over time.

C. Costs of rural-urban migration

Despite the potential benefits of migration, economic barriers such as moving costs, poor creditworthiness, and the risk of unemployment in urban areas can prevent migrants from moving [24,49]. When making a migration decision, potential migrants typically compare the costs associated with their decision with the benefits of migration. Migrants may face different costs, such as travel costs to the destination, subsistence costs when looking for work and housing, and any opportunity costs for activities that remain in the area of origin [25,50,51]. In addition, migrants face other costs due to differences in religion, culture, language, and ethnicity, which together contribute to shaping the migration decision [52]. For this reason, potential migrants prefer to choose a destination with a similar linguistic or ethnic background [51]. On the other hand, other migrants living in communities with different ethnic backgrounds may be able to integrate better than those living in neighbourhoods with few ethnic groups [53]. This is particularly evident for Arab and African people who have migrated to EU countries over the past few decades.

[4] https://www.migrationpolicy.org/

Rural-urban migration often leads to a reduction in the agricultural workforce in rural areas, impacting farm productivity and food security [54]. In urban areas, migrants may compete with locals for jobs, potentially driving down wages in specific sectors and contributing to higher unemployment rates among low-skilled native workers [54]. Furthermore, rapid urbanization due to migration strains urban infrastructure such as housing, transportation, water, and sanitation services, leading to inadequate living conditions in some cities. Moreover, increased urbanization and migration contribute to environmental degradation, including air and water pollution, deforestation, and loss of biodiversity [46,55]. Migration also affects one's physical and mental well-being. For instance, a recent study reveals that younger migrants from rural to urban areas of China report worsening health, which they attribute to shifts in social trust and emotional states [56]. In addition, older parents of migrants who settled in rural areas are more likely to experience health problems [57]. Migration can also result in social disintegration within rural communities as families are separated, leading to breakdowns in traditional support systems [58].

1.3 Theoretical Framework

The conceptualization of migration studies has been historically grounded in *Ravenstein's* 'Laws of Migration' (1885)[59], which first identified systematic patterns of migration flows, and later expanded by *Everett Lee's* Push-Pull Theory (1966)[60], which emphasized the interaction of push factors in origin areas and pull factors in destination areas. These classical theories provide the intellectual foundation upon which more recent approaches, including the Protection Motivation Theory (PMT), are built to explain environmentally induced migration, as described below.

The migration theories investigate the social change processes that are interlinked with human mobility, so many theories and approaches have been used to understand the root causes, motivations, and gains of migration. For example, De Haas has investigated the aspirations and capabilities of migration within given sets of perceived geographical opportunity structures and differentiates between human mobility's instrumental (means-to-an-end) and intrinsic (directly wellbeing-affecting) dimensions [61,62]. This yields a vision in which moving and staying are seen as complementary manifestations of migratory agency and in which human mobility is defined as people's capability to choose where to live, including the option to stay, rather than as the act of moving or migrating itself [62]. Furthermore, the labor policies in the destination countries play a significant role in facilitating the immigration of highly skilled labor in risky environmental areas where land degradation and negative impacts of climate change push the migrants to move from these areas to another safe one. At the same time, rural citizens may move to nearby cities or migrate to major capital cities, and some may even

immigrate to seek opportunities in another country. Climate change and natural disasters tend to encourage rural populations to migrate [1,46].

This study adopted the Protection Motivation Theory (PMT) to interpret the impact of environmental changes and stresses, such as land degradation, as a migration driver. The PMT was developed by Rogers in 1975 and modified in 1983 as a framework for analyzing how individuals react to perceived threats [63,64]. PMT initially focused on health-related behaviours, but it can also be modified to describe migration decisions, especially in situations where individuals encounter environmental, economic, or social challenges.

PMT is a novel and creative theoretical lens for migration; we consider complementing PMT with the livelihoods vulnerability framework and/or Push-Pull models to enhance explanatory power. The following outlines how the primary elements of PMT—threat appraisal and coping appraisal—can clarify migration behavior, **as shown in Fig. 2**:

1. Threat Appraisal (Perceived Severity & Vulnerability)

Migration often happens once individuals perceive a substantial threat to their safety, well-being, or livelihood. For example, the farmers who are facing ALD may perceive yield reduction as a severe threat and believe they will face socioeconomic vulnerability if they stay in the origin regions, which encourages many rural residents to migrate [39]. Furthermore, the interaction between environmental changes and their socioeconomic consequences is a complex process [1], in which factors such as environmental changes, available adaptation strategies, and the accessibility of migration options play a crucial role in the decision-making process of the rural population [47], as shown in **Fig. 2**.

2. Coping Appraisal (Response Efficacy & Self-Efficacy)

Although the threat is perceived to be severe, the migration decision depends on the individual's beliefs about how to respond effectively. For example, the farmers who are facing ALD may feel capable of migration if relocation to another place offers better income and living conditions, and they have the legal pathways and resources (savings and relatives abroad), while poor growers may not.

3. Fear vs. Adaptive Response

Despite the high threat to the poor farmers in the region with ALD, they may stay if they have insufficient resources to relocate (low coping appraisal).

4. External & Internal Rewards (Deterrents to Action)

PMT also considers disincentives that may restrict, delay, or reduce the willingness to migrate under threat, including government aid, social support, and attachment to one's homeland.

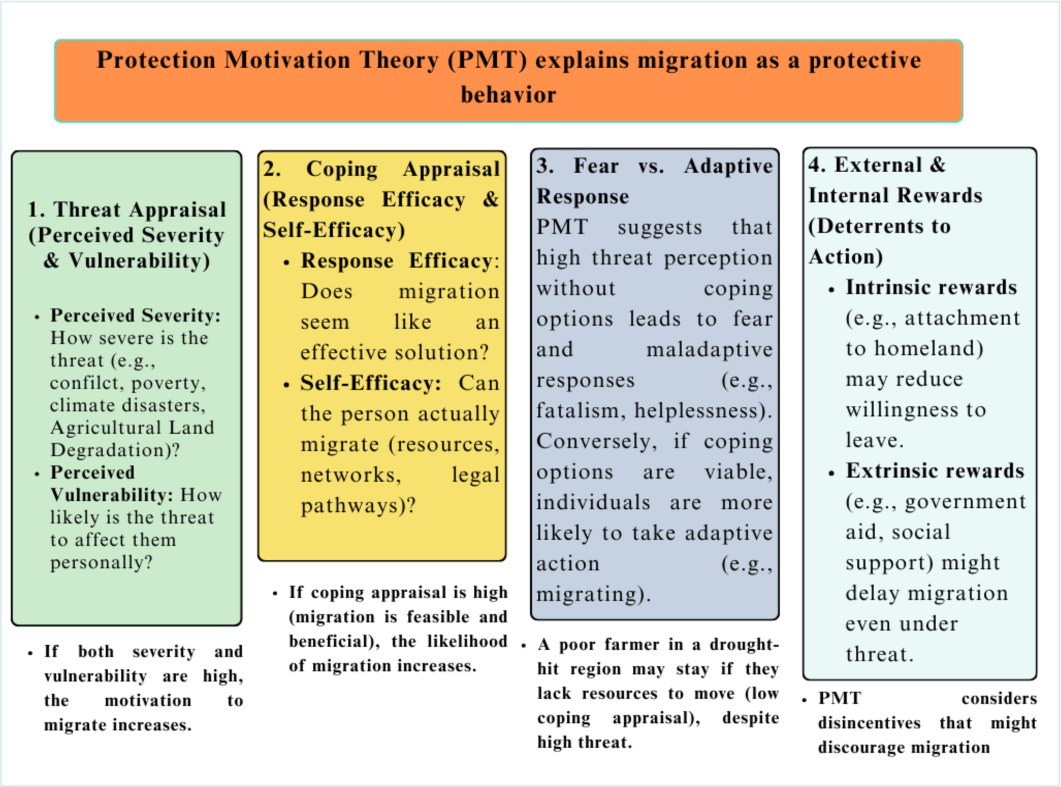

Figure 2 Protection Motivation Theory (PMT) explains migration as a protective behavior.

We summarize the potential consequences of environmental change, particularly the ALD, to highlight the interaction between socioeconomic and environmental stress as a driver of migration. As illustrated in **Figure 3,** the consequences of agricultural land degradation (ALD) on migration intention can be interpreted through the Protection Motivation Theory (PMT). Specifically, ALD increases threat appraisal by raising farmers' perceived vulnerability (e.g., declining productivity, loss of income) and severity (e.g., livelihood insecurity). At the same time, migration as a coping response depends on coping appraisal, where individuals evaluate their ability to relocate successfully given available resources, skills, and networks. The interaction of these processes, heightened threat appraisal alongside varying levels of coping appraisal, shapes the willingness to migrate depicted in the figure. Thus, **Figure 3** integrates PMT dimensions with empirical evidence of ALD-induced migration intentions.

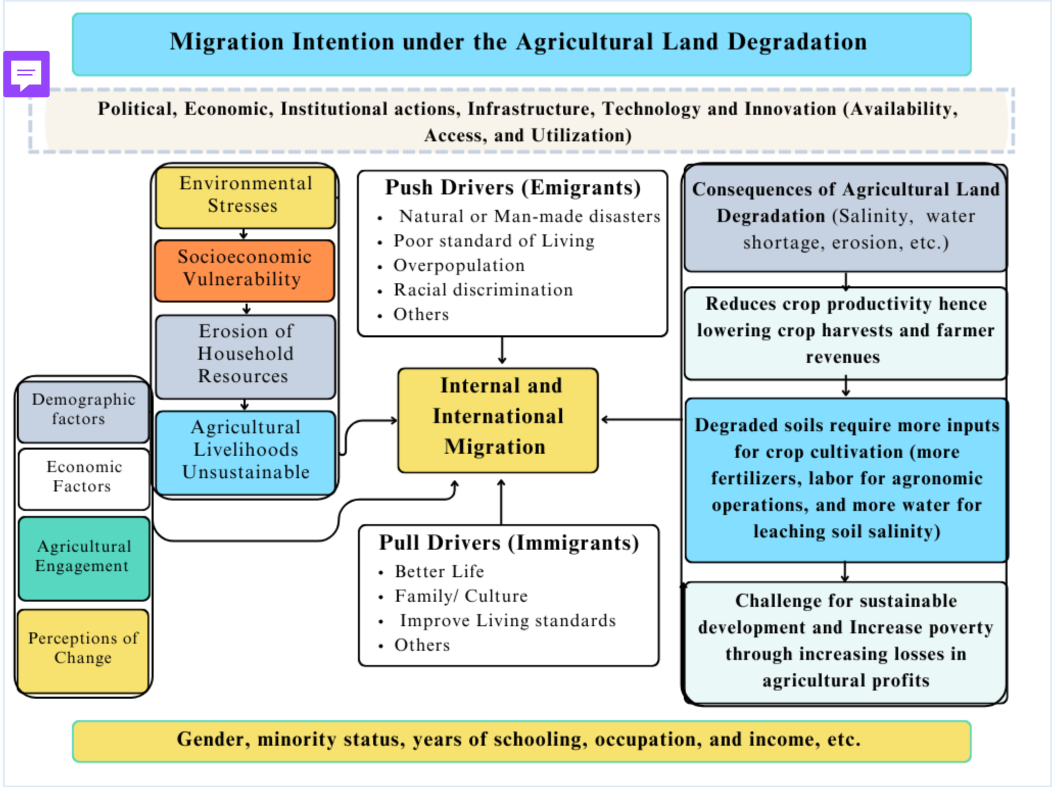

Figure 3 Migration drivers: the push-pull factors of migration under agricultural land degradation, besides the potential consequences of environmental change.

2. Materials and Methods

2.1 Study Area and Sampling Strategy

The current study adopted a multi-stage approach (cluster sampling followed by systematic random household selection within each cluster), ensuring that the sample adequately represents the demographic and agricultural diversity of the selected community, making the findings generalizable to the broader rural population. We first chose Fayoum Governorate, **Figure 4**, Egypt, which is often described as a microcosm of Egypt due to its geographical, agricultural, and demographic characteristics. Fayoum, as an oasis in the desert, suffers from water shortage due to its location on the downstream branch of the Nile River (*Bahr Youssef* canal), the only water source (for agriculture and drinking), which resembles the role of the Nile River for Egypt. Its waters discharge into *Lake Qarun* and *Wadi El-Rayan* areas, which resemble Egypt’s Mediterranean outlets. Fayoum is one of the leading exporters of both internal and international migrants in Egypt. Many of its villages are well known as international migration exporters, such as *Tatoun city* (to Italy*) and Shidmu city* (to France*)*. The governorate is predominantly rural, with about 26.4% of its population living below the poverty line in 2019. The average percentage of the rural population in Egypt was 57.6% in 2017; this percentage rose

to 76.7% in Fayoum Governorate, and within Fayoum Governorate itself, the percentage of rural people rose to more than 90.4% among the population of *Itsa* district (CAPMAS, 2017).

Fayoum is considered a representative for Egypt in the water shortage issue (downstream of the Nile River Basin), where the water shortage in upstream countries will negatively affect Egypt as a country located in a dry region and suffering from groundwater limitation, doubling the problem, especially for agricultural water. This water shortage resulted in many issues, including high soil salinity, decreased agricultural productivity and income, and changes in the crop pattern in the last decades (to mitigate the negative impacts of this water shortage). Previously (before 2008), rice was the main crop in Fayoum (*Itsa* districts). However, after the prohibition of the cultivation of rice, the soil salinity increased in this region, and many farmers left the land or suffered from low income from agriculture as a primary income source.

For the geographical distribution of the selected sample, we chose *Itsa* district as the study area, as the last district in the *Yousef canal* branch, and it is the largest district in terms of both area and population (708,780 people, about 20% of the governorate's population in 2017) (CAPMAS, 2017)[5]. In total, 1,782 households were investigated. One adult respondent was randomly selected from each sampled household. The overall sample size was also cross-validated using Cochran's formula (1977) at a 95% confidence level and 5% margin of error [66], and adjusted for potential non-responses. To ensure representativeness, a cluster sampling strategy was adopted. The twelve local villages of *Itsa* district were divided into four geographically contiguous clusters, each forming a sub-region within the district, as shown in **Table A.1**. Sample sizes in each cluster were calculated using Thompson's formula and cross-checked with Krejcie and Morgan's (1970) sampling tables to meet minimum requirements [65].

5 **CAPMAS** (Central Agency for Public Mobilization and Statistics) www.capmas.gov.eg

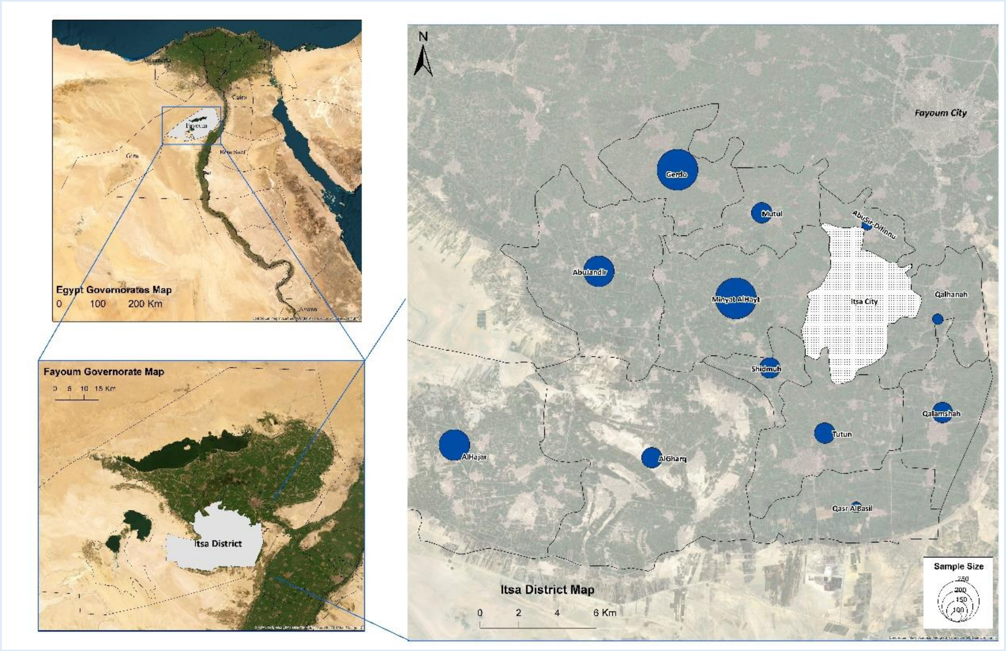

Figure 4 Study area map and distribution of the selected respondents
Sources: Esri, Fayoum Governorate, and Itsa district maps. (December 25, 2024).
https://www.arcgis.com/home/item.html?id=09c9d0af95d24bc7a1b6d8859b59e428

A structured questionnaire was developed and pretested to ensure its accuracy for achieving the study objectives, and we interviewed the respondents face-to-face in 2023, and they self-reported the answers to the proposed questions.

2.2 Data Analysis and Empirical Approach

Many econometric models have been employed to understand the impacts of socioeconomic demographics on the likelihood of changing the place of residence and moving from rural to urban areas or immigrating abroad. In this study, the Multinomial Logistic Regression (MLR) model was employed to analyze the impact of socioeconomic demographic characteristics on the ability and willingness to migrate.

[illegible]gistic regression (LR) is used when the dependent variable is nominal (or categorical). For Binary logistic regression (two dependent variables) and multinomial logistic regression, there are more than two [67]. In LR, a logistic transformation of the odds (referred to as logit) serves as the dependent variable [67,68]:

$$\log(odds) = \text{logit}\ (\text{P}) = \ln\left(\frac{P}{1-P}\right) = a + \beta_1 x_1 + \beta_2 x_2 + \beta_3 x_3 \ldots\ \beta_n x_n \qquad (1)$$

Or

$$P = \frac{exp(a+\beta_1 x_1+\beta_2 x_2+\beta_3 x_3 \ldots\ldots)}{1+exp(a+\beta_1 x_1+\beta_2 x_2+\beta_3 x_3 \ldots\ldots)}$$

Where:

p = the probability that a case is in a particular category,

exp = the exponential (approx. 2.72),

a = the constant of the equation and,

b = the coefficient of the predictor or independent variables.

Logits or Log Odds

Odds value ranges from zero to infinity and tells us “how much more likely it is that an observation is a member of the target group rather than a member of the other group” [53].

$$\text{Odds} = p/(1-p)$$

The odds ratio (OR), estimates the change in the odds of membership in the target gro[illegible] for a one-unit increase in the predictor [67]. It is calculated by using the regression coefficient of the predictor as the exponent or exp.

In our case, the Y (dependent variable) is the desire to change the place of residence and leave the village (Migrate), where "0" is "No, I don’t want", "1" is "Maybe I want", and "2" is "Yes, I want". The explanatory variables include demographic factors, economic factors, and agricultural factors, as presented in **Table A2**. For the perceptions of change based on available data that include (age, family size, agriculture share of income, gender, marital status, educational status, monthly income, income adequacy, working in the agricultural sector, ownership of agricultural land, changes in land ownership during the last 10 years, changes in the farming land during the previous 10 years). The rice crop cultivation, beet crop cultivation, changes in land productivity, living conditions of the farmer now compared to 10 years ago, and current village conditions compared to 10 years ago) have been considered.

The reference category for gender is "Males", marital Status is "Married", educational Status is "high-school", and monthly income is "from 2000 to 4000 LE.[6]", income adequacy is "Somewhat enough", working in the agricultural sector is "Yes", ownership of agricultural land is "Yes", Changes in land ownership during the last 10 years is "Area has not changed", Changes in the farming land

---

[6] 1 $USA = 30.743 LE (Egyptian pound as an average 2023) based on the Central Bank of Egypt data. https://www.cbe.org.eg/en

(cultivated area) during the last 10 years is "Area has not changed", Rice crop cultivation is "No cultivation", Beet crop cultivation is "No cultivation", Changes in land productivity is "Now better", Living conditions of the farmer now compared to 10 years ago "Now better", and Village conditions now compared to 10 years ago is "Now better".

Finally, we used the weighted percentage to rank the motivations and barriers of migration under the ALD as follows: Weighted percentage % = $\frac{\text{Total marks}}{N*3}$ *100, where N represents the sample size. We used the same model to assess the impact of socioeconomic characteristics of respondents on motivation and barriers to migration intention through a composite variable.

3. Results and discussion

The following analysis is guided by the Protection Motivation Theory (PMT), which frames migration intention as an outcome of both threat appraisal and coping appraisal. In this study, threat appraisal is reflected in how farmers perceive the severity and vulnerability associated with agricultural land degradation, while coping appraisal is captured by their resources, education, and access to alternative livelihood strategies. Accordingly, each set of results is interpreted in relation to these theoretical dimensions to ensure consistency between the framework and empirical findings. Environmental changes, such as land degradation, climate change, and natural disasters, tend to encourage rural populations to migrate to urban areas [1,46]. In the following section, we will discuss the main results, including the interaction between socioeconomic factors and environmental stress as drivers for migration.

3.1. Descriptive statistics

The respondents' socioeconomic characteristics have been presented in **Tables A2 and A3,** where more than two-thirds of the respondents (83%) were males and married (68.9%), and most of them were educated (80%). However, more than 40% of them don’t own agricultural land, and around one-quarter (26%) of them depend on agriculture as a primary income source. The findings from the descriptive statistics reveal significant trends regarding land ownership and farming areas. Nearly half of the respondents (46.3%) reported no change in land ownership over the past decade, while 53.3% indicated stability in the area they currently cultivate. This reflects limited land market activity and the role of inheritance in maintaining land ownership structures. This is consistent with Deininger and Jin, who noted that land tenure systems in developing countries often restrict land redistribution, leading to long-term stability in ownership [69].

Regarding crop structure, rice remains a dominant crop, with 60.4% of respondents cultivating it only within the past 10 years, highlighting its importance in local agricultural systems in the investigated area. This finding highlights the difficulty of changing the cropping system, which aligns with Elmpghazy and Elshenawy, who reported that rice is a staple in rural Egypt due to its profitability and the long farmers' experiences, and they consider it a main crop in most of the northern Egyptian governorates [39]. For sugar beet cultivation, the distribution is more balanced, with 36.9% cultivating beet both currently and a decade ago, suggesting the role of crop rotation and adaptation to market demands [3]**.**

A concerning trend is the decline in land productivity, as reported by 35.8% of respondents. This decrease may be attributed to factors such as soil degradation, water shortages, and climate change. Mahmoud (2019) found similar patterns in Egypt, where environmental stressors have contributed to declining agricultural productivity and, consequently, lower farm incomes and increased migration rates [34]. Economic challenges further exacerbate this, as the study shows that 43.4% of respondents earn between 2000 and 4000 EGP (less than $80) per month. This range limits financial stability and motivates people to migrate in search of a better life for their families. Moreover, only 26.6% of respondents considered their income sufficient, reinforcing findings from Radwan et al. (2022), who highlighted persistent rural poverty in Egypt due to limited off-farm employment opportunities [70].

The study also captures perceptions of agricultural production and living conditions. A significant proportion of respondents (44.2%) believe that agricultural production has degraded over the past decade. Zohry (2005) attributes this decline to increased production costs, limited access to quality inputs, and environmental challenges. Similarly, more than half (53.4%) of respondents specified that living conditions have worsened in the last ten years due to the higher living costs and a decrease in the sufficiency of income [30]. This trend is supported by Abdelwahed et al. (2020), who found that rural communities in Egypt have struggled due to inflation and economic reforms that have not equally benefited all social classes [29]. This highlights the significant role of social protection and women's empowerment in reducing the streams of migration from the vulnerable areas.

Migration trends show that 45.2% of respondents do not wish to leave their community, while 22.4% actively seek relocation, which will be discussed in the motivation and barriers section later. Among those willing to migrate, Greater Cairo (40.2%) and destinations outside Egypt (29.4%) were the most desired. These patterns reflect broader economic migration trends, where rural populations move to urban centers or abroad in search of better opportunities for their families [30]**.** The primary motivations for migration were access to suitable job opportunities (70.1%) and higher wages (66.8%),

which is consistent with findings from Abdelwahed et al. (2020), who emphasized that economic factors are the strongest drivers of rural-to-urban migration in Egypt [29]. This highlights the importance of empowering farmers to utilize advanced technology, thereby enhancing their income and reducing migration streams from vulnerable rural areas to the main cities or abroad.

Despite the desire to migrate, several barriers exist, with the most cited obstacles being the difficulty of moving (54.1%) and the high cost of relocation (52.7%). Our findings align with those of Abdelwahed et al. (2020), who found that limited financial resources and the lack of affordable housing often deter rural residents from moving. Similarly, inadequate services in rural areas also influence migration decisions, with 48.5% of respondents dissatisfied with both educational and healthcare services [29]. These findings align with Zohry (2005), who reported that rural Egypt faces severe challenges in access to quality education and healthcare due to underinvestment in infrastructure [30]. This underscores the essential need for equitable economic development across the regions in reducing the pressure of migration, and gives priority to the vulnerable areas for public investment in education, health, and infrastructure.

3.2. Determinants of migration intention

The results of the migration determinants that play a significant role in motivating or inhibiting people to migrate from one region to another have been presented in **Tables 1 and 2**. The interrelationship between these variables and willingness to relocate, which is represented as (yes, I will relocate or maybe). This includes the demographic, economic, and farming systems of the selected respondents, presented as follows;

3.2.1 Model Fitting and Pseudo R-Square

The multinomial logistic regression model was evaluated using several key metrics, beginning with the model fitting information. The final model had a significantly lower -2 Log Likelihood value (3272.3) compared to the intercept-only model (3772.3), indicating a better fit of the whole model. The Chi-Square statistic for the model was 499.98 with 80 degrees of freedom, which is statistically significant ($p < 0.001$). This suggests that the model as a whole is a good fit for the data.

Pseudo R-square values, including Cox and Snell (0.245), Nagelkerke (0.278), and McFadden (0.132), further indicate the model's explanatory power. While these values do not have the same interpretation as R-squared in linear regression, they do provide a measure of the proportion of variance explained by the model. In this case, the Nagelkerke R-square value of 0.278 suggests that the model explains about 27.8% of the variance in the dependent variable, which is moderately strong for social science data.

3.2.2. Demographic Factors

Age: The results show that age is a significant predictor ($p < 0.001$) across all categories, with an odds ratio (Exp(B)) less than 1 (0.944) for "maybe" and 0.951 for "yes". This suggests that with increasing age, the respondent's willingness to migrate and leave the village will decrease. This is consistent with previous studies' findings that younger individuals were more inclined to migrate compared with older populations under the same risk sources. A recent study confirmed that young Egyptian people (18-29 years) are more motivated to migrate based on their age, marital status, and employment status [29]. Although the employment status was insignificant as a migration driver after the Arab Spring of 2011, compared with before it (2008), the remaining factors still significantly drive internal and international migration [29]. For instance, especially in rural areas, older adults are generally more inclined to stay in their current residences due to established social networks and greater attachment to their communities [29]. Furthermore, the youth could be more aware of the negative impacts of climate change, which increases their willingness to move from the most affected regions (rural) and sectors (agriculture) to other service sectors in the cities [52]. Based on the PMT assumptions, our results confirm that the youth are more motivated to relocate due to their higher perceived severity and vulnerability, which increases their motivation to relocate, and lower restrictions to delay relocation, compared to elderly people with families. However, the willingness to migrate will be lower under the government aid, social support, and attachment to one's homeland, which should be considered for future intervention.

Table 1: Overall effect of the main variables on the migration intention

Likelihood Ratio Tests			
Effect	Model Fitting Criteria	Likelihood Ratio Tests	
	-2 Log Likelihood of Reduced Model	Chi-Square	df
Intercept	3272.342^{a}		
Age	3389.866	117.523***	2
Family Size	3278.914	6.572**	2
Agricultural share of total income	3276.911	4.569	2
Gender	3290.362	18.020***	2
Marital Status	3277.869	5.527	6
Educational Status	3294.111	21.769**	10
Working in the agricultural sector	3277.028	4.686*	2
Agricultural land ownership	3273.606	1.264	2

Changes in Agricultural land ownership in last 10 years	3274.118	1.776	6
Changes in total cultivated area in the past 10 years	3276.300	3.958	6
Monthly Income	3315.200	42.858***	6
Income adequacy	3289.551	17.209**	4
Changes in land productivity	3281.266	8.924*	4
Living conditions of the farmer now (compared to 10 years ago)	3280.092	7.750	4
Changes of village conditions now compared to 10 years ago	3275.188	2.846	4

Model Fitting Information

Model	Model Fitting Criteria	Likelihood Ratio Tests	
	-2 Log Likelihood	Chi-Square	df
Intercept only	3772.331		
Final	3272.342	499.989**	80

Pseudo R-Square

Cox and Snell	0.245
Nagelkerke	0.278
McFadden	0.132

The chi-square statistic is the difference in -2 log-likelihoods between the final model and a reduced model. The reduced model is formed by omitting an effect from the final model. The null hypothesis is that all parameters of that effect are 0.

a. This reduced model is equivalent to the final model because omitting the effect does not increase the degrees of freedom.

*** Values are significant at P = 0.01, ** Values are significant at P = 0.05, * Values are significant at P = 0.1

Family Size also emerged as a significant predictor for the "maybe" category (p = 0.016, Exp(B) = 1.078), suggesting that larger families are more likely to consider moving as a whole or send some family members to capital cities or to abroad as well to secure more income for the family members, which consistency with Abdelwahed et al. (2020) [29]. This could be because larger families might seek better living conditions or more opportunities, which aligns with previous research highlighting the impact of family size on migration decisions, such as the study by Mulder and Cooke (2009), who observed that households with more members may seek relocation to improve living conditions or access better services [71]. Due to the nature of social networks in Fayoum as a tribal society, most families prefer to have more children, especially males who will immigrate to EU countries later (it is considered a social superiority in this society). Larger families might also relocate to urban areas to

access better educational opportunities for their children, a common trend observed in rural-to-urban migration studies. At the same time, changes have occurred in the crop pattern in Fayoum, which could be another reason. The previous crop rotation, which included rice and cotton, was able to observe a labour supply surplus due to its need for intensive human labour in farm operations and harvesting. The fragmentation of agricultural land (under the limitation of reclaiming new lands) has another impact on reducing per capita land share and decreasing the rural income due to the land and water limitations in the study area, where the literature revealed that the fragmentation of agricultural land decreases the profitability of farms in the rural areas and leads to migration [72].

Gender significantly affects the decision to move, with males being less likely to want to change residence ($p < 0.001$, Exp (B) = 0.521 for "maybe" and 0.495 for "yes"). This gender difference is consistent with the literature that suggests men often have stronger ties to land and agriculture, making them less inclined to migrate. This supports findings from studies like those by Mulder and Cooke (2009) [71], who noted that women, particularly in patriarchal societies, might seek to relocate for better employment opportunities and autonomy. This pattern reflects broader gender dynamics where women might perceive urban areas as offering more freedom and opportunities. However, given the nature of Egyptian social norms, it would be expected that women have a low willingness to migrate, but due to globalization and access to ICT, the new generations of women are more willing to migrate compared to men [58]. This could go back to the land ownership in the rural areas in Egypt, mostly registered for male householders rather than women, which increased the women's desire for more empowerment that could be more available in the urban regions. Furthermore, the gender differences observed are significant, highlighting the ongoing gender disparities in rural areas where men might have more opportunities locally. At the same time, women perceive urban areas as avenues for greater autonomy and employment. This is consistent with the literature findings that revealed that rural women often migrate to escape restrictive social norms and pursue independent livelihoods [73]. On the other hand, the women who are looking for more women's empowerment prefer to leave the village to be able to find good opportunities for better education, employment, and a mirage in the capital cities [74].

Educational Status: Education level was significant for both categories (yes, maybe), particularly for higher education levels ($p < 0.001$ for some categories). Individuals with higher **education** were more likely to consider moving (Exp(B) = 1.760 for "maybe" and 2.089 for "yes"). This finding supports previous studies that have demonstrated that education increases awareness of opportunities outside rural areas and thus increases the likelihood of migration [74,75]. For example, it is noted that

education significantly predicts migration intentions, particularly among younger adults seeking professional development [75]. The recent literature revealed that education has a significant impact on motivating young people to migrate; at the same time, those people who have already migrated will invest more to support their families and receive a good education [33,76]. A recent study confirmed that young Egyptian laborers have more opportunities in Arab countries when they have a higher education degree than uneducated people [29]. In the same vein, highly skilled and educated individuals are more likely to immigrate, particularly if they have already relocated from their birth village (internally migrated) to pursue their education in the city or abroad.

Our findings indicate that demographic and social factors, particularly age, family size, gender, and education, play a crucial role in determining the likelihood of wanting to relocate. Based on the PMT assumptions, our results suggest that older individuals are more inclined to remain in the village, possibly due to stronger ties to the community or a reduced propensity for change. In contrast, higher educational attainment and greater income levels increase the likelihood of wanting to leave, possibly reflecting aspirations for better opportunities elsewhere [46,77]. These results align with the broader literature on migration and residence decisions, particularly in rural contexts [77]. The significant role of the combination (age, family size, gender, and education) reflects well-documented trends in the migration literature. For instance, younger individuals and those with higher education levels are typically more mobile, seeking better opportunities in urban areas [29,74]. Our results confirm the PMT assumptions, indicating that higher perceived severity and vulnerability increase motivation to relocate, while lower restrictions to delay relocation are observed compared to elderly people with families. Nevertheless, the inclination to migrate is likely to decrease due to government assistance, social support, and a strong connection to one's homeland, all of which should be taken into account for future interventions.

3.2.3. Economic Factors

Income and Income Adequacy: Both monthly income and perceived income adequacy were significant predictors. Interestingly, lower income was associated with a higher likelihood of wanting to leave the village, while perceptions of income adequacy showed a complex relationship, which is consistent with [78]. Lower income groups were more likely to want to move ($p < 0.001$, Exp(B) = 0.573 for "maybe" and Exp(B) = 1.735 for "yes"). The significant role of income in migration decisions aligns with economic theories of migration and PMT assumptions, such as those proposed by [79],

which posit that individuals are more likely to move when they perceive better economic opportunities elsewhere. However, the complex relationship with income adequacy reflects the findings of studies like that by [78], which suggests that subjective perceptions of economic well-being can be as crucial as actual income in migration decisions. Furthermore, the political economy of EU-Egypt migration has been discussed to stop the irregular flows to the EU countries through the Mediterranean Sea for the people who want to secure high-wage jobs that aren't available in their domestic (Egypt) labour market [23,80]. These studies confirmed that the Egyptian labor market can export skilled labor to the EU or other Arab countries by establishing training centers to enhance the skills of those people willing to migrate.

3.2.4. Agricultural System Factors

Agricultural Engagement: Working in the agricultural sector was negatively associated with the desire to move from villages (for the land owners), though the association was not always significant ($p = 0.053$). This could indicate a strong attachment to agricultural livelihoods among those employed in this sector. These findings are consistent with the study of Gray et al., who found that individuals engaged in agriculture were less likely to migrate, as they were more attached to their land and agricultural activities [9]. However, we observed that the young rural people in the selected area were mainly willing to migrate due to the negative impacts of climate change and environmental stress on the agricultural sector, which puts this sector at high risk. Another study has discussed how households with strong agricultural livelihoods often exhibit lower migration rates, particularly in contexts where agriculture remains a viable livelihood strategy [81]. Most of the farmers in the selected area depend on agricultural production as their primary income source, where the shortage of water increases the salinity of the soil, decreasing the productivity and income of those farmers and forcing them to migrate (in case they don't own land or their ownership is a small area).

Changes in land productivity or farming systems due to climate change and other socioeconomic factors push many people to migrate, prompting stakeholders to seek different employment opportunities in urban areas. This encourages rural-urban migration; this finding is consistent with the recent literature [41,52]. The recent literature revealed that rural people in Africa will face a decline in agricultural production due to the negative impacts of climate change and land degradation, and they will be the most affected and exposed to migration [82], especially in vulnerable areas like the study area (Fayoum). Unfortunately, most of the young people in the selected area are either willing to migrate to the EU countries through illegal migration or move (temporarily or permanently) to find work

in capital cities like Cairo or Alexandria, which decreases the labour supply during the seasonal demand for agricultural labour in this area. Salinity reduces soil fertility in the selected area, thereby lowering crop yields and forcing the farmers to change the cropping pattern, which finally decreases the agricultural income. This degradation compels farmers to abandon their land and seek better opportunities in urban areas [27]. Our findings align with recent research, which found that desertification in the Inner Mongolia (Northern China) region has resulted in large-scale rural displacement[20]. Also, in Bangladesh, riverbank erosion and soil salinity in coastal areas have forced many rural inhabitants to migrate to urban slums in Dhaka, highlighting the link between environmental degradation and urban poverty [16,22]. This highlights that increasing soil salinity leads to decreased productivity and income, and subsequently an increase in migration willingness in the future. The intervention should focus more on the technologies and farming practices that enhance the soil fertility under the water shortage and soil salinity to reduce the stream of migration in future interventions.

3.2.5. Perceptions of Change

Perception of Living Conditions: Individuals who believed that living conditions for farmers had improved over the last decade were less likely to consider moving ($p = 0.033$). This highlights the importance of perceived improvements in quality of life in retaining rural populations. This finding supports previous studies that have demonstrated how perceived improvements in local living conditions, particularly in rural areas, can reduce the likelihood of migration by enhancing satisfaction with local opportunities and quality of life [83]. Migration decision depends on the interaction of a set of variables, including migrants' socioeconomic characteristics and environmental change, that should be considered during the design of the intervention to tackle migration. The real example, in this case, is the national initiative in China (rural revitalization), which aims to support rural communities to decrease rural-urban migration and secure agrofood products for local self-sufficiency [83].

Although some variables included in the current study have been statistically insignificant e.g, marital status and ownership of agricultural land, these factors are still influential in understanding and explaining the migration intention and decision, which have been confirmed in the literature [25,50,51]. Future studies could therefore investigate the interaction between these sets of variables in different regions to understand migration motivations better.

Table 2 The willingness of the respondents to migrate from their current place (results of the multinomial regression models)

Do you want to change your place of residence and leave the village? (Maybe or yes)	(1) Maybe		(2) Yes	
	Std. Error	Exp(B)	Std. Error	Exp(B)
Intercept	0.366		0.420	
Age	0.006	0.944**	0.007	0.951***
Family Size	0.032	1.078*	0.035	1.068**
Agriculture share of income	0.218	0..662	0.006	1.018
[Gender=1] Females	0.185	0.521***	0.194	0.495***
[Educational Status=1] Illiterate	0.191	1.038	0.219	0.753
[Educational Status=2] Reads and writes	0.186	1.161	0.219	0.918
[Educational Status=3] Basic education	0.280	1.072	0.331	0.900
[Educational Status=4] University and post-university	0.200	1.760**	0.213	2.089**
[Educational Status=5] Institute (above intermediate)	0.261	1.681*	0.314	1.240
[Do you work in the agricultural sector?=0] No	0.166	0.725*		
[Income (monthly)=1] (less than 2000)	0.173	0.573***	0.173	1.735**
[Income (monthly)=2] (4000- 6000)	0.156	0.826	0.193	0.783
[Income (monthly)=3] (more than 6000)	0.206	0.828	0.231	1.013
[Income adequacy=1] Not enough	0.144	1.014		
[Income adequacy=2] Enough	0.155	0.597***		
Living conditions of the farmer now compared to 10 years ago= (Now worse)	0.171	1.439**		
Living conditions of the farmer now compared to 10 years ago= (As it was 10 years ago)	0.200	1.083		
Changes in land productivity =2] As it was 10 years ago			0.226	0.559**

*** Values are significant at P = 0.01, ** Values are significant at P = 0.05, * Values are significant at P = 0.1

3.3. Motivations and Barriers of Migration Intention

3.3.1 Motivations of Migration

The primary driver of migration is the availability of suitable job opportunities in another governorate, with 70.1% of respondents agreeing on this factor, ranking it first with a weighted percentage of 84.3%, as shown in **Table 3**. This result aligns with Radwan et al. (2022), who found that the lack

of job opportunities in rural areas significantly pushes internal migration in Egypt [84]. Similarly, the pursuit of higher wages or salary increases ranked second (66.8%) with a weighted percentage of 83.0%, confirming that economic incentives play a crucial role in migration decisions [29]. The possibility of starting a project with a higher income in destination areas ranked third (62.4%) with a weighted percentage of 81.3%, reflecting the aspiration for better income-generating activities. This finding aligns with Abdelwahed et al. (2020), who reported that rural populations often seek self-employment opportunities as an alternative to low agricultural incomes [29]. Furthermore, the inadequacy of current income (52.9%) emerged as another significant driver with a weighted percentage of 77.3%, consistent with Xu et al. (2020), who highlighted that low-income levels are one of the primary factors motivating rural-to-urban migration [21].

The quality of public services also affects migration decisions. Poor educational services (48.5%) and health services (48.5%) in villages ranked among the top reasons, with weighted percentages of 75.0% and 75.3%, respectively. These results are supported by Selod et al. (2021), who found that inadequate infrastructure and services in rural regions push many families to seek better living conditions in urban areas [52].

Table 3: Weighted percentage for the migration motivations under land degradation

Why do you want to change your current accommodation place?	Scale						Total marks	Weighted percent-age%	Rank
	agree		Neutral		Disagree				
	F	%	F	%	F	%			
To find a suitable job opportunity in another governorate	1249	70.1	220	12.3	313	17.6	4500	84.3%	1
To transfer my job to another governorate	893	50.1	425	23.8	464	26	3993	74.7%	8
To find a higher wage or a salary increase there	1191	66.8	271	15.2	320	18	4435	83.0%	2
To open a project with a higher income there	1112	62.4	346	19.4	324	18.2	4352	81.3%	3
The cost of living here is expensive	691	38.8	523	29.3	568	31.9	3687	69.0%	12

I can't find a job in my village	857	48.1	434	24.4	491	27.6	3930	73.7%	11
My salary or monthly income in the village is not enough for me	943	52.9	472	26.5	367	20.6	4140	77.3%	4
I opened a project in the village, and its income is not good	633	35.5	461	25.9	688	38.6	3509	65.7%	13
I don't know how to work more than one job in my village	859	48.2	456	25.6	467	26.2	3956	74.0%	10
To find clean housing with reasonable prices in another governorate	876	49.2	436	24.5	470	26.4	3970	74.3%	9
The educational services in the village are not good	865	48.5	493	27.7	424	23.8	4005	75.0%	7
The health services in the village are not good	864	48.5	514	28.8	404	22.7	4024	75.3%	6
The entertainment services in the village are not good	937	52.6	438	24.6	407	22.8	4094	76.7%	5

Weighted percentage% = $\frac{\text{Total marks}}{N*3}$ *100, where the sample size is (N=1782)

3.3.2 Barriers to Migration

Despite the desire to migrate, several obstacles prevent rural residents from moving, as shown in **Table 4**. The most significant barrier was the difficulty of moving to a new place, reported by 54.1% of respondents, with a weighted percentage of 78.0%. This finding aligns with Selod et al. (2021) observation that rural households face logistical and financial challenges when relocating [52]. Family objection (54.2%) and high moving costs (52.7%) were the next most prominent obstacles, both ranked third with a weighted percentage of 76.3%. These results are consistent with Zohry (2005), who found that family ties and financial constraints are key barriers to migration in rural Egypt [30].

Other significant obstacles included the lack of suitable housing (51.6%) and the fear of social isolation (51.4%), with weighted percentages of 76.7% and 76.0%, respectively. These findings align

with Xiang et al. (2016), who reported that rural migrants often struggle to integrate into new communities due to the lack of social networks and affordable housing options [57].

Table 4 Weighted percentage for the migration barriers under land degradation

What might be an obstacle for you to leave your village and work or live in another province?	Scale: agree F	agree %	Neutral F	Neutral %	Disagree F	Disagree %	Total marks	Weighted percentage%	Rank
Difficulty of moving to the new place	964	54.1	452	25.4	366	20.5	4162	78.0%	1
Lack of suitable housing in the new place	919	51.6	482	27	381	21.4	4102	76.7%	2
Cost of moving	940	52.7	425	23.8	417	23.4	4087	76.4%	3
Family objection	966	54.2	360	20.2	456	25.6	4074	76.2%	4
I might feel isolated and alone in the new place	916	51.4	447	25.1	419	23.5	4061	76.0%	5
Lack of friends and relatives in the new place	896	50.3	465	26.1	421	23.6	4039	75.7%	6
Distance of travel	831	46.6	517	29	434	24.4	3961	74.0%	7
Lack of continuous transportation to the new place	827	46.4	498	27.9	457	25.6	3934	73.7%	8

3.3.3 Motivations and Barriers Behind Migration Decisions

We estimated the impact of the demographic and economic characteristics of the respondents on their willingness to relocate based on the assumptions of PMT, highlighting the motivations and barriers behind migration decisions. Therefore, we constructed a composite variable for the motivation and another for the barriers with three levels (high, medium, and low); we considered the medium as a reference in our estimations. Through this variable, we estimated the perceived intention of respondents to relocate as a result of the environmental change and low agricultural income in the selected area, based on their socioeconomic characteristics. The determinants that influence whether individuals are motivated to migrate or face barriers to relocation are summarized in **Tables 5 and 6**. The results reveal that both demographic and economic characteristics significantly affect migration intentions, although the magnitude and direction of these effects vary between the motivation and barrier models.

Model Fit

Both models demonstrated acceptable goodness-of-fit. The Motivation model reported −2 Log Likelihood = 1845.31 and Chi-Square = 191.46 (df = 62, $p < 0.001$), with Pseudo R^2 values of Cox & Snell = 0.167, Nagelkerke = 0.195, and McFadden = 0.094. The Obstacles model yielded −2 Log Likelihood = 1935.88 and Chi-Square = 129.97 (df = 62, $p < 0.001$), with Pseudo R^2 values of Cox & Snell = 0.117, Nagelkerke = 0.136, and McFadden = 0.063. These results indicate a moderate explanatory capacity, appropriate for social-science datasets.

Demographic Factors, Age emerged as a significant predictor of migration intention in both models. In the *Motivation* model, the odds ratio (Exp(B) = 1.046, $p < 0.01$) indicates that as respondents grow older, their likelihood of considering migration slightly increases. Similarly, in the *Obstacles* model, Exp(B) = 1.023 ($p < 0.05$) shows that older individuals perceive fewer barriers to migration. These results contrast with earlier evidence suggesting that younger people are generally more mobile and willing to migrate for better opportunities. At the same time, older individuals tend to remain in their communities because of established livelihoods and social networks [29,71]. However, in rural Egypt, age can also reflect financial independence and accumulated capital, which may facilitate migration rather than deter it. Gender significantly influenced migration obstacles. In the *Obstacles* model, females were more likely to experience migration motivation (Exp(B) = 1.589, $p < 0.10$). This pattern aligns with studies highlighting that women in rural areas often view migration as a pathway to greater autonomy and improved employment opportunities [85,86]. Although men usually maintain stronger ties to land ownership and agricultural work, limited local opportunities for women often drive them toward urban or overseas migration [87]. These findings emphasize that gendered access to resources and employment remains a central determinant of migration behavior in rural Egypt.

Education was a significant determinant across several categories in the *Obstacles* model. Respondents with university or post-university education exhibited a lower Exp(B) = 0.424 ($p < 0.01$), while those with intermediate-level qualifications (institutes) recorded Exp(B) = 0.349 ($p < 0.01$). Conversely, illiterate respondents showed Exp(B) = 0.497 ($p < 0.05$), implying that lower education levels are associated with more substantial obstacles to migration. These results indicate that higher

educational attainment facilitates migration decisions, consistent with findings that education enhances awareness of opportunities and mobility [85]. Similar trends have been reported among Egyptian youth, where educated individuals pursue migration for professional development or improved income prospects [29].

Table 5. Overall Effect of the Main Variables on Migration Intention (Motivation and Barriers)

	**Motivation**			**Barriers**		
Effect	Model Fitting Criteria (-2 Log Likelihood of Reduced Model)	Chi-Square	df	Model Fitting Criteria (-2 Log Likelihood of Reduced Model)	Chi-Square	df
Intercept	1845.308[a]	0.000		1935.884[a]	0.000	
Age	1873.284	27.976***	2	1935.884[a]*	0.000	2
Gender	1854.039	8.732**	2			
Educational Status	1865.923	20.615**	10			
Job	1863.314	18.006**	8			
Income Adequacy	1867.576	22.268***	4			
Monthly income				1944.038**	8.154	2
Marital Status				1950.504**	14.620	6
Change in land productivity.				1951.118***	15.234	4
Change in village conditions now compared to 10 years ago.				1944.920*	9.036	4
Model Fitting (-2 Log Likelihood)	1845.31	191.46***	62	1935.88	129.97***	62
		**Pseudo R-Square**				
Cox and Snell		0.167			0.117	
Nagelkerke		0.195			0.136	
McFadden		0.094			0.063	

*** Significant at $p < 0.01$ ** Significant at $p < 0.05$ * Significant at $p < 0.10$

The chi-square statistic is the difference in -2 log-likelihoods between the final model and a reduced model. The reduced model is formed by omitting an effect from the final model. The null hypothesis is that all parameters of that effect are 0.

a. This reduced model is equivalent to the final model because omitting the effect does not increase the degrees of freedom.

Economic Factors include income and income adequacy**,** with income adequacy being significant in both models. In the *Motivation* model, households reporting "not enough" income had Exp(B) = 0.520 ($p < 0.05$), while those reporting "enough" income recorded Exp(B) = 0.490 ($p < 0.01$). In the *Obstacles* model, inadequate income substantially increased the likelihood of migration (Exp(B) = 1.734, $p < 0.01$). These findings highlight that both objective and perceived income conditions influence migration, consistent with Gray et al. (2012) and Restelli (2023) [16,87]. Monthly income was also significant (Exp(B) = 1.000, $p < 0.01$), indicating that higher income slightly reduces the probability of facing migration obstacles. Such results align with classical migration theory (Todaro, 1969), which links income disparities and job opportunities to mobility decisions [79]. In Egypt, low-income households often rely on migration to diversify income sources, while skilled workers are pulled abroad by higher wages in the EU and Gulf countries [88].

Agricultural Engagement**:** Employment in agriculture and its share of household income had a negative influence on migration intention. In the Obstacles model, the agricultural income shares yielded Exp(B) = 0.357 ($p < 0.01$), implying that stronger agricultural dependence reduces migration propensity. Similarly, employment in private agricultural work (Exp(B) = 0.328, $p < 0.05$) limited migration motivation. These results are consistent with Gray et al. (2012), who observed that individuals closely tied to farming are less likely to migrate due to attachment to land and local livelihoods [16]. However, environmental pressures—such as declining soil fertility and water scarcity—can offset this stability, especially among younger farmers [16,88].

Table 6. Significant Parameter Estimates for Motivation and Barriers Models

**Parameter Estimates**	**Motivation**				**Barriers**			
	**(1) Low**		**(3) High**		**(1) Low**		**(3) High**	
	Std. Er.	Exp(B)	Std. Er.	Exp(B)	Std. Er.	Exp(B)	Std. Er.	Exp(B)
Intercept	1.027		0.694		0.963			
Age	0.009	1.046***			0.009	1.023**		
[Income adequacy=1] Not enough	0.306	0.520**	0.216	1.734***				
[Income adequacy=2] Enough	0.257	0.490***						
Agriculture's share of income			0.276	0.590*				
Monthly income			0.000	1.000*			0.000	1.0***

[Gender=1] Females			0.266	1.589*				
[Educational Status=1] Illiterate			0.304	0.497**				
[Educational Status=4] University and post-university			0.248	0.424***				
[Educational Status=5] Institute (above intermediate)			0.368	0.349***				
[Job=3] government employee and work in agriculture			0.373	0.513*				
[Job= 7] Private sector and work in agriculture	0.454	0.328**						
Agricultural share of total income					0.387	0.357***		
[Changes in land productivity =2] As it was 10 years ago					0.303	2.228***		
[Living conditions of the farmer now compared to 10 years ago= 1] Now worse					0.268	0.643*	0.196	0.660**
[Living conditions of the farmer now compared to 10 years ago= 2] As it was 10 years ago							0.252	0.598**
[Change in village conditions now compared to 10 years ago =2.00] As it was 10 years ago					0.321	0.541*	0.216	0.635**
[Agri-land ownership =0] No							0.693	3.815*
[Changes in land productivity =1] Now less than 10 years ago							0.210	1.678**
[Changes in land productivity =2] As it was 10 years ago							0.225	1.456*

a. The reference category is: 2.

b. This parameter is set to zero because it is redundant.

*** Values are significant at P = 0.01, ** Values are significant at P = 0.05, * Values are significant at P = 0.1

Changes in Land Productivity: The Obstacles model showed that changes in land productivity significantly influenced migration barriers. Respondents reporting lower productivity compared with ten years ago were more likely to face migration pressures (Exp(B) = 1.678, $p < 0.05$). At the same time, those whose productivity remained stable also exhibited a moderate increase in migration likelihood (Exp(B) = 1.456, $p < 0.10$). These findings suggest that any stagnation or decline in agricultural productivity acts as a push factor, heightening migration intentions. Conversely, productivity improve-

ments strengthen local economic resilience and reduce migration risks. Similar evidence has been documented in Egypt and Bangladesh, where soil salinity, desertification, and land degradation have undermined rural livelihoods and triggered out-migration [16]. Perceived improvements in living standards and village conditions significantly lowered migration obstacles. For example, respondents reporting worse living conditions recorded Exp(B) = 0.660 ($p < 0.05$), while those perceiving conditions as unchanged showed Exp(B) = 0.598 ($p < 0.05$). These results highlight the importance of subjective well-being and satisfaction in migration decisions. Similar findings in China's Rural Revitalization program show that improving infrastructure and rural services reduces out-migration [81]. Overall, demographic (age, gender, education), economic (income, income adequacy), agricultural (engagement and productivity), and perceptual factors jointly determine migration intentions in rural Egypt. The results confirm that migration decisions stem from intertwined socio-economic and environmental dynamics rather than single determinants [16,29,88].

Overall, the analysis of migration intentions in Fayoum can be clearly understood within the Protection Motivation Theory (PMT) framework. Agricultural land degradation significantly increases threat appraisal by raising farmers' perceived severity and vulnerability, as declining productivity and livelihood insecurity heighten the risk of staying. At the same time, socio-economic resources such as education, remittances, and land assets serve as coping appraisal factors, shaping households' capacity to adapt locally or migrate successfully. The identified drivers of migration—such as environmental stress and unemployment—correspond to dimensions of threat appraisal, while the barriers—including limited resources, family obligations, and lack of skills—reflect coping constraints. Taken together, these findings demonstrate that migration decisions emerge from the interaction between threat and coping appraisals. This provides an integrated explanation of why some households choose to migrate while others remain, underscoring the value of PMT as a guiding theoretical framework for analyzing environmentally induced migration in developing-country contexts.

4. Conclusions and Policy Implications

The complex interrelationship between population dynamics, agricultural systems, and environmental change creates significant challenges for understanding the drivers of migration. Within the Protection Motivation Theory (PMT) framework, our findings show that threat appraisal is heightened by factors such as declining land productivity, inadequate rural services, and perceptions of economic

insecurity, all of which increase the intention to migrate. At the same time, coping appraisal is shaped by socio-economic resources, including education, remittances, and household assets, which determine whether individuals pursue migration or adapt locally. Demographic and social factors, particularly age, gender, and education, play a significant role, with younger and better-educated individuals, especially women, more likely to migrate in search of autonomy and opportunity.

While absolute income was a significant predictor, the perception of income adequacy demonstrated a more nuanced association with migration intentions, indicating the mediating role of subjective well-being. These results affirm the explanatory sufficiency and robustness of PMT in this context. Furthermore, they underscore the necessity of integrating socio-economic and institutional dimensions into the PMT framework for a comprehensive model. Consequently, effective intervention strategies should aim to reduce threat appraisal by implementing sustainable land and water management, poverty alleviation programs, and enhanced rural services. Concurrently, coping appraisal can be strengthened through expanded access to education and vocational training, as well as initiatives that support women's entrepreneurship. Such integrated approaches would not only mitigate the drivers of migration but also foster inclusive and sustainable rural development.

The policy implications based on the empirical findings from both models emphasize the necessity of designing integrated rural development policies that directly address the root causes of migration in Egypt’s rural regions. Since migration intention is powerfully shaped by demographic, economic, and agricultural factors, interventions should prioritize youth employment creation, women’s empowerment, and agricultural modernization. Policies that enhance rural education, vocational training, and entrepreneurship, particularly for young and educated individuals, can convert migration pressure into local productivity gains. Strengthening income adequacy through diversified livelihoods, access to credit, and value-added agribusiness initiatives can mitigate economic push factors. Moreover, the significant influence of land productivity and living conditions suggests the need for investment in climate-smart agriculture, irrigation efficiency, and soil salinity management to stabilize agricultural

income and reduce migration caused by environmental degradation. Gender-sensitive measures are equally critical: supporting women's landownership, improving their access to markets, and promoting their participation in decision-making can lower the gendered drivers of migration. Overall, adopting a comprehensive rural revitalization framework, like China's model, would help retain rural populations by improving infrastructure, public services, and local governance, thereby ensuring that migration becomes a choice driven by opportunity rather than necessity.

Author Contributions: Conceptualization, M. Hussien, Ali, Tan and M. Abdelhameed; **methodology**, Tan, M. Abdelhameed, and Ali; **software**, Ali, M. Hussien and M. Abdelhameed; **validation**, Tan, Ali and M. Hussien; **formal analysis**, M. Abdelhameed and Ali; **investigation**, M. Abdelhameed, M. Hussien, and Ali; **resources**, Ali and Tan; **data curation**, M. Hussien, M. Abdelhameed and Ali; **writing—original draft preparation**, M. Abdelhameed and Ali; **writing—review and editing**, M. Hussien, M. Abdelhameed, Ali and Tan; **visualization**, M. Hussien, Tan and M. Abdelhameed; **funding acquisition**, Tan. All authors have read and agreed to the published version of the manuscript.

Funding: This work was supported by The National Natural Science Foundation of China Project: The Economic Transformation and the Development of Regional Agricultural Products Value Chain of ASEAN and China (**71961147002**).

Data Availability Statement: Data will be available based on request.

Acknowledgements: We thank the anonymous reviewers for their valuable help in improving the manuscript.

Conflicts of Interest: Each named author has substantially contributed to conducting the underlying research and drafting this manuscript. Additionally, to the best of our knowledge, the named authors have no conflict of interest, financial or otherwise.

5. References

1. Hermans, K.; McLeman, R. Climate Change, Drought, Land Degradation and Migration: Exploring the Linkages. *Curr Opin Environ Sustain* 2021, *50*, 236–244.
2. Gu, W.; Ma, G.; Wang, R.; Scherer, L.; He, P.; Xia, L.; Zhu, Y.; Bi, J.; Liu, B. Climate Adaptation through Crop Migration Requires a Nexus Perspective for Environmental Sustainability in the North China Plain. *Nat Food* **2024**, *5*, 569–580, doi:10.1038/s43016-024-01008-8.
3. Salerno, J.; Gaughan, A.E.; Warrier, R.; Boone, R.; Stevens, F.R.; Keys, P.W.; Mangewa, L.J.; Mombo, F.M.; de Sherbinin, A.; Hartter, J.; et al. Rural Migration under Climate and Land Systems Change. *Nat Sustain* **2024**, doi:10.1038/s41893-024-01396-6.
4. McLeman, R. *Migration and Land Degradation: Recent Experience and Future Trends*; Global Land Outlook ; Global Land Outlook working paper. UNCCD, 2017;
5. Liu, Y.; Zhang, R.; Li, M.; Zhou, C. What Factors Influence Rural-to-Urban Migrant Peasants to Rent out Their Household Farmland? Evidence from China's Pearl River Delta. *Land (Basel)* **2020**, *9*, 1–20, doi:10.3390/land9110418.
6. Davis, J.; Lopez-Carr, D. Migration, Remittances and Smallholder Decision-Making: Implications for Land Use and Livelihood Change in Central America. *Land use policy* **2014**, *36*, 319–329, doi:10.1016/j.landusepol.2013.09.001.
7. Nguyen, C.V.; Nguyen, H.Q. Do Internal and International Remittances Matter to Health, Education and Labor of Children and Adolescents? The Case of Vietnam. *Child Youth Serv Rev* **2015**, *58*, 28–34, doi:10.1016/j.childyouth.2015.09.002.
8. Hermans, K.; Müller, D.; O'Byrne, D.; Olsson, L.; Stringer, L.C. Land Degradation and Migration. *Nat Sustain* **2023**, *6*, 1503–1505, doi:10.1038/s41893-023-01231-4.
9. Gray, C.; Bilsborrow, R. Environmental Influences on Human Migration in Rural Ecuador. *Demography* **2013**, *50*, 1217–1241, doi:10.1007/s13524-012-0192-y.
10. Appadurai, A. The Capacity to Aspire: Culture and the Terms of Recognition. *Cultural politics in a global age: Uncertainty, solidarity and innovation* **2007**, 29.
11. Johnson, R.L. Reversing Channels and Unsettling Binaries: Rethinking Migration and Agrarian Change under Expanded Border and Immigration Enforcement. *Land (Basel)* **2021**, *10*, 1–19, doi:10.3390/land10030228.
12. De Brauw, A. Migration Out of Rural Areas and Implications for Rural Livelihoods. **2019**, doi:10.1146/annurev-resource-100518.
13. Soto Nishimura, A.; Czaika, M. Exploring Migration Determinants: A Meta-Analysis of Migration Drivers and Estimates. *J Int Migr Integr* **2024**, *25*, 621–643, doi:10.1007/s12134-023-01091-z.
14. International Organization for Migration (IOM) *World Migration Report 2020*; IOM World Migration Report; United Nations, 2019; ISBN 9789290687894.

15. Kafando, W.A.; Sakurai, T. Armed Conflicts and Household Food Insecurity: Effects and Mechanisms. *Agricultural Economics* **2024**, doi:10.1111/agec.12814.
16. Gray, C.L.; Mueller, V. Natural Disasters and Population Mobility in Bangladesh. *Proceedings of the National Academy of Sciences* **2012**, *109*, 6000–6005, doi:10.1073/pnas.1115944109.
17. Sedova, B.; Kalkuhl, M. Who Are the Climate Migrants and Where Do They Go? Evidence from Rural India. *World Dev* **2020**, *129*, 104848, doi:https://doi.org/10.1016/j.worlddev.2019.104848.
18. Adger, W.N.; Kelly, P.M.; Winkels, A.; Huy, L.Q.; Locke, C. Migration, Remittances, Livelihood Trajectories, and Social Resilience. *AMBIO: A Journal of the Human Environment* **2002**, *31*, 358–366, doi:10.1579/0044-7447-31.4.358.
19. Henry, S.; Schoumaker, B.; Beauchemin, C. The Impact of Rainfall on the First Out-Migration: A Multi-level Event-History Analysis in Burkina Faso. *Population and Environment - POP ENVIRON* **2004**, *25*, 423–460, doi:10.1023/B:POEN.0000036928.17696.e8.
20. Liu, Y.; Li, J.; Yang, Y. Strategic Adjustment of Land Use Policy under the Economic Transformation. *Land use policy* **2018**, *74*, 5–14, doi:https://doi.org/10.1016/j.landusepol.2017.07.005.
21. Xu, D.; Yong, Z.; Deng, X.; Zhuang, L.; Qing, C. Rural-Urban Migration and Its Effect on Land Transfer in Rural China. *Land (Basel)* **2020**, *9*, doi:10.3390/land9030081.
22. Al-Maruf, A.; Kanak Pervez, A.K.M.; Sarker, P.K.; Rahman, M.S.; Ruiz-Menjivar, J. Exploring the Factors of Farmers' Rural–Urban Migration Decisions in Bangladesh. *Agriculture (Switzerland)* **2022**, *12*, doi:10.3390/agriculture12050722.
23. Awad, I. The Political Economy of Egyptian Migration to Europe in the 2020s. In *IMISCOE Research Series*; Springer Science and Business Media B.V., 2024; Vol. Part F1581, pp. 347–364.
24. Moore, M.; Wesselbaum, D. Climatic Factors as Drivers of Migration: A Review. *Environ Dev Sustain* **2023**, *25*, 2955–2975, doi:10.1007/s10668-022-02191-z.
25. Soto Nishimura, A.; Czaika, M. Exploring Migration Determinants: A Meta-Analysis of Migration Drivers and Estimates. *J Int Migr Integr* **2024**, *25*, 621–643, doi:10.1007/s12134-023-01091-z.
26. Ajaero, C.K.; Onokala, P.C. The Effects of Rural-Urban Migration on Rural Communities of Southeastern Nigeria. *Int J Popul Res* **2013**, *2013*, 1–10, doi:10.1155/2013/610193.
27. Majeed, A.; Muhammad, Z. Salinity: A Major Agricultural Problem—Causes, Impacts on Crop Productivity and Management Strategies. In *Plant Abiotic Stress Tolerance: Agronomic, Molecular and Biotechnological Approaches*; Hasanuzzaman, M., Hakeem, K.R., Nahar, K., Alharby, H.F., Eds.; Springer International Publishing: Cham, 2019; pp. 83–99 ISBN 978-3-030-06118-0.
28. Schwilch, G.; Liniger, H.P.; Hurni, H. Sustainable Land Management (SLM) Practices in Drylands: How Do They Address Desertification Threats? *Environ Manage* **2014**, *54*, 983–1004, doi:10.1007/s00267-013-0071-3.
29. Abdelwahed, A.; Goujon, A.; Jiang, L. The Migration Intentions of Young Egyptians. *Sustainability (Switzerland)* **2020**, *12*, 1–38, doi:10.3390/su12239803.
30. Zohry, A. *Interrelationships between Internal and International Migration in Egypt: A Pilot Study*; 2005;
31. Almulhim, A.I.; Alverio, G.N.; Sharifi, A.; Shaw, R.; Huq, S.; Mahmud, M.J.; Ahmad, S.; Abubakar, I.R. Climate-Induced Migration in the Global South: An in Depth Analysis. *npj Climate Action* **2024**, *3*, doi:10.1038/s44168-024-00133-1.

32. Nagabhatla, N.; Pouramin, P.; Brahmbhatt, R.; Fioret, C.; Glickman, T.; Newbold, K.B.; Smakhtin, V. Water and Migration: A Global Overview. *UNU-INWEH report series* **2020**, *10*.
33. Zaveri, E.; Russ, J.; Khan, A.; Damania, R.; Borgomeo, E.; Jägerskog, A. *Ebb and Flow, Volume 1*; The World Bank, 2021; ISBN 978-1-4648-1745-8.
34. Mahmoud, M.A. Impact of Climate Change on the Agricultural Sector in Egypt. In *Conventional Water Resources and Agriculture in Egypt*; Negm, A.M., Ed.; Springer International Publishing: Cham, 2019; pp. 213–227 ISBN 978-3-319-95065-5.
35. Viala, E. Water for Food, Water for Life a Comprehensive Assessment of Water Management in Agriculture. *Irrigation and Drainage Systems* **2008**, *22*, 127–129, doi:10.1007/s10795-008-9044-8.
36. Zaveri, E.; Russ, J.; Khan, A.; Damania, R.; Borgomeo, E.; Jägerskog, A. *Ebb and Flow: Volume 1. Water, Migration, and Development*; World Bank Publications: Washington, DC, 2021; ISBN 1464817472.
37. Jha, C.K.; Gupta, V.; Chattopadhyay, U.; Amarayil Sreeraman, B. Migration as Adaptation Strategy to Cope with Climate Change: A Study of Farmers' Migration in Rural India. *Int J Clim Chang Strateg Manag* **2018**, *10*, 121–141.
38. Pingali, P.L. Green Revolution: Impacts, Limits, and the Path Ahead. *Proceedings of the national academy of sciences* **2012**, *109*, 12302–12308.
39. Elmoghazy, A.M.; Elshenawy, M.M. Sustainable Cultivation of Rice in Egypt. In *Handbook of Environmental Chemistry*; Springer Verlag, 2019; Vol. 76, pp. 119–144.
40. Morton, J.F. The Impact of Climate Change on Smallholder and Subsistence Agriculture. *Proceedings of the national academy of sciences* **2007**, *104*, 19680–19685.
41. Eshetu, F.; Beshir, M. Dynamics and Determinants of Rural-Urban Migration in Southern Ethiopia. *J Dev Agric Econ* **2017**, *9*, 328–340.
42. Selod, H.; Shilpi, F. Rural-Urban Migration in Developing Countries: Lessons from the Literature. *Reg Sci Urban Econ* **2021**, *91*, doi:10.1016/j.regsciurbeco.2021.103713.
43. Asfaw, W.; Tolossa, D.; Zeleke, G. Causes and Impacts of Seasonal Migration on Rural Livelihoods: Case Studies from Amhara Region in Ethiopia. *Norsk Geografisk Tidsskrift–Norwegian Journal of Geography* **2010**, *64*, 58–70.
44. Nweke, A.C. Rural-Urban Migration in Nigeria, Implication on the Development of the Society: Anambra State as the Focus of the Study. *Journal of Public Administration and Governance* **2019**, *9*, 209–216.
45. United Nations *International Migration 2020 Highlights*; 2021;
46. Geddes, A.; Adger, W.N.; Arnell, N.W.; Black, R.; Thomas, D.S.G. Migration, Environmental Change, and the 'Challenges of Governance.' *Environ Plann C Gov Policy* **2012**, *30*, 951–967.
47. Renaud, F.G.; Dun, O.; Warner, K.; Bogardi, J. A Decision Framework for Environmentally Induced Migration. *International Migration* **2011**, *49*, e5–e29.
48. Qin, H.; Liao, T.F. Labor Out-Migration and Agricultural Change in Rural China: A Systematic Review and Meta-Analysis. *J Rural Stud* **2016**, *47*, 533–541, doi:10.1016/j.jrurstud.2016.06.020.
49. Qin, H.; Liao, T.F. Labor Out-Migration and Agricultural Change in Rural China: A Systematic Review and Meta-Analysis. *J Rural Stud* **2016**, *47*, 533–541, doi:10.1016/j.jrurstud.2016.06.020.
50. Bodvarsson, Ö.B.; Van den Berg, H. The Determinants of International Migration: Theory. In *The Economics of Immigration*; Springer New York: New York, NY, 2013; pp. 27–57.

51. Fafchamps, M.; Shilpi, F. Determinants of the Choice of Migration Destination. *Oxf Bull Econ Stat* **2013**, *75*, 388–409, doi:https://doi.org/10.1111/j.1468-0084.2012.00706.x.
52. Selod, H.; Shilpi, F. Rural-Urban Migration in Developing Countries: Lessons from the Literature. *Reg Sci Urban Econ* **2021**, *91*, doi:10.1016/j.regsciurbeco.2021.103713.
53. Bazzi, S.; Gaduh, A.; Rothenberg, A.D.; Wong, M. Unity in Diversity? How Intergroup Contact Can Foster Nation Building. *American Economic Review* **2019**, *109*, 3978–4025.
54. Taylor, J.E.; Martin, P.L. Human Capital: Migration and Rural Population Change. *Handbook of agricultural economics* **2001**, *1*, 457–511.
55. Luo, C.; Yan, X.; Wang, B.; Ali, A. Long-Term Adaptation Mitigates the Promotion Effect of Air Pollution on Short-Term Population Movements. *Front Environ Sci* **2025**, *13*, 1620499, doi:10.3389/FENVS.2025.1620499/BIBTEX.
56. Lu, H.; Kandilov, I.T.; Zhu, R. The Impact of Internal Migration on the Health of Rural Migrants: Evidence from Longitudinal Data in China. *J Dev Stud* **2020**, *56*, 840–855.
57. Xiang, A.O.; Jiang, D.; Zhong, Z. The Impact of Rural–Urban Migration on the Health of the Left-behind Parents. *China Economic Review* **2016**, *37*, 126–139.
58. De Jong, G.F. Expectations, Gender, and Norms in Migration Decision-Making. *Popul Stud (NY)* **2000**, *54*, 307–319.
59. Lee, E.S. A Theory of Migration. *Demography* **1966**, *3*, 47–57, doi:10.2307/2060063/METRICS.
60. Ravenstein, E.G. The Laws of Migration. *Journal of the Statistical Society of London* **1885**, *48*, 167–227, doi:10.2307/2979181.
61. De Haas, H. Migration and Development: A Theoretical Perspective. *International Migration Review* **2010**, *44*, 227–264, doi:10.1111/j.1747-7379.2009.00804.x.
62. de Haas, H. A Theory of Migration: The Aspirations-Capabilities Framework. *Comp Migr Stud* **2021**, *9*, doi:10.1186/s40878-020-00210-4.
63. Rogers, R.W. A Protection Motivation Theory of Fear Appeals and Attitude Change1. *J Psychol* **1975**, *91*, 93–114, doi:10.1080/00223980.1975.9915803.
64. Maddux, J.E.; Rogers, R.W. Protection Motivation and Self-Efficacy: A Revised Theory of Fear Appeals and Attitude Change. *J Exp Soc Psychol* **1983**, *19*, 469–479, doi:https://doi.org/10.1016/0022-1031(83)90023-9.
65. Krejcie, R. V.; Morgan, D.W. Determining Sample Size for Research Activities. *Educ Psychol Meas* **1970**, *30*, 607–610, doi:10.1177/001316447003000308.
66. Cochran, W.G. *Sampling Techniques*; john wiley & sons, 1977; ISBN 8126515244.
67. Wooldridge, J.M. *Econometric Analysis of Cross Section and Panel Data*; MIT press, 2010; ISBN 0262296799.
68. Hua, C.; Choi, Y.J.; Shi, Q. Companion to BER 642: Advanced Regression Methods. *Bookdown. Retrieved August* **2021**, *5*, 2021.
69. Deininger, K.; Jin, S. Tenure Security and Land-Related Investment: Evidence from Ethiopia. *Eur Econ Rev* **2006**, *50*, 1245–1277, doi:https://doi.org/10.1016/j.euroecorev.2005.02.001.
70. Radwan, S.; Lee, E. *Agrarian Change in Egypt: An Anatomy of Rural Poverty*; Routledge, 2022; ISBN 1003313418.
71. Mulder, C.H.; Cooke, T.J. Family Ties and Residential Locations. *Popul Space Place* **2009**, *15*, 299–304.
72. White, M.J.; Lindstrom, D.P. Internal Migration. In *Handbook of population*; Springer, 2005; pp. 311–346.

73. Chant, S. Households, Gender and Rural-Urban Migration: Reflections on Linkages and Considerations for Policy. *Environ Urban* **1998**, *10*, 5–22.
74. Lagakos, D. Urban-Rural Gaps in the Developing World: Does Internal Migration Offer Opportunities? *Journal of Economic Perspectives* **2020**, *34*, 174–192, doi:10.1257/jep.34.3.174.
75. Yu, Z.; Zhang, H.; Tao, Z.; Liang, J. Amenities, Economic Opportunities and Patterns of Migration at the City Level in China. *Asian and Pacific Migration Journal* **2019**, *28*, 3–27, doi:10.1177/0117196819832054.
76. Nguyen, C.V.; Nguyen, H.Q. Do Internal and International Remittances Matter to Health, Education and Labor of Children and Adolescents? The Case of Vietnam. *Child Youth Serv Rev* **2015**, *58*, 28–34, doi:10.1016/j.childyouth.2015.09.002.
77. Skeldon, R. Interlinkages between Internal and International Migration and Development in the Asian Region. *Popul Space Place* **2006**, *12*, 15–30, doi:https://doi.org/10.1002/psp.385.
78. Chen, Y.; Luo, P.; Chang, T. Urbanization and the Urban-Rural Income Gap in China: A Continuous-wavelet Coherency Analysis. *Sustainability (Switzerland)* **2020**, *12*, doi:10.3390/su12198261.
79. Todaro, M.P.; Maruszko, L. Illegal Migration and US Immigration Reform: A Conceptual Framework. *Popul Dev Rev* **1987**, 101–114.
80. Zapata-Barrero, R.; Awad, I. Introduction: Mediterranean Migration Studies – A Research Agenda for the Coming Years. In *IMISCOE Research Series*; Springer Science and Business Media B.V., 2024; Vol. Part F1581, pp. 1–7.
81. Ren, T.; Wang, N.; Xiao, H.; Zhou, Z. Efficiency of Funding to Rural Revitalization and Regional Heterogeneity of Technologies in China: Dynamic Network Nonconvex Metafrontiers. *Socioecon Plann Sci* **2024**, *92*, doi:10.1016/j.seps.2024.101825.
82. Ghio, D.; Goujon, A.; Natale, F.; Alfredo, A.; Petroliagkis, T. Assessing Populations Exposed to Climate Change: A Focus on Africa in a Global Context. *Popul Environ* **2023**, *45*, doi:10.1007/s11111-023-00439-y.
83. King, R.; Skeldon, R. 'Mind the Gap!'Integrating Approaches to Internal and International Migration. *J Ethn Migr Stud* **2010**, *36*, 1619–1646.
84. Radwan, S.; Lee, E. *Agrarian Change in Egypt: An Anatomy of Rural Poverty*; Routledge, 2022; ISBN 1003313418.
85. Aydemir, A.B.; Kırdar, M.G.; Torun, H. The Effect of Education on Internal Migration of Young Men and Women: Incidence, Timing, and Type of Migration. *Labour Econ* **2022**, *74*, 102098, doi:10.1016/J.LABECO.2021.102098.
86. Anastasiadou, A.; Kim, J.; Sanlitürk, E.; de Valk, H.A.G.; Zagheni, E. Gender Differences in the Migration Process: A Narrative Literature Review. *Popul Dev Rev* **2024**, *50*, 961–996, doi:10.1111/PADR.12677.
87. Restelli, G. Development and International Migration: The Effect of Income on Regular and Irregular Migration Intentions to Europe. *Popul Dev Rev* **2023**, *49*, 135–174, doi:10.1111/PADR.12525.
88. Brodmann, S.; Pouget, Y.; Gatti, R. *Labor Mobility in the Middle East and North Africa: Challenges and Opportunities*; World Bank, Washington, DC, 2010;

Appendix

We attached them as a Supporting Information file, which contains the following;

Table A.1: Selected villages in the *Itsa* districts

Table A.2: Descriptive statistics of the sample

Table A.3: Farming system Characteristics and migration intention of the selected respondents N= (1782)

Click here to access/download
Supporting Information
Supporting Information.docx

Type of the Paper (Article)

Agricultural Land Degradation Consequences as a Migration Driver in ~~Developing Countries~~Egypt

Muhammad Hussien[1†], Abdelrahman Ali[2, 3*†], Yanwen Tan[2*], Mohamed Abdelhameed [3]

[1] Department of Geography, Faculty of Arts, Fayoum University, 63514, Egypt mhb00@fayoum.edu.eg

[2] School of Economics and Management, South China Agricultural University, Guangzhou, 510642, China aaa31@fayoum.edu.eg

[3] Department of Agricultural Economics, Faculty of ~~Agricultural~~Agriculture, Fayoum University, 63514, Egypt maa20@fayoum.edu.eg

***** Correspondence: Tan tanyw@scau.edu.cn, Ali aaa31@fayoum.edu.eg

†These authors contributed equally to this work and should be considered co-first authors

Abstract

Environmental migration is a global phenomenon where increasing soil salinity leads to decreased productivity and income, subsequently increasing migration intention (MI) in the affected regions. This study investigates how agricultural land degradation (ALD), particularly soil salinity ~~reducing~~, reduces productivity and ~~,~~ influences MIs in Egypt. ~~,~~ Employing Protection Motivation Theory (PMT) as a framework, we analyzed primary data from 1,782 respondents using a multinomial logistic model. The empirical findings revealed that socioeconomic characteristics, agricultural engagement, and ALD significantly shape and determine MI~~migration willingness~~. Furthermore, over 35.8% of respondents reported a decline in land productivity in the past decade due to environmental change, with 22.4% actively seeking relocation. Among those willing to migrate, the capital Cairo (40.2%) and international destinations (29.4%) were the most desired. Additionally, gender, age, and income adequacy were identified as crucial predictors in migration dynamics. The findings challenge conventional demographic assumptions by revealing a significant propensity to migrate among older populations and women, groups often considered less mobile. ~~Primary motivations were access to suitable jobs (70.1%) and higher wages (66.8%), whereas major obstacles were relocation difficulty~~The primary motivations (employment and income) are directly exacerbated by ecological pressures like declining land productivity. Therefore, effective policy must address this nexus to reduce threat appraisal by implementing sustainable land and water management and investing in both environmental adaptation (e.g., soil restoration) and human capital (e.g., education, local economic opportunities) to mitigate displacement pressures and foster sustainable rural resilience.

Keywords: Soil Restoration~~Land Ownership~~; Environmental Stresses; Rural-Urban Migration; ~~Migration~~Protection Motivation Theory ~~Drivers~~; Migration Stream; Rural-urban Displacement

1. Introduction

1.1 Background

Environmental stresses often cause socioeconomic vulnerability~~ that leads to the erosion of household resources, thereby~~, leading to the erosion of household resources and encouraging migration as a survival strategy for many rural residents[1,2]. Migration has been considered a global challenge in recent years, where the interaction between a set of environmental changes and socioeconomic characteristics ~~play~~ plays a role in migration decisions and moving vulnerable communities from one region to another, particularly~~,~~ in developing countries[3,4] . Climate change (CC) is projected to negatively affect from 50 million to one billion people from different regions in 2050, increasing the ~~people's~~ people in movement and migration~~ rate~~, and putting pressure on the natural resources in many areas [1,5]. However, environmental migration has been considered a simple indicator of communities' failure to adopt CC~~, ;~~ ~~but~~nevertheless, the migrants could support their local families left behind (in the origin areas) through remittances, which will be invested to enhance the infrastructure and improve the lives of their families. [6,7]. The pull and push drivers, causes, impacts, and types of migration have been presented in **Fig. 1**.

Currently, the global population redistribution is due to migration and socioeconomic changes, where some regions have become less habitable due to conflicts, climate change, water pollution or scarcity, agricultural land deterioration, and desertification, driving people to seek better lives elsewhere [2,8,9]. ~~In 2019, Egypt was the most prominent African country in terms of migrants, according to the number of people (~4 million citizens) living abroad as migrant labor in the Gulf countries, mainly in Saudi Arabia and the United Arab Emirates~~ People in all countries and cultures strive for satisfaction, well-being, and a good quality of life elsewhere [10,11]. This pursuit of a good quality of life changes dynamically as it is influenced by external stimuli, such as peer interaction or social comparisons of the individual's status with others in similar circumstances [12]. Complex interactions between socioeconomic, political processes, and environmental factors could result in heterogeneous potential migration outcomes [13]. Livelihoods in the degraded regions are closely interconnected with migration, particularly where the people mostly depend on agricultural land [8]. Then, combating land degradation should be considered while designing and implementing the interventions to tackle regional or international migration.

In 2019, Egypt was the most prominent African country in terms of migrants, according to the number of people (~4 million citizens) living abroad as migrant labor in the Gulf countries, mainly in Saudi Arabia and the United Arab Emirates [14]. However, the number of people coming to Egypt from the surrounding Arab countries is increasing annually due to the current civil conflicts in these

countries, which increases the pressure on food demand and food security in Egypt, as it is one of the region's highest food importers [15]. ~~Many~~Several empirical studies across ~~different regions highlight~~ various regions have highlighted the link between land degradation and rural-urban migration [1,4,8]. In Africa, countries ~~like Ethiopia and Niger show that severe land degradation and recurrent droughts force rural populations to migrate to urban centers for~~such as Ethiopia and Niger demonstrate that severe land degradation and recurring droughts compel rural populations to migrate to urban centers in search of better opportunities [16]. In India, studies indicate that land degradation, exacerbated by monsoon variability and water scarcity, contributes to rural outmigration, particularly among smallholder farmers [17]~~, also~~. Additionally, in Mexico, deforestation and soil erosion in rural areas have been identified as significant drivers of migration to urban areas, including ~~in Mexico, deforestation and soil erosion in rural areas have been identified as significant drivers of migration to urban areas and even~~ to the United States [18].

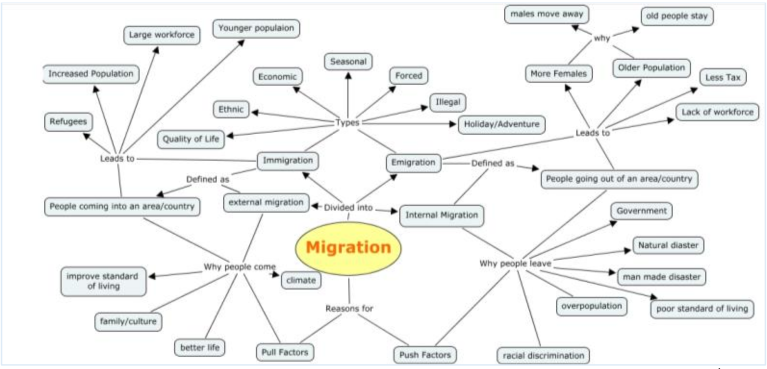

Figure 1 Migration pull and push drivers, causes, and ~~its~~ their impacts.[1]
People migrate in search of better lives or as a result of natural disasters, climate change, and environmental degradation. The migration streams have numerous consequences for both the sender and receiver regions.

Furthermore, some case studies provide detailed insights into the mechanisms by which land degradation drives migration. In the Sahel Region, West Africa, land degradation, due to overgrazing and unsustainable farming practices, has led to significant rural-urban migration, and migrants often move to urban centers or coastal countries seeking employment [19]. While in Northern China, desertification in the Inner Mongolia region has resulted in large-scale rural displacement. Migrants typically move (temporarily or permanently) to the capital cities like Beijing and Tianjin, seeking better living conditions [20,21]. In Bangladesh, riverbank erosion and soil salinity in coastal areas have

Formatted: Font: 11 pt
Formatted: Font: 11 pt
Formatted: Font: 11 pt
Formatted: Font: 11 pt

[1] Migration concept map https://cmapspublic3.ihmc.us/rid=1295355828203_878224076_18749/Migration%20Concept%20Map.cmap

forced many rural inhabitants to migrate to urban slums in Dhaka, highlighting the link between environmental degradation and urban poverty [16,22].

The novelty of the current work could be highlighted through the following points~~;~~ : (1) our focus is to analyze how the interaction between socioeconomic characteristics and environmental change leads to migration intention in developing countries based on primary data. We apply a construct framework, combining the principles of the Protection Motivation Theory (PMT) and a multinomial logistic model, to predict the willingness to migrate under the potential ALD, which significantly contributes to increased migration streams in developing countries. PMT is a novel and creative theoretical lens for migration; we consider complementing PMT with the livelihoods vulnerability framework and/or Push-Pull models to enhance explanatory power. While existing research has explored the relationship between economic factors and migration [23]~~migration~~, few studies have specifically analyzed how ALD influences migrants' decisions, particularly in terms of migration timing [1,3,4,8]. Most prior research has focused on general migration drivers [24,25], whereas our study sheds light on the more specific aspects of the role of climate change in determining when migrants will move from one region to another based on the degree of soil fertility and water availability in their origin rural area. (2) At the same time, we used the constructed framework ~~aim~~ to ~~provide~~ develop ~~evidence~~evidence-based recommendations ~~-~~about the specific push regions ~~based~~grounded on the degree of access to climate change mitigation and adaptation tools in rural areas in Egypt, ~~,~~ which could contribute to future migration-reduction interventions. The result of the current study could best inform policymakers' and international donors' understanding of the environmental migration dynamics to design a strategy for addressing environmentally induced migration resulting from soil and land degradation in developing countries.

The structure of this paper is as follows~~;~~ : the next section is the literature review, and ~~section~~ Section 3 is the methodology and data source. In section 4, we presented the results and discussion, and finally, we highlighted the main conclusion and policy implications.

1.2 Literature Review

Rural-urban migration is a global phenomenon that affects both developing and developed countries and has profound social, economic, and political implications [21,26]. Therefore, examining the factors affecting rural-urban migration, including their motives and barriers, is crucial for researchers, practitioners, and policymakers to understand the migration drivers and develop effective strategies and interventions for migration inhibition. In the following section, we highlight the comparisons and critiques of the existing literature ~~discuss~~ discussing the impacts of (salinity, water shortage, and crop

pattern change) as drivers leading to income decrease and consequently rural-urban and international migration.

A. Land degradation as a rural-urban migration driver

Land degradation is characterized by the decline in land quality and productivity due to various factors such as deforestation, overgrazing, and improper agricultural practices, which significantly impact rural citizens [4,8]. In many developing countries, ALD and the inability to sustain rural livelihoods have compelled individuals and families to seek opportunities in urban areas. According to a Global Assessment of Land Degradation and Improvement report, a quarter of the world's land area is now considered degraded. This degradation is caused by a combination of human activity and climate change, resulting in the loss of fertile land, reduced agricultural productivity, and the displacement of millions of people (GALDI, 2018)[2]. Furthermore, soil salinity (accumulation of soluble salts in the soil) poses a severe threat to agricultural lands, particularly in arid and semi-arid regions like North Africa and Egypt. Salinity reduces soil fertility, thereby decreasing crop yields and agrarian income [27], this degradation compels farmers to abandon their land and seek better opportunities in urban areas. The recent study focused on sustainable land management practices in drylands, highlighting how salinity contributes to land degradation and subsequent migration [28]. Furthermore, soil salinity has a significant impact on agricultural productivity, where the salinity-induced yield losses drive rural populations to migrate to urban centres. This has appeared clearly in many countries in recent years, e.g., Egyptian youth are increasingly migrating from the environmentally affected areas and border villages to the city centers like Cairo and Alexandria, which increases the pressure on the infrastructure in the receiving cities [29,30].

Water access plays a significant role in global migration [31], while water deficit and scarcity are responsible for about 10% of the recent global migration (1 billion migrants) [32,33]. Although the impact of floods as a migration driver (floods have recently forced millions of people to leave their places), its negative consequences are lower than ~~the~~ those of drought as a migration push factor [33]. More than 25% of the global population suffers from extreme water stress, especially in the developing world (more than 85% of people affected by rainfall variability live in low- or middle-income countries) [31]. As Egypt is located in the ~~the~~ North Africa dry region, the agricultural water shortage in rural areas pushes populations towards urban centers, where more than 80% of the water is used for

[2] https://www.isric.org/projects/global-assessment-land-degradation-and-improvement-glada

agriculture[34]. Water scarcity is another critical driver of rural-urban migration, where the over-extraction of groundwater, inefficient irrigation practices, and climate change exacerbate water shortages (which is common in the North African region), reducing sustainable water availability for agricultural activities [35]. As a result, crop yields decline, incomes fall, and rural inhabitants are forced to migrate to cities for better livelihoods [36]. This appears in the statistics, which show the high migration region, mostly from the dry and semi-dry regions [32].

Changes in crop patterns are often driven by environmental degradation and/or market drivers, which ~~will lead~~lead to decreased agricultural productivity and income, where the farmers may be forced to switch from high-value to low-value crops due to changing climatic conditions or soil degradation [2,37]. This shift reduces profitability and makes farming unsustainable (in many cases), prompting rural residents to migrate to urban areas [35]. Some studies analyzed the impacts of the Green Revolution on migration, noting how changes in crop patterns due to environmental stressors affect rural incomes and migration patterns [38]. The same case is in the study area in Fayoum, which was the first city to cultivate rice in Egypt since 640 A.D. [39]. ~~, where rice~~Rice was the main crop for farmers in Fayoum. Still, due to the water supply shortage in this region since the 2000s, the Egyptian government issued a new regulation in 2008 to prohibit rice cultivation in Fayoum, aiming to secure water sufficiency for other field crops in ~~crops and~~ different regions.

While *Morton* (2007) explores the impact of climate change on smallholder agriculture, highlighting how shifts in crop patterns driven by climate variability lead to decreased income and increased migration due to their vulnerability to ~~adopt~~ adapt or mitigate these negative impacts [40].

B. Socioeconomic drivers and impacts of migration

Socioeconomic factors also play a crucial role in understanding migration decisions; in addition to education, better healthcare, and improvements in lifestyle, urban areas often offer better access to such services and opportunities, which attract people from rural areas who want to improve their quality of life [41,42]. In rural-urban migration, economic opportunity is considered the main driver, with migrants seeking better employment opportunities in urban centers, improved living conditions, higher wages, and more diverse jobs that typically do not exist in rural areas [41]. The lack of sustainable livelihoods and economic opportunities in rural areas, such as the lack of enough arable agricultural land and the lack of viable off-farm activities that generate additional income other than agriculture, can be an incentive for individuals to seek new, higher-income employment opportunities in urban areas [43]. Furthermore, the perceived economic prospects and ~~more~~ greater availability of public services in urban areas compared to rural ones may provide a strong incentive for rural residents to move

Formatted: Font: Italic

to urban ~~ones~~ areas [21,44]. According to the International Migration Report, migration can contribute positively to economic growth by filling labor shortages, particularly in sectors with high demand but low local supply of labor (e.g., agriculture & construction) [45]. Furthermore, migrants often send remittances back to their home countries, which can significantly contribute to poverty reduction and improve the living standards of families left behind (World Bank, 2021)[3].

Environmental changes, such as land degradation, climate change, and natural disasters, tend to encourage rural populations to migrate to urban areas [1,46]. Environmental stresses often cause socioeconomic vulnerability that leads to the erosion of household resources by making traditional agricultural livelihoods unsustainable, thereby encouraging migration as a survival strategy for many rural residents [47]. As highlighted in the literature, the interaction between environmental changes and their socio-economic consequences is a complex process to understand the dynamics of migration [1], in which factors such as environmental changes, available adaptation strategies, and the accessibility of migration options play a crucial role in the decision-making process of the rural population [48].

On the other hand, increased migration can strain public services in urban areas such as healthcare and education, especially in areas with high migrant concentration, leading to budgetary challenges for governments (Migration Policy Institute, 2020)[4]. Moreover, cultural differences and perceived competition for resources can sometimes lead to social tensions and conflicts within communities and reduce the average wages due to the experience of the migrants and the high labor supply [33] Furthermore, the pressure on the infrastructure in the receiver cities will reduce ~~its ability to resilience to~~their ability to withstand sudden shocks and also increase the losses during natural disasters such as floods and storms. At the same time, the food producers will decrease over time, which will increase the food import vouchers, especially in developing countries like Egypt. Also ~~it will increase the mind drainage from the rural to the urban, which could reduce the economic growth in the rural area over the~~ , it will increase the migration from rural to urban areas, which could reduce the economic growth in the rural areas over time.

C. Costs of rural-urban migration

Despite the potential benefits of migration, economic barriers such as moving costs, poor creditworthiness, and the risk of unemployment in urban areas can prevent migrants from moving [24,49]. When making a migration decision, potential migrants typically compare the costs associated with

[3] https://www.worldbank.org/en/topic/migrationremittancesdiasporaissues/brief/migration-remittances-data
[4] https://www.migrationpolicy.org/

their decision with the benefits ~~from~~ of migration. Migrants may face different costs, such as travel costs to the destination, subsistence costs when looking for work and housing, and any opportunity costs for activities that remain in the area of origin [25,50,51]. In addition, migrants face other costs due to differences in religion, culture, language, and ethnicity, which together contribute to shaping the migration decision [52]. For this reason, potential migrants prefer to choose a destination with a similar linguistic or ethnic background [51]. On the other hand, other migrants living in communities with different ethnic backgrounds may be able to integrate better than those living in neighbourhoods with few ethnic groups [53]. This is particularly evident for Arab and African people who have migrated to EU countries over the past few decades.

Rural-urban migration often leads to a reduction in the agricultural workforce in rural areas, impacting farm productivity and food security [54]. In urban areas, migrants may compete with locals for jobs, potentially driving down wages in specific sectors and contributing to higher unemployment rates among low-skilled native workers [54]. Furthermore, rapid urbanization due to migration strains urban infrastructure such as housing, transportation, water, and sanitation services, leading to inadequate living conditions in some cities. Moreover, ~~Increased~~ increased urbanization and migration contribute to environmental degradation, including air and water pollution, deforestation, and loss of biodiversity [46,55]. Migration also affects one's physical and mental well-being. For instance, a recent study reveals that younger migrants from rural to urban areas of China report worsening health, which they attribute to shifts in social trust and emotional states [56]. In addition, older parents of migrants who settled in rural areas are more likely to experience health problems [57]. Migration can also result in social disintegration within rural communities as families are separated, leading to breakdowns in traditional support systems [58].

1.3 Theoretical Framework

The conceptualization of migration studies has been historically grounded in *Ravenstein's* 'Laws of Migration' (1885)[59], which first identified systematic patterns of migration flows, and later expanded by *Everett Lee's* Push-Pull Theory (1966)[60], which emphasized the interaction of push factors in origin areas and pull factors in destination areas. These classical theories provide the intellectual foundation upon which more recent approaches, including the Protection Motivation Theory (PMT), are built to explain environmentally induced migration, as described below.

The migration theories investigate the social change processes that are interlinked with human mobility, so many theories and approaches have been used to understand the root causes, motivations,

Formatted: Font: Not Italic, Font color: Black
Formatted: Font color: Black
Formatted: Font: Not Italic, Font color: Black
Formatted: Font: Not Italic, Font color: Black
Formatted: Font: Not Italic, Font color: Black
Formatted: Font: Not Italic, Font color: Black
Formatted: Font color: Black
Formatted: Font: Not Italic, Font color: Black
Formatted: Font: Not Italic, Font color: Black
Formatted: Font: Not Italic, Font color: Black
Formatted: Font: Not Italic, Font color: Black
Formatted: Font: Not Italic, Font color: Black
Formatted: Font: Not Italic, Font color: Black

and gains of migration. For example, De Haas has investigated the aspirations and capabilities of migration within given sets of perceived geographical opportunity structures and differentiates between human mobility's instrumental (means-to-an-end) and intrinsic (directly wellbeing-affecting) dimensions [61,62]. This yields a vision in which moving and staying are seen as complementary manifestations of migratory agency and in which human mobility is defined as people's capability to choose where to live, including the option to stay, rather than as the act of moving or migrating itself [62]. Furthermore, the labor policies in the ~~destinations~~ destination countries play a significant role in facilitating the immigration of highly skilled labor in risky environmental areas where land degradation and negative impacts of climate change push the migrants to move from these areas to another safe one. At the same time, rural citizens may move to nearby cities or migrate to major capital cities, and some may even immigrate to seek opportunities in another country. Climate change and natural disasters tend to encourage rural populations to migrate [1,46].

This study adopted the Protection Motivation Theory (PMT) to interpret the impact of environmental changes and stresses ~~such as land degradation~~, such as land degradation, as a migration driver. The PMT was developed by Rogers in 1975 and modified in 1983 as a framework for analyzing how individuals react to perceived threats [63,64]. PMT initially focused on health-related behaviours, but it can also be modified to describe migration decisions, especially in situations where individuals encounter environmental, economic, or social challenges.

PMT is a novel and creative theoretical lens for migration; we consider complementing PMT with the livelihoods vulnerability framework and/or Push-Pull models to enhance explanatory power. The following outlines how the primary elements of PMT—threat appraisal and coping appraisal—can clarify migration behavior, **as shown in Fig. 2**:

1. Threat Appraisal (Perceived Severity & Vulnerability)

Migration often happens once individuals perceive a substantial threat to their safety, well-being, or livelihood. For example, the farmers who are facing ALD may perceive yield reduction as a severe threat and believe they will face socioeconomic vulnerability if they stay in the origin regions, which encourages many rural residents to migrate [39]. Furthermore, the interaction between environmental changes and their socioeconomic consequences is a complex process [1], in which factors such as environmental changes, available adaptation strategies, and the accessibility of migration options play a crucial role in the decision-making process of the rural population [47], as shown in **Fig. 2**.

2. Coping Appraisal (Response Efficacy & Self-Efficacy)

Although the threat is perceived to be severe, the migration decision depends on the individual's beliefs about how ~~to effectively respond~~to respond effectively. For example, the farmers who are facing ALD may feel capable of migration~~,~~ if relocation to another place offers better income and living conditions, and they have the legal pathways and resources (savings and relatives abroad), ~~(while poor growers may not)~~while poor growers may not.

3. Fear vs. Adaptive Response

Despite the high threat ~~for~~ to the poor farmers in the region with ALD, they may stay if they have ~~deficiency~~ insufficient resources to relocate (low coping appraisal).

4. External & Internal Rewards (Deterrents to Action)

PMT also ~~take into consideration disincentives that might restrict, delay, or reduce willingness of migration under threat, such as (government aid, social support, and attachment to homeland)~~considers disincentives that may restrict, delay, or reduce the willingness to migrate under threat, including government aid, social support, and attachment to one's homeland.~~-~~

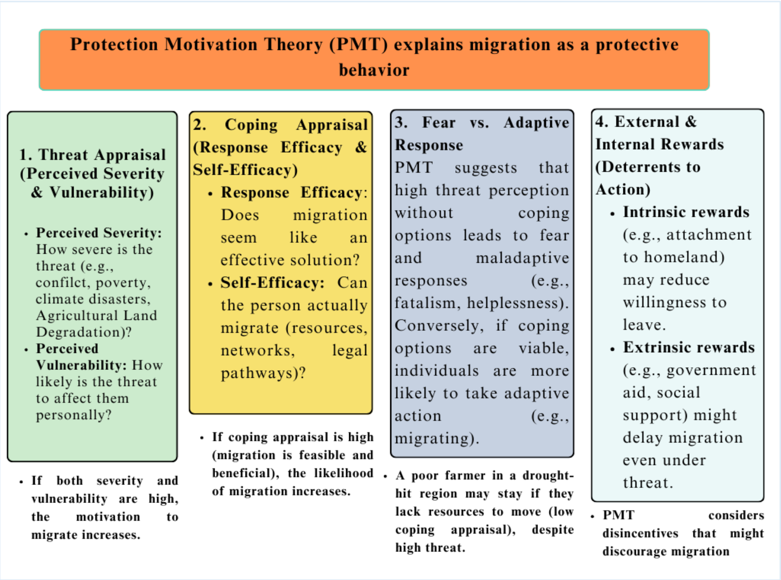

Figure 2 Protection Motivation Theory (PMT) explains migration as a protective behavior.

W~~In Fig. 3, w~~e summarize the potential consequences of ~~the~~ environmental change ~~especially the ALD~~, particularly the ALD, to highlight the interaction between socioeconomic and environmental stress as a driver of migration ~~to highlight the interaction between socioeconomic and environmental~~

Formatted: Indent: First line: 0.35", Line spacing: 1.5 lines

stress as a migration driver. As illustrated in **Figure 3,** the consequences of agricultural land degradation (ALD) on migration intention can be interpreted through the Protection Motivation Theory (PMT). Specifically, ALD increases threat appraisal by raising farmersfarmers' perceived vulnerability (e.g., declining productivity, loss of income) and severity (e.g., livelihood insecurity). At the same time, migration as a coping response depends on coping appraisal, where individuals evaluate their ability to relocate successfully given available resources, skills, and networks. The interaction of these processes, heightened threat appraisal alongside varying levels of coping appraisal, shapes the willingness to migrate depicted in the figure. Thus, **Figure 3** integrates PMT dimensions with empirical evidence of ALD-induced migration intentions.

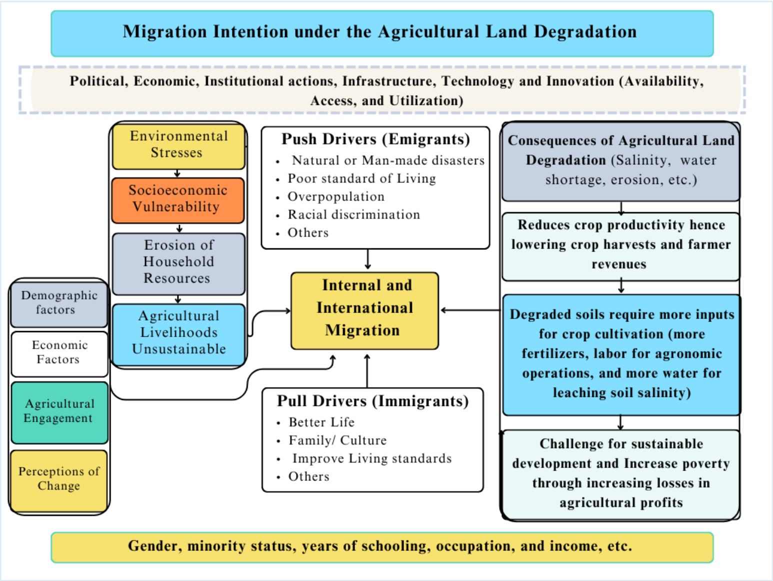

Figure 3 Migration drivers: the push-pull factors of migration under agricultural land degradation, besides the potential consequences Consequences of the eEnvironmental cChange (Agricultural Land Degradation) as a Migration Driver.

As illustrated in Figure 3, the consequences of agricultural land degradation (ALD) on migration intention can be interpreted through the Protection Motivation Theory (PMT). Specifically, ALD increases threat appraisal by raising farmers perceived vulnerability (e.g., declining productivity, loss of income) and severity (e.g., livelihood insecurity). At the same time, migration as a coping response depends on coping appraisal, where individuals evaluate their ability to relocate successfully given available resources, skills, and networks. The interaction of these processes heightened threat appraisal alongside varying levels of coping appraisal, shapes the willingness to migrate depicted in the figure. Thus, Figure 3 integrates PMT dimensions with empirical evidence of ALD-induced migration intentions.

Formatted: Font: Bold

Formatted: Font: Bold

Formatted: English (United States)

Formatted: Justified

Formatted: English (United States)

Formatted: English (United States)

Formatted: English (United States)

2. Materials and Methods

2.1 Study Area and Sampling Strategy

The current study adopted a multi-stage approach (cluster sampling followed by systematic random household selection within each cluster), ensuring that the sample adequately represents the demographic and agricultural diversity of the selected community, making the findings generalizable to the broader rural population. We first chose Fayoum Governorate, Egypt, which is often described as a microcosm of Egypt due to its geographical, agricultural, and demographic characteristics. Fayoum, as an oasis in the desert, suffers from water shortage due to its location on the downstream branch of the Nile River (*Bahr Youssef* canal), the only water source (for agriculture and drinking)It forms a closed depression that depends mainly on the *Bahr Youssef* canal as its primary water source, which resembles the role of the Nile River for Egypt. Its waters discharge into *Lake Qarun* and *Wadi El-Rayan* areas, which resemble Egypt's Mediterranean outlets. Within Fayoum, *Itsa* district was selected as the study area for several reasons: it is the largest district in terms of both area and population (708,780 people, about 20% of the governorate's population in 2017), and many of its villages are well known as sources of migration, such as *Tatoun* (to Italy) *and Shidmu* (to France).

.

Cluster 1 (Desert Hinterland): Villages of Qalhana, Qolamsha, Qasr El-Basil, and Tatoun (population 166,878; sample size drawn = 431 > 383 minimum).

Cluster 2 (Bahr Youssef Delta): Villages of Gerdo, Matoul, and Abousir (population 197,868; sample size drawn = 488 > 384 minimum).

Cluster 3 (Bahr El-Nazla and Bahr El-Banat lands): Villages of Abogendeer and Menyet El-Heet (population 183,884; sample size drawn = 430 > 384 minimum). This cluster contains the most fertile lands.

Cluster 4 (El-Gharq El-Soltany Basin): Villages of El-Gharq, El-Hagar, and Anak (population 183,884; sample size drawn = 391 > 384 minimum).

This multi-stage approach (cluster sampling followed by systematic random household selection within each cluster) ensures that the sample adequately represents the demographic and agricultural diversity of the district, making the findings generalizable to the broader rural population of Fayoum Governorate."A random sample approach was followed to investigate 1782 respondents from the Fayoum governorate of Egypt (southern Cairo). Fayoum is one of the leading exporters of both internal and international migrants in Egypt. Many of its villages are well known as international migration

Formatted: Indent: Left: 0"

Formatted: Font: Italic

Formatted: Font: Italic

Formatted: Font: Italic

Formatted: Font: Italic

Formatted: Font: Not Italic

Formatted: Font: Italic

Formatted: Font: Italic

Formatted: Font: Italic

Formatted: Font: Italic

Formatted: Indent: Left: 0", First line: 0.35"

exporters, such as *Tatoun city* (to Italy) *and Shidmu city* (to France).~~Fayoum is one of the main exporters of both internal and international migrants in Egypt .~~ The governorate is predominantly rural, with about 26.4% of its population living below the poverty line in 2019.~~Due to the importance of Fayoum as a main exporter of internal and international migrants and the importance of Fayoum as a governorate demonised by the rural citizens (26.4%) of the Fayoum population fell below the poverty line in 2019.~~ The average percentage of the rural population in Egypt was 57.6% in 2017~~, this percentage rises~~; this percentage rose ~~-~~to 76.7% in Fayoum Governorate, and within Fayoum Governorate itself, the percentage of rural people rose to more than 90.4% among the population of *Itsa* district (CAPMAS, 2017)[5]. ~~We chose it to identify the interaction between socioeconomic factors and environmental stress as the main drivers of rural-urban migration.~~

~~Furthermore, Fayoum as an oasis in the desert suffers from water shortage due to its location on the downstream branch of Nile Revier (*Youssef Sea*), the only water source (for agriculture and drinking). Also,~~ Fayoum is considered a representative for Egypt in the water shortage issue (downstream of the Nile River Basin), where the water shortage in upstream countries will negatively affect Egypt as a country located in ~~the~~ a dry region and ~~suffer~~ suffering from groundwater limitation, doubling the problem, especially for agricultural water~~. Ministry of Planning and Economic Development, 2019, Localization Report of Sustainable Development Goals in Fayoum Governorate~~. This water shortage resulted in many issues, including high soil salinity, decreased agricultural productivity and income, and changes in the crop pattern in the last decades (to mitigate the negative impacts of this water shortage). Previously (before 2008), rice was the main crop in Fayoum (*Itsa* districts). However, after the prohibition of the cultivation of rice, the soil salinity increased in this region, and many farmers left the land or suffered from low income from agriculture as a primary income source.

For the geographical distribution of the selected sample, we chose *Itsa* district as the study area, as the last district in the *Yousef canal* branch, and it is the largest district in terms of both area and population (708,780 people, about 20% of the governorate's population in 2017) (CAPMAS, 2017)[6]. In total, 1,782 households were investigated. One adult respondent was randomly selected from each sampled household. The overall sample size was also cross-validated using Cochran's formula (1977) at a 95% confidence level and 5% margin of error [66], and adjusted for potential non-responses. To ensure representativeness, a cluster sampling strategy was adopted. The twelve local villages of *Itsa* district were divided into four geographically contiguous clusters, each forming a sub-region within

~~[5] www.capmas.gov.eg~~

[6] **CAPMAS** (Central Agency for Public Mobilization and Statistics) www.capmas.gov.eg

Formatted: Font: Italic

Formatted: Font: Italic

Formatted: Font: Not Italic

the district, as shown in **Table A.1**. Sample sizes in each cluster were calculated using Thompson's formula and cross-checked with Krejcie and Morgan's (1970) sampling tables to meet minimum requirements [65].

Formatted: Not Highlight

Formatted: Not Highlight

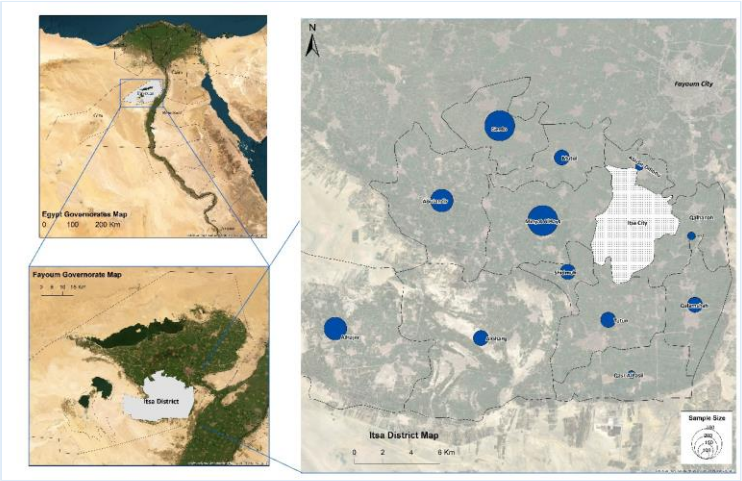

Figure 4 Study area map and distribution of the selected respondents

This water shortage resulted in many issues including high soil salinity, decreased agricultural productivity and income, and changes in the crop pattern in the last decades (to mitigate the negative impacts of this water shortage). For the geographical distribution of the selected sample, we chose *Itsa* district the most populated city in Fayoum and the last district in the *Yousef Sea* branch. Previously (before 2008), rice was the main crop in Fayoum and *Itsa* but after the prohibition of the cultivation of rice, the soil salinity increased in this region and many farmers left the land or suffered from the low income from agriculture as a main income source. Finally, we selected the respondents randomly among the twelve rural villages in *Itsa district*, as shown in **Fig. 3**. The Demographic and sample characteristics are shown in **Table A1**.

Formatted: Font: Not Italic

Formatted: Font: Italic

A structured questionnaire was developed and pretested to ensure its accuracy for achieving the study objectives, then we interviewed the respondents face-to-face duringand we interviewed the respondents face-to-face in 2023, and they self-reported the answers to the proposed questions.

2.2 Data Analysis and Empirical Approach

Many econometric models have been employed to understand the impacts of socioeconomic demographics on the likelihood of changing the place of residence and moving from rural to urban areas or immigrating abroad. In this study, the Multinomial Logistic Regression (MLR) model was employed to analyze the impact of socioeconomic demographic characteristics on the ability and willingness to migrate.

Logistic regression (LR) is used when the dependent variable is nominal (or categorical). For Binary logistic regression (two dependent variables) and multinomial logistic regression, there are more than two [67]. In LR, a logistic transformation of the odds (referred to as logit) serves as the dependent variable [67,68]:

$$\log(odds) = \text{logit}\ \ (\text{P}) = \ln(\frac{P}{1-P}) = a + \beta_1 x_1 + \beta_2 x_2 + \beta_3 x_3 \ldots\ \ \beta_\text{n} x_\text{n} \qquad (1)$$

Or

$$\text{P} = \frac{exp(a+\beta_1 x_1+\beta_2 x_2+\beta_3 x_3 \ldots\ldots)}{1+exp(a+\beta_1 x_1+\beta_2 x_2+\beta_3 x_3 \ldots\ldots)} \qquad (2)$$

Where:

p = the probability that a case is in a particular category,

exp = the exponential (approx. 2.72),

a = the constant of the equation and,~~,~~

b = the coefficient of the predictor or independent variables.

Logits or Log Odds

Odds value ranges from zero to infinity and tells us "how much more likely it is that an observation is a member of the target group rather than a member of the other group" [53].

Odds = p/(1-p)

The odds ratio (OR), estimates the change in the odds of membership in the target group for a one-unit increase in the predictor [67]. It is calculated by using the regression coefficient of the predictor as the exponent or exp.

In our case, the Y (dependent variable) is the desire to change the place of residence and leave the village (Migrate), where "0" is "No~~ I don't want" and "1" is "Maybe I want" and "2" is "Yes~~, I don't want", "1" is "Maybe I want", and "2" is "Yes, I want". The explanatory variables include demographic ~~and socioeconomic~~ factors, economic factors, and agricultural factors, as presented in **Table**

~~A1~~A2. For the perceptions of change based on available data that include (age, family size, agriculture share of income, gender, marital status, educational status, monthly income, income adequacy, working in the agricultural sector, ownership of agricultural land, changes in land ownership during the last 10 years, changes in the farming land during the previous 10 years). The rice crop cultivation, beet crop cultivation, changes in land productivity, living conditions of the farmer now compared to 10 years ago, and current village conditions compared to 10 years ago) have been considered.

The reference category for gender is "Males", marital Status is "Married", educational Status is "high-school", and monthly income is "from 2000 to 4000 LE.[7]", income adequacy is "Somewhat enough", working in the agricultural sector is "Yes", ownership of agricultural land is "Yes", Changes in land ownership during the last 10 years is "Area has not changed", Changes in the farming land (cultivated area) during the last 10 years is "Area has not changed", Rice crop cultivation is "No cultivation", Beet crop cultivation is "No cultivation", Changes in land productivity is "Now better", Living conditions of the farmer now compared to 10 years ago "Now better", and Village conditions now compared to 10 years ago is "Now better".

Finally, we used the weighted percentage to rank the motivations and barriers of migration under the ALD as follows~~;~~: Weighted percentage % = $\frac{\text{Total marks}}{N*3}$ *100, where N represents the sample size. We used the same model to assess the impact of socioeconomic characteristics of respondents on motivation and barriers to migration intention through a composite variable.

3. Results and discussion

The following analysis is guided by the Protection Motivation Theory (PMT), which frames migration intention as an outcome of both threat appraisal and coping appraisal. In this study, threat appraisal is reflected in how farmers perceive the severity and vulnerability associated with agricultural land degradation, while coping appraisal is captured by their resources, education, and access to alternative livelihood strategies. Accordingly, each set of results is interpreted in relation to these theoretical dimensions to ensure consistency between the framework and empirical findings.

Environmental changes, such as land degradation, climate change, and natural disasters, tend to encourage rural populations to migrate to urban areas [1,46]. In the following section, we will discuss the main results, including the interaction between socioeconomic factors and environmental stress as drivers for migration.

[7] 1 $USA = 30.743 LE (Egyptian pound as an average 2023-) based on the Central Bank of Egypt data. https://www.cbe.org.eg/en

Formatted: MDPI_3.1_text, Indent: First line: 0.35", Line spacing: 1.5 lines

Field Code Changed

3.3. Model Fitting and Pseudo R-Square

The multinomial logistic regression model was evaluated using several key metrics, beginning with the model fitting information. The final model had a significantly lower -2 Log Likelihood value (3272.3) compared to the intercept-only model (3772.3), indicating a better fit of the full model. The Chi-Square statistic for the model was 499.98 with 80 degrees of freedom, which is statistically significant ($p < 0.001$). This suggests that the model as a whole is a good fit for the data.

Pseudo R-square values, including Cox and Snell (0.245), Nagelkerke (0.278), and McFadden (0.132), further indicate the model's explanatory power. While these values do not have the same interpretation as R-squared in linear regression, they do provide a measure of the proportion of variance explained by the model. In this case, the Nagelkerke R-square value of 0.278 suggests that the model explains about 27.8% of the variance in the dependent variable, which is moderately strong for social science data.

3.1. Descriptive statistics

The respondents' socioeconomic characteristics have been presented in **Tables A12, and A2A3,** where more than two-thirds of the respondents (83%) were males and married (68.9%), and most of them were educated (80%). However, more than 40% of them don't own agricultural land, and around one-quarter (26%) of them depend on agriculture as a primary income source. The findings from the descriptive statistics reveal significant trends regarding land ownership and farming areas. Nearly half of the respondents (46.3%) reported no change in land ownership over the past decade, while 53.3% indicated stability in the area they currently cultivate. This reflects limited land market activity and the role of inheritance in maintaining land ownership structures. This is consistent with Deininger and Jin, who noted that land tenure systems in developing countries often restrict land redistribution, leading to long-term stability in ownership [69].

Regarding crop structure, rice remains a dominant crop, with 60.4% of respondents cultivating it only within the past 10 years, highlighting its importance in local agricultural systems in the investigated area. This finding highlights the difficulty of changing the cropping system, which aligns with Elmpghazy and Elshenawy, who reported that rice is a staple in rural Egypt due to its profitability and the availability long farmers' experiencesof irrigation, and they consider it a main crop in most of the northern Egyptian governorates [39]. For sugar beet cultivation, the distribution is more balanced, with

Formatted: Not Highlight

36.9% cultivating beet both currently and a decade ago, suggesting the role of crop rotation and adaptation to market demands [3]**.**

A concerning trend is the decline in land productivity, as reported by 35.8% of respondents. This decrease may be attributed to factors such as soil degradation, water shortages, and climate change. Mahmoud (2019) found similar patterns in Egypt, where environmental stressors have contributed to declining agricultural productivity and, consequently, lower farm incomes and increased migration rates [34]. ~~This is further exacerbated by economic challenges~~Economic challenges further exacerbate this, as the study shows that 43.4% of respondents earn between 2000 and 4000 EGP (less than $80) per month. This range~~per month, a range that~~ limits financial stability and motivates people to migrate in search of a better life for their families. Moreover, only 26.6% of respondents considered their income sufficient, reinforcing findings from Radwan et al. (2022), who highlighted persistent rural poverty in Egypt due to limited off-farm employment opportunities [70].

The study also captures perceptions of agricultural production and living conditions. A significant proportion of respondents (44.2%) believe that agricultural production has degraded over the past decade. Zohry (2005) attributes this decline to increased production costs, limited access to quality inputs, and environmental challenges. Similarly, more than half (53.4%) of respondents specified that living conditions have worsened in the last ten years due to the higher living costs and a decrease in the sufficiency of income [30]. This trend is supported by Abdelwahed et al. (2020), who found that rural communities in Egypt have struggled due to inflation and economic reforms that have not equally benefited all social classes [29]. This highlights the significant role of social protection and women's empowerment in reducing the streams of migration from the vulnerable areas.

Migration trends show that 45.2% of respondents do not wish to leave their community, while 22.4% actively seek relocation, which will be discussed in the motivation and barriers section later. Among those willing to migrate, Greater Cairo (40.2%) and destinations outside Egypt (29.4%) were the most desired. These patterns reflect broader economic migration trends, where rural populations move to urban centers or abroad in search of better opportunities for their families [30]**.** The ~~main~~ primary motivations for migration were access to suitable job opportunities (70.1%) and higher wages (66.8%), which is consistent with findings from Abdelwahed et al. (2020), who emphasized that economic factors are the strongest drivers of rural-to-urban migration in Egypt [29]. This highlights the importance of empowering farmers to utilize advanced technology, thereby enhancing their income and reducing migration streams from vulnerable rural areas to the main cities or abroad.

Despite the desire to migrate, several barriers exist, with t~~. T~~he most cited obstacles ~~were~~ being the difficulty of moving (54.1%) and the high cost of relocation (52.7%). Our findings align with those

of Abdelwahed et al. (2020), who found that limited financial resources and the lack of affordable housing often deter rural residents from moving. Similarly, inadequate services in rural areas also influence migration decisions, with 48.5% of respondents dissatisfied with both educational and healthcare services [29]. These findings align with Zohry (2005), who reported that rural Egypt faces severe challenges in access to quality education and healthcare due to underinvestment in infrastructure [30]. This underscores the essential need for equitable economic development across the regions in reducing the pressure of migration, and gives priority to the vulnerable areas for public investment in education, health, and infrastructure.

3.2. Determinants of migration intention –

Formatted: MDPI_2.1_heading1

The results of the migration determinants that play a significant role in motivating or inhibiting people to migrate from one region to another have been presented in ~~**Table 1** and **Table**~~ **Tables 1 and 2**. The interrelationship between these variables and willingness to ~~migrate~~relocate, which ~~represented as (yes~~is represented as (yes, I will ~~reallocate~~relocate or maybe). This includes the demographic, economic, and farming systems of the selected respondents, presented as follows;

3.2.1 Model Fitting and Pseudo R-Square

Formatted: MDPI_2.3_heading3

The multinomial logistic regression model was evaluated using several key metrics, beginning with the model fitting information. The final model had a significantly lower -2 Log Likelihood value (3272.3) compared to the intercept-only model (3772.3), indicating a better fit of the whole model. The Chi-Square statistic for the model was 499.98 with 80 degrees of freedom, which is statistically significant ($p < 0.001$). This suggests that the model as a whole is a good fit for the data.

Pseudo R-square values, including Cox and Snell (0.245), Nagelkerke (0.278), and McFadden (0.132), further indicate the model's explanatory power. While these values do not have the same interpretation as R-squared in linear regression, they do provide a measure of the proportion of variance explained by the model. In this case, the Nagelkerke R-square value of 0.278 suggests that the model explains about 27.8% of the variance in the dependent variable, which is moderately strong for social science data.

3.~~2~~2.~~1~~2. Demographic Factors

Age: The results show that age is a significant predictor ($p < 0.001$) across all categories, with an odds ratio (Exp(B)) less than 1 (0.944) for "maybe" and 0.951 for "yes". This suggests that with increasing age, the respondent's willingness to migrate and leave the village will ~~be decreased~~decrease.

This is consistent with previous studies' findings that younger individuals were more inclined to migrate compared with older populations under the same ~~different~~ risk sources. A recent study confirmed that young Egyptian people (18-29 years) are more motivated to migrate based on their age, marital status, and employment status [29]. Although the employment status was insignificant as a migration driver after the Arab Spring of 2011, compared with before it (2008), the remaining factors still significantly drive internal and international migration [29]. For instance, especially in rural areas, older adults are generally more inclined to stay in their current residences due to established social networks and greater attachment to their communities [29]. Furthermore, the youth could be more aware of the negative impacts of climate change, which increases their willingness to move from the most affected regions (rural) and sectors (agriculture) to other service sectors in the cities [52]. Based on the PMT assumptions, our results confirm that the youth are more motivated to relocate due to their higher perceived severity and vulnerability, which increases their motivation to relocate, and lower restrictions to delay relocation, compared to elderly people with families. However, the willingness to migrate will be lower under the government aid, social support, and attachment to one's homeland, which should be considered for future intervention.

~~**Family Size**: Family size also emerged as a significant predictor for the "maybe" category (p = 0.016, Exp(B) = 1.078), suggesting that larger families are more likely to consider moving as a whole or send some family members to capital cities or to abroad as well to secure more income for the family members, which consistency with Abdelwahed et al. (2020).~~

Table 1: Overall effect of the main variables on the migration intention

Effect	Model Fitting Criteria<br>-2 Log Likelihood of Reduced Model	Likelihood Ratio Tests<br>Chi-Square	df
Intercept	3272.342ᵃ		
Age	3389.866	117.523***	2
Family Size	3278.914	6.572**	2
Agricultural share of total income	3276.911	4.569	2
Gender	3290.362	18.020***	2
Marital Status	3277.869	5.527	6
Educational Status	3294.111	21.769**	10
Working in the agricultural sector	3277.028	4.686*	2
Agricultural land ownership	3273.606	1.264	2

Formatted: Font: Not Bold

Formatted: Font: Not Bold

Formatted Table

Changes in Agricultural land ownership in last 10 years	3274.118	1.776	6
Changes in total cultivated area in the past 10 years	3276.300	3.958	6
Monthly Income	3315.200	42.858***	6
Income adequacy	3289.551	17.209**	4
Changes in land productivity	3281.266	8.924*	4
Living conditions of the farmer now (compared to 10 years ago)	3280.092	7.750	4
Changes of village conditions now compared to 10 years ago	3275.188	2.846	4

Model Fitting Information

Model	Model Fitting Criteria	Likelihood Ratio Tests	
	-2 Log Likelihood	Chi-Square	df
Intercept only	3772.331		
Final	3272.342	499.989**	80

Pseudo R-Square

Cox and Snell	0.245
Nagelkerke	0.278
McFadden	0.132

The chi-square statistic is the difference in -2 log-likelihoods between the final model and a reduced model. The reduced model is formed by omitting an effect from the final model. The null hypothesis is that all parameters of that effect are 0.

a. This reduced model is equivalent to the final model because omitting the effect does not increase the degrees of freedom.

*** Values are significant at P = 0.01, ** Values are significant at P = 0.05, * Values are significant at P = 0.1

Family Size also emerged as a significant predictor for the "maybe" category (p = 0.016, Exp(B) = 1.078), suggesting that larger families are more likely to consider moving as a whole or send some family members to capital cities or to abroad as well to secure more income for the family members, which consistency with Abdelwahed et al. (2020) [29]. This could be because larger families might seek better living conditions or more opportunities, which aligns with previous research highlighting the impact of family size on migration decisions, such as the study by Mulder and Cooke (2009), who observed that households with more members may seek relocation to improve living conditions or access better services [71]. Due to the nature of social networks in Fayoum as a tribal society, most families prefer to have more children, especially males who will immigrate to EU countries later (it is considered a social superiority in this society). Larger families might also relocate to urban areas to

Formatted Table

Formatted: Indent: Left: 0"

access better educational opportunities for their children, a common trend observed in rural-to-urban migration studies. At the same time, changes have occurred in the crop pattern in Fayoum, which could be another reason. The previous crop rotation, which included rice and cotton, was able to observe a labour supply surplus due to its need for intensive human labour in farm operations and harvesting. The fragmentation of agricultural land (under the limitation of reclaiming new lands) has another impact on reducing per capita land share and decreasing the rural income due to the land and water limitations in the study area, where the literature revealed that the fragmentation of agricultural land decreases the profitability of farms in the rural areas and leads to migration [72].

Gender significantly affects the decision to move, with males being less likely to want to change residence ($p < 0.001$, Exp (B) = 0.521 for "maybe" and 0.495 for "yes"). This gender difference is consistent with the literature that suggests men often have stronger ties to land and agriculture, making them less inclined to migrate. This supports findings from studies like those by Mulder and Cooke (2009) [71], who noted that women, particularly in patriarchal societies, might seek to relocate for better employment opportunities and autonomy. This pattern reflects broader gender dynamics where women might perceive urban areas as offering more freedom and opportunities. However, given the nature of Egyptian social norms, it would be expected that women have a low willingness to migrate, but due to globalization and access to ICT, the new generations of women are more willing to migrate compared to men [58]. This could go back to the land ownership in the rural areas in Egypt, mostly registered for male householders rather than women, which increased the women's desire for more empowerment that could be more available in the urban regions. Furthermore, the gender differences observed are significant, highlighting the ongoing gender disparities in rural areas where men might have more opportunities locally. At the same time, women perceive urban areas as avenues for greater autonomy and employment. This is consistent with the literature findings that revealed that rural women often migrate to escape restrictive social norms and pursue independent livelihoods [73]. On the other hand, the women who are looking for more women's empowerment prefer to leave the village to be able to find good opportunities for better education, employment, and a mirage in the capital cities [74].

Formatted: Font: Not Bold

Educational Status: Education level was significant for both categories (yes, maybe), particularly for higher education levels ($p < 0.001$ for some categories). Individuals with higher **education** were more likely to consider moving (Exp(B) = 1.760 for "maybe" and 2.089 for "yes"). This finding supports previous studies that have demonstrated that education increases awareness of opportunities outside rural areas and thus increases the likelihood of migration [74,75]. For example, it is noted that

education significantly predicts migration intentions, particularly among younger adults seeking professional development [75]. The recent literature revealed that education has a significant impact on motivating young people to migrate, ; at the same time, those people who have already migrated will invest more to support their families and receive a good education [33,76]. A recent study confirmed that young Egyptian laborers have more opportunities in Arab countries when they have a higher education degree than uneducated people [29]. At the same vein, the high skilled and more educated people are ready to immigrate especially if they In the same vein, highly skilled and educated individuals are more likely to immigrate, particularly if they have already relocated from their birth village (internally migrated) to pursuealready left their birth village (internally migrated) to receive their education in the city or abroad.

The Our findings,findings indicate that demographic and social factors, particularly age, family size, gender, and education, play a crucial role in determining the likelihood of wanting to relocate. Based on the PMT assumptions, our The results suggest that older individuals are more inclined to remain in the village, possibly due to stronger ties to the community or a reduced propensity for change. In contrast, higher educational attainment and greater income levels increase the likelihood of wanting to leave, possibly reflecting aspirations for better opportunities elsewhere [46,77]. These results align with the broader literature on migration and residence decisions, particularly in rural contexts [77]. The significant role of the combination (age, family size, gender, and education) reflects well-documented trends in the migration literature. For instance, younger individuals and those with higher education levels are typically more mobile, seeking better opportunities in urban areas [29,74]. Our results confirm the PMT assumptions, indicating that higher perceived severity and vulnerability increase motivation to relocate, while lower restrictions to delay relocation are observed compared to elderly people with families. Nevertheless, the inclination to migrate is likely to decrease due to government assistance, social support, and a strong connection to one's homeland, all of which should be taken into account for future interventions.

Formatted: Font: Not Bold, (Asian) Chinese (PRC)

3.2.23. Economic Factors

Income and Income Adequacy: Both Both monthly income and perceived income adequacy were significant predictors. Interestingly, lower income was associated with a higher likelihood of wanting to leave the village, while perceptions of income adequacy showed a complex relationship, which is consistent with [78]. Lower income groups were more likely to want to move ($p < 0.001$,

Exp(B) = 0.573 for "maybe" and Exp(B) = 1.735 for "yes"). The significant role of income in migration decisions aligns with economic theories of migration and PMT assumptions, such as those proposed by [79], which posit that individuals are more likely to move when they perceive better economic opportunities elsewhere. However, the complex relationship with income adequacy reflects the findings of studies like that by [78], which ~~suggest~~ suggests that subjective perceptions of economic well-being can be as ~~important~~ crucial as actual income in migration decisions. Furthermore, the political economy of EU-Egypt migration has been discussed to stop the irregular flows to the EU countries through the Mediterranean Sea for the people who want to secure high-wage jobs that aren't available in their domestic (Egypt) labour market [23,80]. These studies confirmed that the Egyptian labor market can export skilled labor to the EU or other Arab countries by establishing training centers to enhance the skills of those people willing to migrate.

3.2.~~3~~4. Agricultural System Factors

Agricultural Engagement: Working in the agricultural sector was negatively associated with the desire to move from villages (for the land owners), though the association was not always significant (p = 0.053). This could indicate a strong attachment to agricultural livelihoods among those employed in this sector. These findings are consistent with the study of Gray et al., who found that individuals engaged in agriculture were less likely to migrate, as they were more attached to their land and agricultural activities [9]. However, we observed that the young rural ~~in the selected area were mostly willing to migrate due to the negative impacts of climate change and environmental stress on the agricultural sector~~people in the selected area were mainly willing to migrate due to the negative impacts of climate change and environmental stress on the agricultural sector, which puts this sector at high risk. Another study has discussed how households with strong agricultural livelihoods often exhibit lower migration rates, particularly in contexts where agriculture remains a viable livelihood strategy [81]. Most of the farmers in the selected area depend on agricultural production as their ~~main~~ primary income source, where the shortage of water increases the salinity of the soil ~~decreasing the productivity and income of those farmers and forcing them to migrate (in case they don't own land or their ownership is~~ , decreasing the productivity and income of those farmers and forcing them to migrate (in case they don't own land or their ownership is a small area).

Changes in land productivity or farming systems due to climate change and other socioeconomic factors push many people to migrate, prompting stakeholders to seek different employment opportunities in urban areas. This encourages rural-urban migration~~,~~ ; this finding is consistent with the recent literature [41,52]. The recent literature revealed that rural people in Africa will face a decline

in agricultural production due to the negative impacts of climate change and land degradation, and they ~~are they~~ will be the most affected and exposed to migration [82], especially in vulnerable areas like the study area (Fayoum). Unfortunately, most of the young people in the selected area ~~either are~~are either willing to migrate to the EU countries through illegal migration or move (temporarily or permanently) to find work in capital cities like Cairo or Alexandria, which decreases the labour supply during the seasonal demand for agricultural labour ~~demand~~ in this area. Salinity reduces soil fertility in the selected area, thereby ~~decreasing~~ lowering crop yields and forcing the farmers to change the cropping pattern, which finally decreases the agricultural income~~, this~~. This degradation compels farmers to abandon their land and seek better opportunities in urban areas [27]. ~~These~~ Our findings align with recent research, which found that desertification in the Inner Mongolia (Northern China) region has resulted in large-scale rural displacement[20]. Also, in Bangladesh, riverbank erosion and soil salinity in coastal areas have forced many rural inhabitants to migrate to urban slums in Dhaka, highlighting the link between environmental degradation and urban poverty [16,22]. This highlights that increasing soil salinity leads to decreased productivity and income, and subsequently an increase in migration willingness in the future. The intervention should focus more on the technologies and farming practices that enhance the soil fertility under the water shortage and soil salinity to reduce the stream of migration in future interventions.

3.2.~~4~~5. Perceptions of Change

Perception of Living Conditions: Individuals who believed that living conditions for farmers had improved over the last decade were less likely to consider moving ($p = 0.033$). This highlights the importance of perceived improvements in quality of life in retaining rural populations. This finding supports previous studies that have demonstrated how perceived improvements in local living conditions, particularly in rural areas, can reduce the likelihood of migration by enhancing satisfaction with local opportunities and quality of life [83]. Migration decision depends on the interaction of a set of variables~~ including migrants' socioeconomic characteristics and environmental change, that should be considered during the designing~~, including migrants' socioeconomic characteristics and environmental change, that should be considered during the design of the intervention to tackle ~~the~~ migration. The real example, in this case, is the national initiative in China (rural revitalization), which aims to support rural communities to decrease rural-urban migration and secure agrofood products for local self-sufficiency [83].

Although some variables included in the current study have been statistically insignificant e.g~~ (~~, marital status and ownership of agricultural land~~)~~, ~~but~~ these factors are still influential in understanding

and explaining the migration intention and decision, which have been confirmed in the literature [25,50,51]. ~~So future studies could investigate deeply~~Future studies could therefore investigate the interaction between these sets of variables in different regions ~~to better understand migration motivations~~to understand migration motivations better.

3.3. Model Fitting and Pseudo R-Square

The multinomial logistic regression model was evaluated using several key metrics, beginning with the model fitting information. The final model had a significantly lower -2 Log Likelihood value (3272.3) compared to the intercept-only model (3772.3), indicating a better fit of the full model. The Chi-Square statistic for the model was 499.98 with 80 degrees of freedom, which is statistically significant ($p < 0.001$). This suggests that the model as a whole is a good fit for the data.

Table 2 the The willingness of the respondents to migrate from their current place (results of the multinomial regression models)

Parameter Estimates	(1) Maybe			
	(1) Maybe		(2) Yes	
**Do you want to change your place of residence and leave the village? (Maybe or yes)**	Std. Error	Exp(B)	Std. Error	Exp(B)
Intercept	0.366		0.420	
Age	0.006	0.944**	0.007	0.951***
Family Size	0.032	1.078*	0.035	1.068**
Agriculture share of income	0.218	0..662	0.006	1.018
[Gender=1] Females	0.185	0.521***	0.194	0.495***
[Educational Status=1] Illiterate	0.191	1.038	0.219	0.753
[Educational Status=2] Reads and writes	0.186	1.161	0.219	0.918
[Educational Status=3] Basic education	0.280	1.072	0.331	0.900
[Educational Status=4] University and post-university	0.200	1.760**	0.213	2.089**
[Educational Status=5] Institute (above intermediate)	0.261	1.681*	0.314	1.240
[Do you work in the agricultural sector?=0] No	0.166	0.725*		
[Income (monthly)=1] (less than 2000)	0.173	0.573***	0.173	1.735**
[Income (monthly)=2] (4000- 6000)	0.156	0.826	0.193	0.783
[Income (monthly)=3] (more than 6000)	0.206	0.828	0.231	1.013
[Income adequacy=1] Not enough	0.144	1.014		
[Income adequacy=2] Enough	0.155	0.597***		
Living conditions of the farmer now compared to 10 years ago= (Now worse)	0.171	1.439**		
Living conditions of the farmer now compared to 10 years ago= (As it was 10 years ago)	0.200	1.083		
Changes in land productivity =2] As it was 10 years ago			0.226	0.559**

Formatted Table

Formatted: Centered

Formatted: Font: Bold

*** Values are significant at P = 0.01, *, ** Values are significant at P = 0.05, * Values are significant at P = 0.1

~~**Pseudo R-square values, including Cox and Snell (0.245), Nagelkerke (0.278), and McFadden (0.132), further indicate the model's explanatory power. While these values do not have the same interpretation as R-squared in linear regression, they do provide a measure of the proportion of variance explained by the model. In this case, the Nagelkerke R-square value of 0.278 suggests that the model explains about 27.8% of the variance in the dependent variable, which is moderately strong for social science data.**~~

Formatted: MDPI_2.1_heading1, Indent: First line: 0"

3.~~3~~2.5. Motivations and Barriers of Migration Intention

Formatted: MDPI_2.1_heading1

~~A~~3.3.1 Motivations of Migration

The primary driver of migration is the availability of suitable job opportunities in another governorate, with 70.1% of respondents agreeing on this factor, ranking it first with a weighted percentage of 84.3%,, as shown in ~~table~~ Table 3. This result aligns with Radwan et al. (2022), who found that the lack of job opportunities in rural areas significantly pushes internal migration in Egypt [84]. Similarly, the pursuit of higher wages or salary increases ranked second (66.8%) with a weighted percentage of 83.0%, confirming that economic incentives play a crucial role in migration decisions [29].

The possibility of starting a project with a higher income in destination areas ranked third (62.4%) with a weighted percentage of 81.3%, reflecting the aspiration for better income-generating activities. This finding aligns with Abdelwahed et al. (2020), who reported that rural populations often seek self-employment opportunities as an alternative to low agricultural incomes [29]. Furthermore, the inadequacy of current income (52.9%) emerged as another significant driver with a weighted percentage of 77.3%, consistent with Xu et al. (2020), who highlighted that low-income levels are one of the primary factors motivating rural-to-urban migration [21].

The quality of public services also affects migration decisions. Poor educational services (48.5%) and health services (48.5%) in villages ranked among the top reasons, with weighted percentages of 75.0% and 75.3%, respectively. These results are supported by Selod et al. (2021), who found that inadequate infrastructure and services in rural regions push many families to seek better living conditions in urban areas [52].

Table 3: Weighted percentage for the migration motivations under land degradation

Why do you want to change your current accommodation place?	Scale			Total marks	Weighted percentage%	Rank
	agree	Neutral	Disagree			

Formatted Table

	F	%	F	%	F	%			
To find a suitable job opportunity in another governorate	1249	70.1	220	12.3	313	17.6	4500	84.3%	1
To transfer my job to another governorate	893	50.1	425	23.8	464	26	3993	74.7%	8
To find a higher wage or a salary increase there	1191	66.8	271	15.2	320	18	4435	83.0%	2
To open a project with a higher income there	1112	62.4	346	19.4	324	18.2	4352	81.3%	3
The cost of living here is expensive	691	38.8	523	29.3	568	31.9	3687	69.0%	12
I can’t find a job in my village	857	48.1	434	24.4	491	27.6	3930	73.7%	11
My salary or monthly income in the village is not enough for me	943	52.9	472	26.5	367	20.6	4140	77.3%	4
I opened a project in the village, and its income is not good	633	35.5	461	25.9	688	38.6	3509	65.7%	13
I don’t know how to work more than one job in my village	859	48.2	456	25.6	467	26.2	3956	74.0%	10
To find clean housing with reasonable prices in another governorate	876	49.2	436	24.5	470	26.4	3970	74.3%	9
The educational services in the village are not good	865	48.5	493	27.7	424	23.8	4005	75.0%	7
The health services in the village are not good	864	48.5	514	28.8	404	22.7	4024	75.3%	6
The entertainment services in the village are not good	937	52.6	438	24.6	407	22.8	4094	76.7%	5

Weighted percentage% = $\frac{\text{Total marks}}{N*3}$ $\frac{\text{Total marks}}{N*3}$ *100, where the sample size is (N=1782)

3~~B~~.3.2 Barriers ~~Obstacles~~ to Migration

Despite the desire to migrate, several obstacles prevent rural residents from moving, as shown in **Table 4**. The most significant barrier was the difficulty of moving to a new place, reported by 54.1% of respondents, with a weighted percentage of 78.0%. This finding aligns with Selod et al. (2021) observation that rural households face logistical and financial challenges when relocating [52].

Family objection (54.2%) and high moving costs (52.7%) were the next most prominent obstacles, both ranked third with a weighted percentage of 76.3%. These results are consistent with Zohry (2005), who found that family ties and financial constraints are key barriers to migration in rural Egypt [30].

Other significant obstacles included the lack of suitable housing (51.6%) and the fear of social isolation (51.4%), with weighted percentages of 76.7% and 76.0%, respectively. These findings align with Xiang et al. (2016), who reported that rural migrants often struggle to integrate into new communities due to the lack of social networks and affordable housing options [57].

~~Overall, the analysis of migration intentions in Fayoum can be clearly understood within the Protection Motivation Theory (PMT) framework. Agricultural land degradation significantly increases threat appraisal by raising farmers perceived severity and vulnerability, as declining productivity and livelihood insecurity heighten the risk of staying. At the same time, socio-economic resources such as education, remittances, and land assets serve as coping appraisal factors, shaping households' capacity to adapt locally or migrate successfully. The identified drivers of migration—such as environmental stress and unemployment—correspond to dimensions of threat appraisal, while the barriers—including limited resources, family obligations, and lack of skills—reflect coping constraints. Taken together, these findings demonstrate that migration decisions emerge from the interaction between threat and coping appraisals. This provides an integrated explanation of why some households choose to migrate while others remain, underscoring the value of PMT as a guiding theoretical framework for analyzing environmentally induced migration in developing-country contexts.~~

Formatted: Font: Times New Roman

Formatted: Font: 10 pt

Formatted: Font: 10 pt

Formatted: Indent: Left: 0", First line: 0"

Formatted: MDPI_2.3_heading3

Formatted: Font: Not Italic

Formatted: Font: Not Italic

Formatted: Highlight

Formatted: Highlight

Table 4 Weighted percentage for the migration barriers under land degradation

What might be an obstacle for you to leave your village and work or live in another province?	Scale						Total marks	Weighted per-centage%	Rank
	agree		Neutral		Disagree				
	F	%	F	%	F	%			
Difficulty of moving to the new place	964	54.1	452	25.4	366	20.5	4162	78.0%	1
Lack of suitable housing in the new place	919	51.6	482	27	381	21.4	4102	76.7%	2
Cost of moving	940	52.7	425	23.8	417	23.4	4087	76.4%	3
Family objection	966	54.2	360	20.2	456	25.6	4074	76.2%	4
I might feel isolated and alone in the new place	916	51.4	447	25.1	419	23.5	4061	76.0%	5
Lack of friends and relatives in the new place	896	50.3	465	26.1	421	23.6	4039	75.7%	6
Distance of travel	831	46.6	517	29	434	24.4	3961	74.0%	7
Lack of continuous transportation to the new place	827	46.4	498	27.9	457	25.6	3934	73.7%	8

3.3.3 Motivations and Barriers Behind Migration Decisions

We estimated the impact of the demographic and economic characteristics of the respondents on their willingness to relocate based on the assumptions of PMT, highlighting the motivations and barriers behind migration decisions. Therefore, we constructed a composite variable for the motivation and another for the barriers with three levels (high, medium, and low); we considered the medium as a reference in our estimations. Through this variable, we estimated the perceived intention of respondents to relocate as a result of the environmental change and low agricultural income in the selected area, based on their socioeconomic characteristics. The determinants that influence whether individuals are motivated to migrate or face barriers to relocation are summarized in **Tables 5 and 6**. The results reveal that both demographic and economic characteristics significantly affect migration intentions, although the magnitude and direction of these effects vary between the motivation and barrier models.

Model Fit

Formatted Table

Formatted: Default Paragraph Font, Font: Times New Roman, 12 pt

Formatted: Font: (Default) Times New Roman, 12 pt

Formatted: Normal, Indent: First line: 0.35"

Formatted: Font: (Default) Times New Roman, 12 pt

Formatted: Font: (Default) Times New Roman, 12 pt

Formatted: Font: 12 pt, Bold

Formatted: Font: 12 pt

Formatted: Default Paragraph Font, Font: Times New Roman, 12 pt, Font color: Auto

Formatted: Font: 12 pt, Font color: Auto

Formatted: Line spacing: 1.5 lines

Both models demonstrated acceptable goodness-of-fit. The Motivation model reported −2 Log Likelihood = 1845.31 and Chi-Square = 191.46 (df = 62, $p < 0.001$), with Pseudo R^2 values of Cox & Snell = 0.167, Nagelkerke = 0.195, and McFadden = 0.094. The Obstacles model yielded −2 Log Likelihood = 1935.88 and Chi-Square = 129.97 (df = 62, $p < 0.001$), with Pseudo R^2 values of Cox & Snell = 0.117, Nagelkerke = 0.136, and McFadden = 0.063. These results indicate a moderate explanatory capacity, appropriate for social-science datasets.

Demographic Factors, Age emerged as a significant predictor of migration intention in both models. In the *Motivation* model, the odds ratio (Exp(B) = 1.046, $p < 0.01$) indicates that as respondents grow older, their likelihood of considering migration slightly increases. Similarly, in the *Obstacles* model, Exp(B) = 1.023 ($p < 0.05$) shows that older individuals perceive fewer barriers to migration. These results contrast with earlier evidence suggesting that younger people are generally more mobile and willing to migrate for better opportunities. At the same time, older individuals tend to remain in their communities because of established livelihoods and social networks [29,71]. However, in rural Egypt, age can also reflect financial independence and accumulated capital, which may facilitate migration rather than deter it. Gender significantly influenced migration obstacles. In the *Obstacles* model, females were more likely to experience migration motivation (Exp(B) = 1.589, $p < 0.10$). This pattern aligns with studies highlighting that women in rural areas often view migration as a pathway to greater autonomy and improved employment opportunities [85,86]. Although men usually maintain stronger ties to land ownership and agricultural work, limited local opportunities for women often drive them toward urban or overseas migration [87]. These findings emphasize that gendered access to resources and employment remains a central determinant of migration behavior in rural Egypt.

Education was a significant determinant across several categories in the *Obstacles* model. Respondents with university or post-university education exhibited a lower Exp(B) = 0.424 ($p < 0.01$), while those with intermediate-level qualifications (institutes) recorded Exp(B) = 0.349 ($p < 0.01$). Conversely, illiterate respondents showed Exp(B) = 0.497 ($p < 0.05$), implying that lower education levels are associated with more substantial obstacles to migration. These results indicate that higher educational attainment facilitates migration decisions, consistent with findings that education enhances

Formatted: Font: 12 pt
Formatted: Font: 12 pt, Not Italic
Formatted: Font: 12 pt
Formatted: Indent: First line: 0.35", Line spacing: 1.5 lines
Formatted: Font: 12 pt, Not Italic
Formatted: Font: 12 pt
Formatted: Font: 12 pt, Not Bold, Font color: Auto
Formatted: Font: 12 pt, Font color: Auto
Formatted: Font: 12 pt
Formatted: Font: 12 pt
Formatted: Font: 12 pt
Formatted: Font: Not Bold
Formatted: Font: 12 pt
Formatted: Font: 12 pt
Formatted: Font: 12 pt
Formatted: Font: 12 pt, Bold, Font color: Red
Formatted: Font: 12 pt
Formatted: Font: 12 pt

awareness of opportunities and mobility [85]. Similar trends have been reported among Egyptian youth, where educated individuals pursue migration for professional development or improved income prospects [29].

Table 5. Overall Effect of the Main Variables on Migration Intention (Motivation and Barriers)

Effect	Motivation			Barriers		
	Model Fitting Criteria (-2 Log Likelihood of Reduced Model)	Chi-Square	df	Model Fitting Criteria (-2 Log Likelihood of Reduced Model)	Chi-Square	df
Intercept	1845.308[a]	0.000		1935.884[a]	0.000	
Age	1873.284	27.976***	2	1935.884[a]*	0.000	2
Gender	1854.039	8.732**	2			
Educational Status	1865.923	20.615**	10			
Job	1863.314	18.006**	8			
Income Adequacy	1867.576	22.268***	4			
Monthly income				1944.038**	8.154	2
Marital Status				1950.504**	14.620	6
Change in land productivity.				1951.118***	15.234	4
Change in village conditions now compared to 10 years ago.				1944.920*	9.036	4
Model Fitting (-2 Log Likelihood)	1845.31	191.46***	62	1935.88	129.97***	62
**Pseudo R-Square**						
Cox and Snell		0.167			0.117	
Nagelkerke		0.195			0.136	
McFadden		0.094			0.063	

*** Significant at $p < 0.01$ ** Significant at $p < 0.05$ * Significant at $p < 0.10$

The chi-square statistic is the difference in -2 log-likelihoods between the final model and a reduced model. The reduced model is formed by omitting an effect from the final model. The null hypothesis is that all parameters of that effect are 0.

a. This reduced model is equivalent to the final model because omitting the effect does not increase the degrees of freedom.

Economic Factors include income and income adequacy, with income adequacy being significant in both models. In the *Motivation* model, households reporting “not enough” income had Exp(B) = 0.520 ($p < 0.05$), while those reporting “enough” income recorded Exp(B) = 0.490 ($p < 0.01$). In the *Obstacles* model, inadequate income substantially increased the likelihood of migration (Exp(B) = 1.734, $p < 0.01$). These findings highlight that both objective and perceived income conditions influence migration, consistent with Gray et al. (2012) and Restelli (2023) [16,87]. Monthly income was also significant (Exp(B) = 1.000, $p < 0.01$), indicating that higher income slightly reduces the probability of facing migration obstacles. Such results align with classical migration theory (Todaro, 1969), which links income disparities and job opportunities to mobility decisions [79]. In Egypt, low-income households often rely on migration to diversify income sources, while skilled workers are pulled abroad by higher wages in the EU and Gulf countries [88].

Agricultural Engagement: Employment in agriculture and its share of household income had a negative influence on migration intention. In the Obstacles model, the agricultural income shares yielded Exp(B) = 0.357 ($p < 0.01$), implying that stronger agricultural dependence reduces migration propensity. Similarly, employment in private agricultural work (Exp(B) = 0.328, $p < 0.05$) limited migration motivation. These results are consistent with Gray et al. (2012), who observed that individuals closely tied to farming are less likely to migrate due to attachment to land and local livelihoods [16]. However, environmental pressures—such as declining soil fertility and water scarcity—can offset this stability, especially among younger farmers [16,88].

Table 6. Significant Parameter Estimates for Motivation and Barriers Models

Parameter Estimates	Motivation				Barriers			
	(1) Low		(3) High		(1) Low		(3) High	
	Std. Er.	Exp(B)	Std. Er.	Exp(B)	Std. Er.	Exp(B)	Std. Er.	Exp(B)
Intercept	1.027		0.694		0.963			
Age	0.009	1.046***			0.009	1.023**		
[Income adequacy=1] Not enough	0.306	0.520**	0.216	1.734***				
[Income adequacy=2] Enough	0.257	0.490***						
Agriculture's share of income			0.276	0.590*				
Monthly income			0.000	1.000*			0.000	1.0***

[Gender=1] Females	0.266	1.589*				
[Educational Status=1] Illiterate	0.304	0.497**				
[Educational Status=4] University and post-university	0.248	0.424***				
[Educational Status=5] Institute (above intermediate)	0.368	0.349***				
[Job=3] government employee and work in agriculture	0.373	0.513*				
[Job= 7] Private sector and work in agriculture	0.454	0.328**				
Agricultural share of total income			0.387	0.357***		
[Changes in land productivity =2] As it was 10 years ago			0.303	2.228***		
[Living conditions of the farmer now compared to 10 years ago= 1] Now worse			0.268	0.643*	0.196	0.660**
[Living conditions of the farmer now compared to 10 years ago= 2] As it was 10 years ago					0.252	0.598**
[Change in village conditions now compared to 10 years ago =2.00] As it was 10 years ago			0.321	0.541*	0.216	0.635**
[Agri-land ownership =0] No					0.693	3.815*
[Changes in land productivity =1] Now less than 10 years ago					0.210	1.678**
[Changes in land productivity =2] As it was 10 years ago					0.225	1.456*

a. The reference category is: 2.

b. This parameter is set to zero because it is redundant.

*** Values are significant at P = 0.01, ** Values are significant at P = 0.05, * Values are significant at P = 0.1

Changes in Land Productivity: The Obstacles model showed that changes in land productivity significantly influenced migration barriers. Respondents reporting lower productivity compared with ten years ago were more likely to face migration pressures (Exp(B) = 1.678, $p < 0.05$). At the same time, those whose productivity remained stable also exhibited a moderate increase in migration likelihood (Exp(B) = 1.456, $p < 0.10$). These findings suggest that any stagnation or decline in agricultural productivity acts as a push factor, heightening migration intentions. Conversely, productivity improve-

ments strengthen local economic resilience and reduce migration risks. Similar evidence has been documented in Egypt and Bangladesh, where soil salinity, desertification, and land degradation have undermined rural livelihoods and triggered out-migration [16]. Perceived improvements in living standards and village conditions significantly lowered migration obstacles. For example, respondents reporting worse living conditions recorded Exp(B) = 0.660 ($p < 0.05$), while those perceiving conditions as unchanged showed Exp(B) = 0.598 ($p < 0.05$). These results highlight the importance of subjective well-being and satisfaction in migration decisions. Similar findings in China's Rural Revitalization program show that improving infrastructure and rural services reduces out-migration [81]. Overall, demographic (age, gender, education), economic (income, income adequacy), agricultural (engagement and productivity), and perceptual factors jointly determine migration intentions in rural Egypt. The results confirm that migration decisions stem from intertwined socio-economic and environmental dynamics rather than single determinants [16,29,88].

Overall, the analysis of migration intentions in Fayoum can be clearly understood within the Protection Motivation Theory (PMT) framework. Agricultural land degradation significantly increases threat appraisal by raising farmers' perceived severity and vulnerability, as declining productivity and livelihood insecurity heighten the risk of staying. At the same time, socio-economic resources such as education, remittances, and land assets serve as coping appraisal factors, shaping households' capacity to adapt locally or migrate successfully. The identified drivers of migration—such as environmental stress and unemployment—correspond to dimensions of threat appraisal, while the barriers—including limited resources, family obligations, and lack of skills—reflect coping constraints. Taken together, these findings demonstrate that migration decisions emerge from the interaction between threat and coping appraisals. This provides an integrated explanation of why some households choose to migrate while others remain, underscoring the value of PMT as a guiding theoretical framework for analyzing environmentally induced migration in developing-country contexts.

4. Conclusions and Policy Implications

The complex interrelationship between population dynamics, agricultural systems, and environmental change creates significant challenges for understanding the drivers of migration. Within the Protection Motivation Theory (PMT) framework, our findings show that threat appraisal is heightened by factors such as declining land productivity, inadequate rural services, and perceptions of economic

Formatted: Font: 12 pt

Formatted: Font: 12 pt

Formatted: Font: 12 pt

Formatted: Font: 12 pt

Formatted: Font: 12 pt, Not Italic

Formatted: Font: 12 pt

Formatted: Font: 12 pt

Formatted: Font: 12 pt

Formatted: Font: 12 pt

Formatted: Font: 12 pt, Not Bold

Formatted: Font: (Default) Times New Roman, Not Highlight

Formatted: Font: (Default) Times New Roman, Not Bold

Formatted: Indent: Left: 0"

insecurity, all of which increase the intention to migrate. At the same time, coping appraisal is shaped by socio-economic resources, including education, remittances, and household assets, which determine whether individuals pursue migration or adapt locally. Demographic and social factors, particularly age, gender, and education, play a significant role, with younger and better-educated individuals, especially women, more likely to migrate in search of autonomy and opportunity.

While absolute income was a significant predictor, the perception of income adequacy demonstrated a more nuanced association with migration intentions, indicating the mediating role of subjective well-being. These results affirm the explanatory sufficiency and robustness of PMT in this context. Furthermore, they underscore the necessity of integrating socio-economic and institutional dimensions into the PMT framework for a comprehensive model. Consequently, effective intervention strategies should aim to reduce threat appraisal by implementing sustainable land and water management, poverty alleviation programs, and enhanced rural services. Concurrently, coping appraisal can be strengthened through expanded access to education and vocational training, as well as initiatives that support women's entrepreneurship. Such integrated approaches would not only mitigate the drivers of migration but also foster inclusive and sustainable rural development.

The policy implications based on the empirical findings from both models emphasize the necessity of designing integrated rural development policies that directly address the root causes of migration in Egypt’s rural regions. Since migration intention is powerfully shaped by demographic, economic, and agricultural factors, interventions should prioritize youth employment creation, women’s empowerment, and agricultural modernization. Policies that enhance rural education, vocational training, and entrepreneurship, particularly for young and educated individuals, can convert migration pressure into local productivity gains. Strengthening income adequacy through diversified livelihoods, access to credit, and value-added agribusiness initiatives can mitigate economic push factors. Moreover, the significant influence of land productivity and living conditions suggests the need for investment in climate-smart agriculture, irrigation efficiency, and soil salinity management to stabilize agricultural

income and reduce migration caused by environmental degradation. Gender-sensitive measures are equally critical: supporting women's landownership, improving their access to markets, and promoting their participation in decision-making can lower the gendered drivers of migration. Overall, adopting a comprehensive rural revitalization framework, like China's model, would help retain rural populations by improving infrastructure, public services, and local governance, thereby ensuring that migration becomes a choice driven by opportunity rather than necessity.

The complex interrelationship between population, agriculture, and environmental changes puts a challenge to exploring the drivers of migration and understanding the motivation and barriers of the phenomenon. The findings of this study indicate that demographic and social factors, particularly age, family size, gender, and education, play a crucial role in determining the likelihood of willingness to relocate (internal or international migration). The results suggest that older individuals are more inclined to remain in the village, possibly due to stronger ties to the community or a reduced propensity for change. In contrast, higher educational attainment and greater income levels increase the likelihood of wanting to leave, possibly reflecting aspirations for better opportunities elsewhere. The current results align with the broader literature on migration and residence decisions, particularly in rural contexts. The significant role of these demographic, economic and farming system factors, especially the interaction between these factors is essential for understanding and determining the willingness to migrate. For instance, the interventions for younger individuals and those with higher education levels should be through seeking better opportunities for them similar to those in the urban areas inside the same country.

Formatted: Body Text, Indent: First line: 0", Line spacing: single

The results concluded that young rural women are often willing to migrate to escape restrictive social norms and pursue independent livelihoods, which highlights the role of gender equality and women empowerment in rural areas as a key solution to reduce rural urban migration. While women perceive urban areas as avenues for greater autonomy and employment, incentivising rural women to start their small businesses could be the right strategy to decrease the migration streams, which could be implemented through partnerships with the local NGOs in the rural areas. Interestingly, while income level was a significant factor, perceptions of income adequacy showed a non-linear effect, indicating that satisfaction with income may not directly translate to a desire to stay or leave the village. This complex relationship could be influenced by other unmeasured factors such as quality of life or personal aspirations which requires more investigations in the future.

Migration decisions are not solely based on absolute income levels but also relative perceptions of well-being and future opportunities. Furthermore, the complex relationship between

income, income adequacy, and migration intentions underscores the importance of subjective economic well-being, a factor often emphasized in the livelihoods approach to migration studies. In conclusion, the multinomial logistic regression analysis reveals a nuanced picture of the factors influencing rural residence decisions. The model highlights the importance of demographic variables, while also suggesting that economic considerations, though significant, are influenced by subjective perceptions of income adequacy and satisfaction. These findings could inform policy interventions aimed at rural retention by addressing the specific needs and aspirations of different demographic groups.

However, agricultural land ownership and marital status have been statistically insignificant, these factors are still influential in understanding and explaining the migration intention and decision. Where over 35.8% of respondents reported a decline in land productivity in the past decade with 22.4% actively seeking relocation. Among those willing to migrate, the capital Cairo (40.2%) and in ternational destinations (29.4%) were the most desired. Primary motivations were access to suitable jobs (70.1%) and higher wages (66.8%), whereas major obstacles were relocation diffi culty (54.1%) and high costs (52.7%). Similarly, inadequate services in rural areas also influence migration decisions, with 48.5% of respondents dissatisfied with both educational and healthcare services.

We conclude that rural-urban migration dynamics require integrating strategies to secure good living conditions for rural people and achieve inclusive and sustainable rural development. Overall, these findings contribute theoretically and empirically to the understanding of rural-urban migration dynamics, offering insights into how demographic and economic factors interplay in residence decisions. They also suggest avenues for policy interventions, such as improving local opportunities for women and enhancing education and income adequacy, to potentially reduce migration pressures. At the same time, implementing national projects to sustain soil health (including irrigation and drainage programs) could be a long and sustainable solution for the rural-urban migration in developing countries. Based on these results, we suggest expanding the training centers in the rural areas to equip the rural youth in different agrofood industries, which will secure more jobs and contribute to solving the local communities' issues subsequently reducing the migration streams. Our study underscores the importance of enhancing soil fertility and providing targeted support to vulnerable rural citizens. Policymakers should consider implementing measures such as increasing the availability of affordable sustainable soil health management solutions, offering subsidies, and providing financial counselling and community support programs. Such initiatives can help rural stakeholders overcome the negative impacts of environmental change, fostering more equitable and sustainable rural development.

Author Contributions: Conceptualization, M. Hussien, Ali, Tan and M. Abdelhameed; **methodology**, Tan, M. Abdelhameed, and Ali; **software**, Ali, M. Hussien and M. Abdelhameed; **validation**,

Tan, Ali and M. Hussien; **formal analysis**, M. Abdelhameed and Ali; **investigation**, M. Abdelhameed, M. Hussien, and Ali; **resources**, Ali and Tan; **data curation**, M. Hussien, M. Abdelhameed and Ali; **writing—original draft preparation**, M. Abdelhameed and Ali; **writing**—**review and editing**, M. Hussien, M. Abdelhameed, Ali and Tan; **visualization**, M. Hussien, Tan and M. Abdelhameed; **funding acquisition**, Tan. All authors have read and agreed to the published version of the manuscript.

Funding: This work was supported by The National Natural Science Foundation of China Project: The Economic Transformation and the Development of Regional Agricultural Products Value Chain of ASEAN and China (**71961147002**).

Data Availability Statement: Data will be available based on request.

Acknowledgements: We thank the anonymous reviewers for their valuable help in improving the manuscript.

Conflicts of Interest: Each named author has substantially contributed to conducting the underlying research and drafting this manuscript. Additionally, to the best of our knowledge, the named authors have no conflict of interest, financial or otherwise.

5. References

Appendix A

Table A1 Descriptive statistics of the sample

Sample Characteristics (n= 1782)		F	% N
**Gender**	Female	303	17.00%
	Male	1479	83.00%
**Rural Unit**	Qalhanah	98	5.50%
	Qalamshah	128	7.20%

	AbuJandir	189	10.60%
	AlHajar	162	9.10%
	Tutun	132	7.40%
	Minyat_AlHayt	251	14.10%
	Gerdo	247	13.90%
	AlGharq	121	6.80%
	Mutul	146	8.20%
	AbuSir Difinnu	107	6.00%
	Shidmuh	117	6.60%
	Qasr_AlBasil	84	4.70%
**Marital Status**	under 18 years old	39	2.20%
	Single (never married)	426	23.90%
	Married	1228	68.90%
	Divorced	30	1.70%
	Widower	59	3.30%
**Educational Status**	Illiterate	377	21.70%
	Reads and writes	299	17.20%
	Basic	98	5.60%
	Secondary	497	28.60%
	Institute	106	6.10%
	University and post-university	363	20.90%
**Do you work in agriculture or elsewhere?**	I work in agriculture only	472	26.50%
	Government worker, and I work in agriculture	50	2.80%
	Government worker, and I do not work in agriculture	59	3.30%
	Government employee (specialist - teacher - pharmacist - doctor – etc.), and does not work in agriculture	67	3.70%
	Government employee, and I work in agriculture	91	5.10%
	Private sector (trader, shop owner, driver, etc.), and I do not work in agriculture	283	15.90%
	Private sector, working in agriculture	326	18.30%
	I do not work	434	24.40%
**Do you own agricultural land?**	No	738	41.40%
	Yes	1044	58.60%

Table A2 Farming system Characteristics and migration intention of the selected respondents N= (1782)

**Items**	Responses	N	%
**Has the area of land you own now changed from 10 years ago?**	The area is now less than 10 years ago	248	22.9%
	The area has not changed	502	46.3%
	The area is now larger than 10 years ago.	335	30.9%

~~**Has the area of land you cultivate changed now compared to 10 years ago?**~~	~~The area is now less than 10 years ago~~<br>~~The area has not changed~~<br>~~The area is now larger than 10 years ago.~~	~~309~~<br>~~744~~<br>~~343~~	~~22.1%~~<br>~~53.3%~~<br>~~24.6%~~
~~**How about the rice cultivation in your crop pattern?**~~	~~Within the crop structure of only 10 years~~<br>~~Within the current crop structure and for 10 years~~<br>~~Within the current crop composition only~~	~~171~~<br>~~66~~<br>~~46~~	~~60.4%~~<br>~~23.3%~~<br>~~16.3%~~
~~**How about the sugar beet cultivation in your crop pattern?**~~	~~Within the crop structure of only 10 years~~<br>~~Within the current crop structure and for 10 years~~<br>~~Within the current crop composition only~~	~~158~~<br>~~165~~<br>~~124~~	~~35.3%~~<br>~~36.9%~~<br>~~27.7%~~
~~**Has there been a change in the productivity of the land you cultivate?**~~	~~Productivity is now less than 10 years ago~~<br>~~Productivity has not changed~~<br>~~Productivity is now larger than 10 years ago.~~	~~503~~<br>~~516~~<br>~~385~~	~~35.8%~~<br>~~36.8%~~<br>~~27.4%~~
~~**Total income (monthly)**~~	~~less than 2000 LE~~<br>~~2000 - 4000~~<br>~~4000- 6000~~<br>~~more than 6000~~	~~419~~<br>~~774~~<br>~~378~~<br>~~211~~	~~23.5%~~<br>~~43.4%~~<br>~~21.2%~~<br>~~11.8%~~
~~**Income adequacy**~~	~~Not enough~~<br>~~Somewhat enough~~<br>~~Enough~~	~~624~~<br>~~684~~<br>~~474~~	~~35.0%~~<br>~~38.4%~~<br>~~26.6%~~
~~**What do you think of the current state of agricultural production?**~~	~~Now worse~~<br>~~As it was 10 years ago~~<br>~~Now better~~	~~788~~<br>~~321~~<br>~~673~~	~~44.2%~~<br>~~18.0%~~<br>~~37.8%~~
~~**What do you think about the living conditions of the farmer now compared to 10 years ago?**~~	~~Now worse~~<br>~~As it was 10 years ago~~<br>~~Now better~~	~~952~~<br>~~257~~<br>~~573~~	~~53.4%~~<br>~~14.4%~~<br>~~32.2%~~
~~**What do you think of the village now compared to 10 years ago?**~~	~~Now worse~~<br>~~As it was 10 years ago~~<br>~~Now better~~	~~588~~<br>~~295~~<br>~~899~~	~~33.0%~~<br>~~16.6%~~<br>~~50.4%~~
~~**Have you ever changed your place of residence (for 6 months or more)?**~~	~~No~~<br>~~Yes~~	~~1485~~<br>~~297~~	~~83.3%~~<br>~~16.7%~~

**Have you worked outside Fayoum Governorate (for 6 months or more)?**	No<br>Yes	1079<br>703	60.5%<br>39.5%
**Do you want to change your place of residence and leave the village?**	No<br>Maybe<br>Yes	805<br>577<br>400	45.2%<br>32.4%<br>22.4%
**I plan to change residence to ….**	Greater Cairo<br>Alexandria<br>Cities of the Suez Canal, Sinai and the Red Sea<br>Delta Governorates<br>Upper Egypt Governorates<br>Fayoum City<br>Outside Egypt	314<br>47<br>31<br>27<br>12<br>120<br>230	40.2%<br>6.0%<br>4.0%<br>3.5%<br>1.5%<br>15.4%<br>29.4%
**Do you want to change your job location or look for work outside your village?**	No<br>Maybe<br>Yes	681<br>512<br>589	38.2%<br>28.7%<br>33.1%
**Change of job location or look for work to**	Greater Cairo<br>Alexandria<br>Cities of the Suez Canal, Sinai and the Red Sea<br>Delta Governorates<br>Upper Egypt Governorates<br>Fayoum City<br>Outside Egypt	303<br>48<br>28<br>29<br>9<br>94<br>296	37.5%<br>5.9%<br>3.5%<br>3.6%<br>1.1%<br>11.6%<br>36.7%
**If legal travel is not available, is it possible to travel illegally?**	No<br>Maybe<br>Yes	1138<br>275<br>369	63.9%<br>15.4%<br>20.7%
**If you leave the village and work in another governorate, you will take your family with you.**	I won't take them at all<br>I might take them, and I might not<br>I'll take them after I settle in<br>I'll take some of them to help me there<br>I'll take them all, of course	439<br>323<br>123<br>386<br>511	24.6%<br>18.1%<br>6.9%<br>21.7%<br>28.7%
**If you work in another governorate, will you change your place of residence on your official ID?**	I won't change it at all<br>I won't change it unless I have to<br>I might change it, and I might not<br>I will change it after it settles<br>I will change it of course	431<br>357<br>377<br>337<br>280	24.2%<br>20.0%<br>21.2%<br>18.9%<br>15.7%
**If you moved to another governorate and liked the way you**	No<br>Maybe	360<br>800	20.2%

~~lived there, would you advise others to move there?~~		

1. Hermans, K.; McLeman, R. Climate Change, Drought, Land Degradation and Migration: Exploring the Linkages. *Curr Opin Environ Sustain* 2021, *50*, 236–244.
2. Gu, W.; Ma, G.; Wang, R.; Scherer, L.; He, P.; Xia, L.; Zhu, Y.; Bi, J.; Liu, B. Climate Adaptation through Crop Migration Requires a Nexus Perspective for Environmental Sustainability in the North China Plain. *Nat Food* **2024**, *5*, 569–580, doi:10.1038/s43016-024-01008-8.
3. Salerno, J.; Gaughan, A.E.; Warrier, R.; Boone, R.; Stevens, F.R.; Keys, P.W.; Mangewa, L.J.; Mombo, F.M.; de Sherbinin, A.; Hartter, J.; et al. Rural Migration under Climate and Land Systems Change. *Nat Sustain* **2024**, doi:10.1038/s41893-024-01396-6.
4. McLeman, R. *Migration and Land Degradation: Recent Experience and Future Trends*; Global Land Outlook ; Global Land Outlook working paper. UNCCD, 2017;
5. Liu, Y.; Zhang, R.; Li, M.; Zhou, C. What Factors Influence Rural-to-Urban Migrant Peasants to Rent out Their Household Farmland? Evidence from China's Pearl River Delta. *Land (Basel)* **2020**, *9*, 1–20, doi:10.3390/land9110418.
6. Davis, J.; Lopez-Carr, D. Migration, Remittances and Smallholder Decision-Making: Implications for Land Use and Livelihood Change in Central America. *Land use policy* **2014**, *36*, 319–329, doi:10.1016/j.landusepol.2013.09.001.
7. Nguyen, C.V.; Nguyen, H.Q. Do Internal and International Remittances Matter to Health, Education and Labor of Children and Adolescents? The Case of Vietnam. *Child Youth Serv Rev* **2015**, *58*, 28–34, doi:10.1016/j.childyouth.2015.09.002.
8. Hermans, K.; Müller, D.; O'Byrne, D.; Olsson, L.; Stringer, L.C. Land Degradation and Migration. *Nat Sustain* **2023**, *6*, 1503–1505, doi:10.1038/s41893-023-01231-4.
9. Gray, C.; Bilsborrow, R. Environmental Influences on Human Migration in Rural Ecuador. *Demography* **2013**, *50*, 1217–1241, doi:10.1007/s13524-012-0192-y.
10. Appadurai, A. The Capacity to Aspire: Culture and the Terms of Recognition. *Cultural politics in a global age: Uncertainty, solidarity and innovation* **2007**, 29.
11. Johnson, R.L. Reversing Channels and Unsettling Binaries: Rethinking Migration and Agrarian Change under Expanded Border and Immigration Enforcement. *Land (Basel)* **2021**, *10*, 1–19, doi:10.3390/land10030228.
12. De Brauw, A. Migration Out of Rural Areas and Implications for Rural Livelihoods. **2019**, doi:10.1146/annurev-resource-100518.
13. Soto Nishimura, A.; Czaika, M. Exploring Migration Determinants: A Meta-Analysis of Migration Drivers and Estimates. *J Int Migr Integr* **2024**, *25*, 621–643, doi:10.1007/s12134-023-01091-z.
14. International Organization for Migration (IOM) *World Migration Report 2020*; IOM World Migration Report; United Nations, 2019; ISBN 9789290687894.
15. Kafando, W.A.; Sakurai, T. Armed Conflicts and Household Food Insecurity: Effects and Mechanisms. *Agricultural Economics* **2024**, doi:10.1111/agec.12814.
16. Gray, C.L.; Mueller, V. Natural Disasters and Population Mobility in Bangladesh. *Proceedings of the National Academy of Sciences* **2012**, *109*, 6000–6005, doi:10.1073/pnas.1115944109.

17. Sedova, B.; Kalkuhl, M. Who Are the Climate Migrants and Where Do They Go? Evidence from Rural India. *World Dev* **2020**, *129*, 104848, doi:https://doi.org/10.1016/j.worlddev.2019.104848.
18. Adger, W.N.; Kelly, P.M.; Winkels, A.; Huy, L.Q.; Locke, C. Migration, Remittances, Livelihood Trajectories, and Social Resilience. *AMBIO: A Journal of the Human Environment* **2002**, *31*, 358–366, doi:10.1579/0044-7447-31.4.358.
19. Henry, S.; Schoumaker, B.; Beauchemin, C. The Impact of Rainfall on the First Out-Migration: A Multilevel Event-History Analysis in Burkina Faso. *Population and Environment - POP ENVIRON* **2004**, *25*, 423–460, doi:10.1023/B:POEN.0000036928.17696.e8.
20. Liu, Y.; Li, J.; Yang, Y. Strategic Adjustment of Land Use Policy under the Economic Transformation. *Land use policy* **2018**, *74*, 5–14, doi:https://doi.org/10.1016/j.landusepol.2017.07.005.
21. Xu, D.; Yong, Z.; Deng, X.; Zhuang, L.; Qing, C. Rural-Urban Migration and Its Effect on Land Transfer in Rural China. *Land (Basel)* **2020**, *9*, doi:10.3390/land9030081.
22. Al-Maruf, A.; Kanak Pervez, A.K.M.; Sarker, P.K.; Rahman, M.S.; Ruiz-Menjivar, J. Exploring the Factors of Farmers' Rural–Urban Migration Decisions in Bangladesh. *Agriculture (Switzerland)* **2022**, *12*, doi:10.3390/agriculture12050722.
23. Awad, I. The Political Economy of Egyptian Migration to Europe in the 2020s. In *IMISCOE Research Series*; Springer Science and Business Media B.V., 2024; Vol. Part F1581, pp. 347–364.
24. Moore, M.; Wesselbaum, D. Climatic Factors as Drivers of Migration: A Review. *Environ Dev Sustain* **2023**, *25*, 2955–2975, doi:10.1007/s10668-022-02191-z.
25. Soto Nishimura, A.; Czaika, M. Exploring Migration Determinants: A Meta-Analysis of Migration Drivers and Estimates. *J Int Migr Integr* **2024**, *25*, 621–643, doi:10.1007/s12134-023-01091-z.
26. Ajaero, C.K.; Onokala, P.C. The Effects of Rural-Urban Migration on Rural Communities of Southeastern Nigeria. *Int J Popul Res* **2013**, *2013*, 1–10, doi:10.1155/2013/610193.
27. Majeed, A.; Muhammad, Z. Salinity: A Major Agricultural Problem—Causes, Impacts on Crop Productivity and Management Strategies. In *Plant Abiotic Stress Tolerance: Agronomic, Molecular and Biotechnological Approaches*; Hasanuzzaman, M., Hakeem, K.R., Nahar, K., Alharby, H.F., Eds.; Springer International Publishing: Cham, 2019; pp. 83–99 ISBN 978-3-030-06118-0.
28. Schwilch, G.; Liniger, H.P.; Hurni, H. Sustainable Land Management (SLM) Practices in Drylands: How Do They Address Desertification Threats? *Environ Manage* **2014**, *54*, 983–1004, doi:10.1007/s00267-013-0071-3.
29. Abdelwahed, A.; Goujon, A.; Jiang, L. The Migration Intentions of Young Egyptians. *Sustainability (Switzerland)* **2020**, *12*, 1–38, doi:10.3390/su12239803.
30. Zohry, A. *Interrelationships between Internal and International Migration in Egypt: A Pilot Study*; 2005;
31. Almulhim, A.I.; Alverio, G.N.; Sharifi, A.; Shaw, R.; Huq, S.; Mahmud, M.J.; Ahmad, S.; Abubakar, I.R. Climate-Induced Migration in the Global South: An in Depth Analysis. *npj Climate Action* **2024**, *3*, doi:10.1038/s44168-024-00133-1.
32. Nagabhatla, N.; Pouramin, P.; Brahmbhatt, R.; Fioret, C.; Glickman, T.; Newbold, K.B.; Smakhtin, V. Water and Migration: A Global Overview. *UNU-INWEH report series* **2020**, *10*.
33. Zaveri, E.; Russ, J.; Khan, A.; Damania, R.; Borgomeo, E.; Jägerskog, A. *Ebb and Flow, Volume 1*; The World Bank, 2021; ISBN 978-1-4648-1745-8.

34. Mahmoud, M.A. Impact of Climate Change on the Agricultural Sector in Egypt. In *Conventional Water Resources and Agriculture in Egypt*; Negm, A.M., Ed.; Springer International Publishing: Cham, 2019; pp. 213–227 ISBN 978-3-319-95065-5.
35. Viala, E. Water for Food, Water for Life a Comprehensive Assessment of Water Management in Agriculture. *Irrigation and Drainage Systems* **2008**, *22*, 127–129, doi:10.1007/s10795-008-9044-8.
36. Zaveri, E.; Russ, J.; Khan, A.; Damania, R.; Borgomeo, E.; Jägerskog, A. *Ebb and Flow: Volume 1. Water, Migration, and Development*; World Bank Publications: Washington, DC, 2021; ISBN 1464817472.
37. Jha, C.K.; Gupta, V.; Chattopadhyay, U.; Amarayil Sreeraman, B. Migration as Adaptation Strategy to Cope with Climate Change: A Study of Farmers' Migration in Rural India. *Int J Clim Chang Strateg Manag* **2018**, *10*, 121–141.
38. Pingali, P.L. Green Revolution: Impacts, Limits, and the Path Ahead. *Proceedings of the national academy of sciences* **2012**, *109*, 12302–12308.
39. Elmoghazy, A.M.; Elshenawy, M.M. Sustainable Cultivation of Rice in Egypt. In *Handbook of Environmental Chemistry*; Springer Verlag, 2019; Vol. 76, pp. 119–144.
40. Morton, J.F. The Impact of Climate Change on Smallholder and Subsistence Agriculture. *Proceedings of the national academy of sciences* **2007**, *104*, 19680–19685.
41. Eshetu, F.; Beshir, M. Dynamics and Determinants of Rural-Urban Migration in Southern Ethiopia. *J Dev Agric Econ* **2017**, *9*, 328–340.
42. Selod, H.; Shilpi, F. Rural-Urban Migration in Developing Countries: Lessons from the Literature. *Reg Sci Urban Econ* **2021**, *91*, doi:10.1016/j.regsciurbeco.2021.103713.
43. Asfaw, W.; Tolossa, D.; Zeleke, G. Causes and Impacts of Seasonal Migration on Rural Livelihoods: Case Studies from Amhara Region in Ethiopia. *Norsk Geografisk Tidsskrift–Norwegian Journal of Geography* **2010**, *64*, 58–70.
44. Nweke, A.C. Rural-Urban Migration in Nigeria, Implication on the Development of the Society: Anambra State as the Focus of the Study. *Journal of Public Administration and Governance* **2019**, *9*, 209–216.
45. United Nations *International Migration 2020 Highlights*; 2021;
46. Geddes, A.; Adger, W.N.; Arnell, N.W.; Black, R.; Thomas, D.S.G. Migration, Environmental Change, and the 'Challenges of Governance.' *Environ Plann C Gov Policy* **2012**, *30*, 951–967.
47. Renaud, F.G.; Dun, O.; Warner, K.; Bogardi, J. A Decision Framework for Environmentally Induced Migration. *International Migration* **2011**, *49*, e5–e29.
48. Qin, H.; Liao, T.F. Labor Out-Migration and Agricultural Change in Rural China: A Systematic Review and Meta-Analysis. *J Rural Stud* **2016**, *47*, 533–541, doi:10.1016/j.jrurstud.2016.06.020.
49. Qin, H.; Liao, T.F. Labor Out-Migration and Agricultural Change in Rural China: A Systematic Review and Meta-Analysis. *J Rural Stud* **2016**, *47*, 533–541, doi:10.1016/j.jrurstud.2016.06.020.
50. Bodvarsson, Ö.B.; Van den Berg, H. The Determinants of International Migration: Theory. In *The Economics of Immigration*; Springer New York: New York, NY, 2013; pp. 27–57.
51. Fafchamps, M.; Shilpi, F. Determinants of the Choice of Migration Destination. *Oxf Bull Econ Stat* **2013**, *75*, 388–409, doi:https://doi.org/10.1111/j.1468-0084.2012.00706.x.
52. Selod, H.; Shilpi, F. Rural-Urban Migration in Developing Countries: Lessons from the Literature. *Reg Sci Urban Econ* **2021**, *91*, doi:10.1016/j.regsciurbeco.2021.103713.

53. Bazzi, S.; Gaduh, A.; Rothenberg, A.D.; Wong, M. Unity in Diversity? How Intergroup Contact Can Foster Nation Building. *American Economic Review* **2019**, *109*, 3978–4025.
54. Taylor, J.E.; Martin, P.L. Human Capital: Migration and Rural Population Change. *Handbook of agricultural economics* **2001**, *1*, 457–511.
55. Luo, C.; Yan, X.; Wang, B.; Ali, A. Long-Term Adaptation Mitigates the Promotion Effect of Air Pollution on Short-Term Population Movements. *Front Environ Sci* **2025**, *13*, 1620499, doi:10.3389/FENVS.2025.1620499/BIBTEX.
56. Lu, H.; Kandilov, I.T.; Zhu, R. The Impact of Internal Migration on the Health of Rural Migrants: Evidence from Longitudinal Data in China. *J Dev Stud* **2020**, *56*, 840–855.
57. Xiang, A.O.; Jiang, D.; Zhong, Z. The Impact of Rural–Urban Migration on the Health of the Left-behind Parents. *China Economic Review* **2016**, *37*, 126–139.
58. De Jong, G.F. Expectations, Gender, and Norms in Migration Decision-Making. *Popul Stud (NY)* **2000**, *54*, 307–319.
59. Lee, E.S. A Theory of Migration. *Demography* **1966**, *3*, 47–57, doi:10.2307/2060063/METRICS.
60. Ravenstein, E.G. The Laws of Migration. *Journal of the Statistical Society of London* **1885**, *48*, 167–227, doi:10.2307/2979181.
61. De Haas, H. Migration and Development: A Theoretical Perspective. *International Migration Review* **2010**, *44*, 227–264, doi:10.1111/j.1747-7379.2009.00804.x.
62. de Haas, H. A Theory of Migration: The Aspirations-Capabilities Framework. *Comp Migr Stud* **2021**, *9*, doi:10.1186/s40878-020-00210-4.
63. Rogers, R.W. A Protection Motivation Theory of Fear Appeals and Attitude Change1. *J Psychol* **1975**, *91*, 93–114, doi:10.1080/00223980.1975.9915803.
64. Maddux, J.E.; Rogers, R.W. Protection Motivation and Self-Efficacy: A Revised Theory of Fear Appeals and Attitude Change. *J Exp Soc Psychol* **1983**, *19*, 469–479, doi:https://doi.org/10.1016/0022-1031(83)90023-9.
65. Krejcie, R. V.; Morgan, D.W. Determining Sample Size for Research Activities. *Educ Psychol Meas* **1970**, *30*, 607–610, doi:10.1177/001316447003000308.
66. Cochran, W.G. *Sampling Techniques*; john wiley & sons, 1977; ISBN 8126515244.
67. Wooldridge, J.M. *Econometric Analysis of Cross Section and Panel Data*; MIT press, 2010; ISBN 0262296799.
68. Hua, C.; Choi, Y.J.; Shi, Q. Companion to BER 642: Advanced Regression Methods. *Bookdown. Retrieved August* **2021**, *5*, 2021.
69. Deininger, K.; Jin, S. Tenure Security and Land-Related Investment: Evidence from Ethiopia. *Eur Econ Rev* **2006**, *50*, 1245–1277, doi:https://doi.org/10.1016/j.euroecorev.2005.02.001.
70. Radwan, S.; Lee, E. *Agrarian Change in Egypt: An Anatomy of Rural Poverty*; Routledge, 2022; ISBN 1003313418.
71. Mulder, C.H.; Cooke, T.J. Family Ties and Residential Locations. *Popul Space Place* **2009**, *15*, 299–304.
72. White, M.J.; Lindstrom, D.P. Internal Migration. In *Handbook of population*; Springer, 2005; pp. 311–346.
73. Chant, S. Households, Gender and Rural-Urban Migration: Reflections on Linkages and Considerations for Policy. *Environ Urban* **1998**, *10*, 5–22.
74. Lagakos, D. Urban-Rural Gaps in the Developing World: Does Internal Migration Offer Opportunities? *Journal of Economic Perspectives* **2020**, *34*, 174–192, doi:10.1257/jep.34.3.174.

75. Yu, Z.; Zhang, H.; Tao, Z.; Liang, J. Amenities, Economic Opportunities and Patterns of Migration at the City Level in China. *Asian and Pacific Migration Journal* **2019**, *28*, 3–27, doi:10.1177/0117196819832054.
76. Nguyen, C.V.; Nguyen, H.Q. Do Internal and International Remittances Matter to Health, Education and Labor of Children and Adolescents? The Case of Vietnam. *Child Youth Serv Rev* **2015**, *58*, 28–34, doi:10.1016/j.childyouth.2015.09.002.
77. Skeldon, R. Interlinkages between Internal and International Migration and Development in the Asian Region. *Popul Space Place* **2006**, *12*, 15–30, doi:https://doi.org/10.1002/psp.385.
78. Chen, Y.; Luo, P.; Chang, T. Urbanization and the Urban-Rural Income Gap in China: A Continuous-wavelet Coherency Analysis. *Sustainability (Switzerland)* **2020**, *12*, doi:10.3390/su12198261.
79. Todaro, M.P.; Maruszko, L. Illegal Migration and US Immigration Reform: A Conceptual Framework. *Popul Dev Rev* **1987**, 101–114.
80. Zapata-Barrero, R.; Awad, I. Introduction: Mediterranean Migration Studies – A Research Agenda for the Coming Years. In *IMISCOE Research Series*; Springer Science and Business Media B.V., 2024; Vol. Part F1581, pp. 1–7.
81. Ren, T.; Wang, N.; Xiao, H.; Zhou, Z. Efficiency of Funding to Rural Revitalization and Regional Heterogeneity of Technologies in China: Dynamic Network Nonconvex Metafrontiers. *Socioecon Plann Sci* **2024**, *92*, doi:10.1016/j.seps.2024.101825.
82. Ghio, D.; Goujon, A.; Natale, F.; Alfredo, A.; Petroliagkis, T. Assessing Populations Exposed to Climate Change: A Focus on Africa in a Global Context. *Popul Environ* **2023**, *45*, doi:10.1007/s11111-023-00439-y.
83. King, R.; Skeldon, R. 'Mind the Gap!'Integrating Approaches to Internal and International Migration. *J Ethn Migr Stud* **2010**, *36*, 1619–1646.
84. Radwan, S.; Lee, E. *Agrarian Change in Egypt: An Anatomy of Rural Poverty*; Routledge, 2022; ISBN 1003313418.
85. Aydemir, A.B.; Kırdar, M.G.; Torun, H. The Effect of Education on Internal Migration of Young Men and Women: Incidence, Timing, and Type of Migration. *Labour Econ* **2022**, *74*, 102098, doi:10.1016/J.LABECO.2021.102098.
86. Anastasiadou, A.; Kim, J.; Sanlitürk, E.; de Valk, H.A.G.; Zagheni, E. Gender Differences in the Migration Process: A Narrative Literature Review. *Popul Dev Rev* **2024**, *50*, 961–996, doi:10.1111/PADR.12677.
87. Restelli, G. Development and International Migration: The Effect of Income on Regular and Irregular Migration Intentions to Europe. *Popul Dev Rev* **2023**, *49*, 135–174, doi:10.1111/PADR.12525.
88. Brodmann, S.; Pouget, Y.; Gatti, R. *Labor Mobility in the Middle East and North Africa: Challenges and Opportunities*; World Bank, Washington, DC, 2010;

Appendix

Table A.1: Selected villages in the *Itsa* districts

**Cluster**	**Villages**	**Total population**	**Selected respondents (N)**	**Selected sample (%)**
Cluster 1 (Desert Hinterland)	*Qalhana, Qolamsha, Qasr El-Basil, and Tatoun*	166878	431	24.77%
Cluster 2 (*Bahr Youssef* Delta)	*Gerdo, Matoul, and Abousir*	197868	488	28.05%
Cluster 3 (*Bahr El-Nazla* and *Bahr El-Banat* lands)	Abogendeer and Menyet El-Heet	183884	430	24.71%
Cluster 4 (*El-Gharq El-Soltany* Basin)	*Shidmoh, El-Gharq, El-Hagar*, and *Anak*	170524	391	22.47%
Total				100 %

Table A.2: Descriptive statistics of the sample

**Sample Characteristics (n= 1782)**		**F**	**% N**
**Gender**	Female	303	17.00%
	Male	1479	83.00%
**Rural Unit**	*Qalhanah*	98	5.50%
	Qalamshah	128	7.20%
	AbuJandir	189	10.60%
	AlHajar	162	9.10%
	Tutun	132	7.40%
	Minyat_AlHayt	251	14.10%
	Gerdo	247	13.90%
	AlGharq	121	6.80%
	Mutul	146	8.20%
	AbuSir-Difinnu	107	6.00%
	Shidmuh	117	6.60%
	Qasr_AlBasil	84	4.70%
**Marital Status**	under 18 years old	39	2.20%
	Single (never married)	426	23.90%
	Married	1228	68.90%
	Divorced	30	1.70%
	Widower	59	3.30%
**Educational Status**	Illiterate	377	21.70%
	Reads and writes	299	17.20%
	Basic	98	5.60%
	Secondary	497	28.60%

Formatted: Comment Text
Formatted: Font: 11 pt, Bold
Formatted: Font: 11 pt
Formatted: Font: 11 pt, Italic
Formatted: Font: 11 pt
Formatted: Font: 11 pt, Font color: Auto
Formatted: Font: 11 pt
Formatted Table
Formatted: Justified
Formatted: Font: Bold
Formatted Table
Formatted: Font: Italic
Formatted: Font: Italic
Formatted: Font: Italic
Formatted: Font: Italic
Formatted: Font: Italic
Formatted: Font: Italic
Formatted: Font: Italic
Formatted: Font: Italic
Formatted: Font: Italic
Formatted: Font: Italic
Formatted: Font: Italic
Formatted: Font: Italic

	Institute	106	6.10%
	University and post-university	363	20.90%
**Do you work in agriculture or elsewhere?**	I work in agriculture only	472	26.50%
	Government worker, and I work in agriculture	50	2.80%
	Government worker, and I do not work in agriculture	59	3.30%
	Government employee (specialist - teacher - pharmacist - doctor - etc.), and does not work in agriculture	67	3.70%
	Government employee, and I work in agriculture	91	5.10%
	Private sector (trader, shop owner, driver, etc.), and I do not work in agriculture	283	15.90%
	Private sector, working in agriculture	326	18.30%
	I do not work	434	24.40%
**Do you own agricultural land?**	No	738	41.40%
	Yes	1044	58.60%

Table A.3: Farming system Characteristics and migration intention of the selected respondents N= (1782)

**Items**	**Responses**	**N**	**%**
**Has the area of land you own now changed from 10 years ago?**	The area is now less than 10 years ago	248	22.9%
	The area has not changed	502	46.3%
	The area is now larger than 10 years ago.	335	30.9%
**Has the area of land you cultivate changed now compared to 10 years ago?**	The area is now less than 10 years ago	309	22.1%
	The area has not changed	744	53.3%
	The area is now larger than 10 years ago.	343	24.6%
**How about the rice cultivation in your crop pattern?**	Within the crop structure of only 10 years	171	60.4%
	Within the current crop structure and for 10 years	66	23.3%
	Within the current crop composition only	46	16.3%
**How about the sugar beet cultivation in your crop pattern?**	Within the crop structure of only 10 years	158	35.3%
	Within the current crop structure and for 10 years	165	36.9%
	Within the current crop composition only	124	27.7%
	Productivity is now less than 10 years ago	503	35.8%

Formatted: Left

Formatted Table

Formatted: Font: Bold

**Has there been a change in the productivity of the land you cultivate?**	Productivity has not changed	516	36.8%
	Productivity is now larger than 10 years ago.	385	27.4%
**Total income (monthly)**	less than 2000 LE	419	23.5%
	2000 - 4000	774	43.4%
	4000- 6000	378	21.2%
	more than 6000	211	11.8%
**Income adequacy**	Not enough	624	35.0%
	Somewhat enough	684	38.4%
	Enough	474	26.6%
**What do you think of the current state of agricultural production?**	Now worse	788	44.2%
	As it was 10 years ago	321	18.0%
	Now better	673	37.8%
**What do you think about the living conditions of the farmer now compared to 10 years ago?**	Now worse	952	53.4%
	As it was 10 years ago	257	14.4%
	Now better	573	32.2%
**What do you think of the village now compared to 10 years ago?**	Now worse	588	33.0%
	As it was 10 years ago	295	16.6%
	Now better	899	50.4%
**Have you ever changed your place of residence (for 6 months or more)?**	No	1485	83.3%
	Yes	297	16.7%
**Have you worked outside Fayoum Governorate (for 6 months or more)?**	No	1079	60.5%
	Yes	703	39.5%
**Do you want to change your place of residence and leave the village?**	No	805	45.2%
	Maybe	577	32.4%
	Yes	400	22.4%
**I plan to change residence to ….**	Greater Cairo	314	40.2%
	Alexandria	47	6.0%
	Cities of the Suez Canal, Sinai and the Red Sea	31	4.0%
	Delta Governorates	27	3.5%
	Upper Egypt Governorates	12	1.5%
	Fayoum City	120	15.4%
	Outside Egypt	230	29.4%
**Do you want to change your job location or look for work outside your village?**	No	681	38.2%
	Maybe	512	28.7%
	Yes	589	33.1%
**Change of job location or look for work to**	Greater Cairo	303	37.5%
	Alexandria	48	5.9%

	Cities of the Suez Canal, Sinai and the Red Sea	28	3.5%
	Delta Governorates	29	3.6%
	Upper Egypt Governorates	9	1.1%
	Fayoum City	94	11.6%
	Outside Egypt	296	36.7%
**If legal travel is not available, is it possible to travel illegally?**	No	1138	63.9%
	Maybe	275	15.4%
	Yes	369	20.7%
**If you leave the village and work in another governorate, you will take your family with you.**	I won't take them at all	439	24.6%
	I might take them, and I might not	323	18.1%
	I'll take them after I settle in	123	6.9%
	I'll take some of them to help me there	386	21.7%
	I'll take them all, of course	511	28.7%
**If you work in another governorate, will you change your place of residence on your official ID?**	I won't change it at all	431	24.2%
	I won't change it unless I have to	357	20.0%
	I might change it, and I might not	377	21.2%
	I will change it after it settles	337	18.9%
	I will change it of course	280	15.7%
**If you moved to another governorate and liked the way you lived there, would you advise others to move there?**	No	360	20.2%
	Maybe	800	44.9%
	Yes	622	34.9%

1220 **Formatted:** Comment Text, Adjust space between Latin and Asian text, Adjust space between Asian text and numbers

RESPONSE TO REVIEWER COMMENTS FORM
FOR PLOS ONE

Name of author(s): Muhammad Hussien, Abdelrahman Ali, Yanwen Tan, Mohamed Abdelhameed
Manuscript ID: PONE-D-25-21257
Title: **"**Agricultural Land Degradation Consequences as a Migration Driver in Egypt"

Comments reviewer I	***Author's response***
Conceptualization of the paper begins with push-pull theory of Everett Lee (1966) and basis of which is "laws of migration" Revenstein (1885). Therefore, it would be better if we acknowledge their prioneering contribution of the knowledgebase.	We sincerely thank the reviewer for this valuable observation. We agree that the conceptualization of migration studies is deeply rooted in Everett Lee's Push-Pull Theory (1966) and Ravenstein's "Laws of Migration" (1885). We have therefore revised the introduction to explicitly acknowledge their pioneering contributions and to situate our study within this historical foundation. See the revised version of the manuscript.
Page 10 - "Figure 3. Migration intention under the agriculture land degredation" is not well connected with protection motivation theory (PMT). It needs more explanation in the text.	We thank the reviewer for pointing this out. We agree that Figure 3 ("Migration intention under the agricultural land degradation") required a stronger linkage to the Protection Motivation Theory (PMT). We revised the manuscript by adding explanatory text directly before and after Figure 3 to clarify how PMT constructs (threat appraisal and coping appraisal) are reflected in the figure. This ensures that the theoretical framework and empirical findings are conceptually aligned.
Page 10 – Section 2.1 Study area and sampling strategy: The paper is silent about sample size (1782) how it has been calculated and whether it is representative or not for a particular population. It needs to be well established that the method of calculation and its representativeness.	We thank the reviewer for raising this point. In the revised manuscript, we clarified that the sample size of 1,782 households was determined using Cochran's formula (1977) at a 95% confidence level and 5% margin of error, adjusted for Fayoum's rural population. A cluster sampling approach was applied across the 12 villages of Itsa district, grouped into four clusters. Within each cluster, households were selected through systematic random sampling, with one adult respondent interviewed per household. This ensured statistical representativeness of the rural population.
In the same page, line 254-56 is not clear.	We thank the reviewer for pointing out this unclear sentence. We have revised it to improve clarity and readability. The revised version corrects the grammar and removes the confusing expression, while retaining the intended meaning about Fayoum's significance as a migration-exporting governorate and its poverty level.

Page 11, line 277-78 – it says, “we selected the respondents randomly among the twelve rural villages in Itsa district”. It is not clear that whether it is individual survey or household survey. How was the sample from 12 villages randomly selected? Is this a cluster sampling? Or are these all 12 villages put together in a single sampling unit? They have to be clearly spelled out how the sample is random.	We thank the reviewer for this important observation. We have clarified in the revised manuscript that the survey was household-based and that one adult respondent was randomly selected from each sampled household. The 12 villages in Itsa district were treated as clusters, and within each village, households were selected using systematic random sampling from official village household lists provided by local authorities. This approach ensured both randomness and proportional representation of the selected villages.
Section 3.3. “Model fitting and pseudo R square”: It would be better to bring this section at the begging of the section 3 just before table 1. Then, go for the parameter estimates.	We thank the reviewer for this constructive suggestion. We agree that placing the subsection “Model fitting and pseudo R square” at the beginning of Section 3 improves the logical flow of results. Accordingly, we have moved this subsection to appear immediately before Table 1, followed by the parameter estimates.
It is confusing that table 1 displays both coefficients (b) and odds ratios (exp(b)) and level of significance is indicated in coefficients, however, the analysis has been made using exp(b). So, be clear and consistent between text and table.	We appreciate the reviewer’s careful reading of Table 2. We agree that the presentation was confusing because both coefficients (b) and odds ratios (Exp(b)) were reported, while the analysis and discussion were based on odds ratios. To ensure clarity and consistency, we have revised Table 2 to present only the odds ratios (Exp(b)) along with their corresponding significance levels. The text discussion has also been aligned to reference odds ratios consistently.
Section 3.2.5 Drivers and Barriers of migration: This is main heading, whereas in the subsection B, it is “obstacles”. Be consistent.<br>○ Author has to establish those questions included to inform drivers and barriers are valid and they would well represent the drivers and barriers.<br>○ Analysis of drivers and barriers of migration is weak. Authors need to find out the appropriate advance statistical method for measuring the drivers and barriers. They should be measured against socio-economic variables so that variation in drivers and barriers could be informed. Indexing into a single composite variable for bother drivers and obstacles may one option. And, retreatment of three level of scales – agree,	**We thank the reviewer for this comprehensive feedback. In response, we made three major revisions:**<br>- Terminology Consistency: We standardized the terminology by using “Drivers and Barriers of Migration” consistently in both the main heading and subsections.<br>- We added a new section, 3.3.3. In this section, we constructed a new composite variable for both drivers and obstacles, and then we assessed the impact of socioeconomic variables on these new variables. Please see section 3.3.3 and tables 5 and 6.

neutral, disagree – by making it binary may be possible for using binary logistic regression. If not, it is not exactly ordinal variable so author may use multinomial using "neutral" as reference category. OR author needs to find out an appropriate method how they can be measured statistically.	
Analysis and findings are not well connected with theoretical framework. Analysis has to be guided by the theoretical framework. For this, authors need to rework.	We appreciate the reviewer's valuable comment. In the revised manuscript, the Results and Discussion section has been restructured to ensure stronger alignment between the empirical analysis and the Protection Motivation Theory (PMT).
Conclusion is quite a bit long. However, it too is not connected with theoretical framework. It has to be well discussed whether the established theoretical foundation is sufficiently explain the migration intention or not.	We thank the reviewer for this valuable comment. In the revised manuscript, we shortened the Conclusion to make it more concise and focused. Importantly, we also restructured it to explicitly connect the findings with the Protection Motivation Theory (PMT). The revised conclusion now discusses how threat appraisal (land degradation, livelihood risks) and coping appraisal (education, remittances, assets) together explain migration intentions. It further highlights that PMT provides a suitable and sufficient theoretical foundation for understanding environmentally induced migration in the Fayoum context, while also pointing to areas for future research.
• In conclusion, the title of the paper does not seem to be well proven and the paper needs major revision.	We appreciate your suggestion. We made every effort to improve the manuscript overall.
Comments reviewer II	***Author's response***
Title: Consider making it more specific by including "Egypt" as the country case study	We appreciate the reviewer's valuable comment. Therefore, we modified the title. See the revised version of the manuscript.
**Abstract**: Remove redundancy (e.g., repeating "migration willingness") and clarify the novel contribution.	Many thanks for your suggestion, we considered that in the revised version.
**Introduction:** Better delineate the knowledge gap the paper addresses. Please clarify better how this study extends existing literature beyond identifying the problem.	We agree with your suggestion; therefore, we have considered it in the revised version, as shown in the modified introduction section. See below:- The novelty of the current work could be highlighted through the following points: (1) our focus is to analyze how the interaction between socioeconomic characteristics and environmental

Eg….While existing research has explored the relationship between economic factors and migration, few studies have specifically analyzed how ALD influences migrants' decisions, particularly in terms of migration timing. [Reference these works] Most prior<br>research has focused on general migration drivers, whereas our study sheds light on the<br>more specific…[Reference these works]	change leads to migration intention in developing countries based on primary data. We apply a construct framework, a combination of the principles of the Protection Motivation Theory (PMT) and multinomial logistic model, to predict the willingness to migrate under the potential ALD, which plays a significant role in increasing migration streams in developing countries. PMT is a novel and creative theoretical lens for migration; we consider complementing PMT with the livelihoods vulnerability framework and/or Push-Pull models to enhance explanatory power. While existing research has explored the relationship between economic factors and migration [23], few studies have specifically analyzed how ALD influences migrants' decisions, particularly in terms of migration timing [1,3,4,8]. Most prior research has focused on general migration drivers [24,25], whereas our study sheds light on the more specific aspects of the role of climate change to determine when the migrants will move from one region to another based on the degree of soil fertility and water availability in their origin rural area. (2) At the same time, we used the constructed framework to develop evidence-based recommendations about the specific push regions grounded on the degree of access to climate change mitigation and adaptation tools in rural areas in Egypt, which could contribute to future migration-reduction interventions. The result of the current study could best inform policymakers' and international donors' understanding of the environmental migration dynamics to design a strategy for addressing environmentally induced migration resulting from soil and land degradation in developing countries.
**Literature Review:** Whilst the literature engages with a broad set of sources from multiple geographies, it is too descriptive. More analytical synthesis is needed—compare, contrast, and critique existing works. Include more regionally comparative insights, especially from other North African or Middle Eastern contexts.	We thank the reviewer for this valuable comment. Therefore, we revised this section, highlighting the comparisons and critiques of the existing literature. Please check the revised version of the manuscript.
**5. Theoretical Framework:** PMT is a novel and creative theoretical lens for migration, but Better justify why PMT is appropriate in this migration context; originally used in health behavior studies. Consider complementing PMT with	We thank the reviewer for this constructive suggestion. We agree with you; therefore, we rewrote this section considering your suggestion. Please see the modified version.

Livelihoods Vulnerability Framework or Push-Pull models to enhance explanatory power.	
**6. Methodology:** More detail is needed on how variables were operationalized (e.g., how is "migration intention" coded?). Consider adding a table summarizing variable definitions and expected relationships. Explain potential biases and limitations, e.g., is Itsa District representative of broader Egypt? Include details on ethical clearance and informed consent.	We agree with you; therefore, we rewrote the methodology section, considering your suggestion.<br>We also added the table summarizing the variable definitions and expected relationships.<br>We agree that Itsa district isn't representative of broader Egypt, we explained that in the modified manuscript.<br>Please check the modified manuscript.
**Results and Discussion:** Well-structured and includes strong descriptive and inferential statistics.	Many thanks for your support. We appreciate that.
**Writing and Language:** The manuscript needs significant language polishing. Some sentences are overly long or grammatically incorrect. Examples: "the capital Cairo (40.2%) and international destinations (29.4%) were the most desired." → "Among those willing to migrate, Cairo (40.2%) and international destinations (29.4%) were preferred." Use of commas, conjunctions, and passive voice should be corrected throughout.	We apologize for the grammatical mistakes. We checked the language across the whole manuscript. Please see the modified manuscript.
**Policy and Practical Implications:** The discussion on policy implications is promising but too brief and generic. Expand on Targeted adaptation strategies, Links to SDGs, especially SDG 13 (Climate Action) and SDG 10 (Reduced Inequalities), Role of government, NGOs, and international donors in mitigating ALD.	We sincerely appreciate this suggestion; therefore, we modified this section according to your suggestion, considering the role of public-private partnership and linking with the SDGs 10 and 13. Please see the revised version.
**Conclusion:** Whilst the conclusion summarizes findings clearly, consider distilling 2–3 key takeaways for policymakers and academics. Include a brief note on future research directions, e.g., longitudinal studies or gender-specific analyses.	Many thanks for your suggestion. We added the limitations of the study and the future research direction based on the current findings.
Details about changes in the original manuscript included by the authors In addition to the reviewer's suggestion	

Section	*Changes*
**Abstract**	- We modified according to the two reviewer comments.
**Introduction**<br>- **Theoretical Framework**	- Added acknowledgment of Everett Lee (1966) and Ravenstein (1885) as the foundational migration theories. (pp 217-222)<br>- Expanded explanation to explicitly connect Figure 3 to the PMT. (pp 257-265)
**Material and methods**<br>- **Study Area and Sampling Strategy**	- Fayoum Governorate is often described as a microcosm of Egypt due to its geographical, agricultural, and demographic characteristics. It forms a closed depression that depends mainly on the Bahr Youssef canal as its primary water source, which resembles the role of the Nile River for Egypt. Its waters discharge into Lake Qarun and Wadi El-Rayan, which resemble Egypt's Mediterranean outlets. Within Fayoum, Itsa district was selected as the study area for several reasons: it is the largest district in terms of both area and population (708,780 people, about 20% of the governorate's population in 2017), and many of its villages are well known as sources of migration, such as Tatoun (to Italy) and Shidmu (to France).<br>To ensure representativeness, a **cluster sampling strategy** was adopted. The twelve local units of Itsa district were divided into **four geographically contiguous clusters**, each forming a sub-region within the district. Sample sizes in each cluster were calculated using **Thompson's formula** and cross-checked with **Krejcie and Morgan's (1970) sampling tables** to meet minimum requirements.<br>**Cluster 1 (Desert Hinterland):** Villages of Qalhana, Qolamsha, Qasr El-Basil, and Tatoun (population 166,878; sample size drawn = 431 > 383 minimum).<br>**Cluster 2 (Bahr Youssef Delta):** Villages of Gerdo, Matoul, and Abousir (population 197,868; sample size drawn = 488 > 384 minimum).<br>**Cluster 3 (Bahr El-Nazla and Bahr El-Banat lands):** Villages of Abogendeer and Menyet El-Heet (population 183,884; sample size drawn = 430 > 384 minimum). This cluster contains the most fertile lands.<br>**Cluster 4 (El-Gharq El-Soltany Basin):** Villages of El-Gharq, El-Hagar, and Anak (population 183,884; sample size drawn = 391 > 384 minimum).<br>In total, **1,782 households** were surveyed. One adult respondent was randomly selected from each sampled household. The overall sample size was also cross-

	validated using **Cochran's formula (1977)** at a 95% confidence level and 5% margin of error, and adjusted for potential non-responses. This multi-stage approach (cluster sampling followed by systematic random household selection within each cluster) ensures that the sample adequately represents the demographic and agricultural diversity of the district, making the findings generalizable to the broader rural population of Fayoum Governorate."<br><br>- Fayoum is one of the main exporters of both internal and international migrants in Egypt. The governorate is predominantly rural, with about 26.4% of its population living below the poverty line in 2019. (pp 269-271)
**Result and Discussion**<br>**Section: 3. Results and Discussion**<br><br><br>**3.2.5. Drivers and Barriers of Migration**<br><br><br>**Section: 3. Results and Discussion**	Reorganized subsection order.<br><br><br>Heading/Subheading Consistency: replaced "obstacles" with "barriers"<br><br><br>We added a paragraph before the Results to make it crystal clear that the analysis is theory-guided.<br>We also added a paragraph at the end of the Results " Overall, the analysis of migration intentions in Fayoum can be clearly understood within the Protection Motivation Theory (PMT) framework. Agricultural land degradation significantly increases threat appraisal by raising farmers' perceived severity and vulnerability, as declining productivity and livelihood insecurity heighten the risk of staying. At the same time, socio-economic resources such as education, remittances, and land assets serve as coping appraisal factors, shaping households' capacity to adapt locally or migrate successfully. The identified drivers of migration—such as environmental stress and unemployment—correspond to dimensions of threat appraisal, while the barriers—including limited resources, family obligations, and lack of skills—reflect coping constraints. Taken together, these findings demonstrate that migration decisions emerge from the interaction between threat and coping appraisals. This provides an integrated explanation of why

	some households choose to migrate while others remain, underscoring the value of PMT as a guiding theoretical framework for analyzing environmentally induced migration in developing-country contexts”
**Conclusion and recommendations**	The complex interrelationship between population dynamics, agricultural systems, and environmental change creates significant challenges for understanding the drivers of migration. Within the Protection Motivation Theory (PMT) framework, our findings show that threat appraisal is heightened by factors such as declining land productivity, inadequate rural services, and perceptions of economic insecurity, all of which increase the intention to migrate. At the same time, coping appraisal is shaped by socio-economic resources including education, remittances, and household assets, which determine whether individuals pursue migration or adapt locally. Demographic and social factors—particularly age, gender, and education—play a central role, with younger and better-educated individuals, especially women, more likely to migrate in search of autonomy and opportunity.<br>While absolute income levels were significant, perceptions of income adequacy exerted a more complex influence, suggesting that subjective well-being is a critical element in migration decisions. These findings confirm that PMT provides a sufficient and robust theoretical foundation for explaining migration intentions in rural Egypt, while also pointing to the value of integrating socio-economic and institutional perspectives for a fuller understanding.<br>Policy implications therefore include reducing threat appraisal through sustainable land and water management, poverty alleviation, and improved rural services, while strengthening coping appraisal through education, vocational training, and support for women’s entrepreneurship. Such interventions would not only address migration pressures but also contribute to inclusive and sustainable rural development.

Date: 12/10/2025
