# Supplementary material for: Agricultural land degradation consequences as a migration driver in Egypt
Source: PLoS One. 2026 Jul 17;21(7):e0353721. doi: 10.1371/journal.pone.0353721 (PMC13379038; doi:10.1371/journal.pone.0353721)
Supplement: Attachment — Submitted filename: PONE-D-25-21257 - Review.docx [file pone.0353721.s005.docx]

Agricultural Land Degradation Consequences as a Migration Driver in Developing Countries

No. PONE-D-25-21257

Comments:

- Conceptualization of the paper begins with push-pull theory of Everett Lee (1966) and basis of which is “laws of migration” Revenstein (1885). Therefore, it would be better if we acknowledge their prioneering contribution of the knowledgebase.
- Page 10 - “Figure 3. Migration intention under the agriculture land degredation” is not well connected with protection motivation theory (PMT). It needs more explanation in the text.
- Page 10 – Section 2.1 Study area and sampling strategy: The paper is silent about sample size (1782) how it has been calculated and whether it is representative or not for a particular population. It needs to be well established that the method of calculation and its representativeness.
- In the same page, line 254-56 is not clear.
- Page 11, line 277-78 – it says, “we selected the respondents randomly among the twelve rural villages in Itsa district”. It is not clear that whether it is individual survey or household survey. How was the sample from 12 villages randomly selected? Is this a cluster sampling? Or are these all 12 villages put together in a single sampling unit? They have to be clearly spelled out how the sample is random.
- Section 3.3. “Model fitting and pseudo R square”: It would be better to bring this section at the begging of the section 3 just before table 1. Then, go for the parameter estimates.
- It is confusing that table 1 displays both coefficients (b) and odds ratios (exp(b)) and level of significance is indicated in coefficients, however, the analysis has been made using exp(b). So, be clear and consistent between text and table.
- Section 3.2.5 Drivers and Barriers of migration: This is main heading, whereas in the subsection B, it is “obstacles”. Be consistent.
  - Author has to establish those questions included to inform drivers and barriers are valid and they would well represent the drivers and barriers.
  - Analysis of drivers and barriers of migration is weak. Authors need to find out the appropriate advance statistical method for measuring the drivers and barriers. They should be measured against socio-economic variables so that variation in drivers and barriers could be informed. Indexing into a single composite variable for bother drivers and obstacles may one option. And, retreatment of three level of scales – agree, neutral, disagree – by making it binary may be possible for using binary logistic regression. If not, it is not exactly ordinal variable so author may use multinomial using “neutral” as reference category. OR author needs to find out an appropriate method how they can be measured statistically.
- Analysis and findings are not well connected with theoretical framework. Analysis has to be guided by the theoretical framework. For this, authors need to rework.
- Conclusion is quite a bit long. However, it too is not connected with theoretical framework. It has to be well discussed whether the established theoretical foundation is sufficiently explain the migration intention or not.
- In conclusion, the title of the paper does not seem to be well proven and the paper needs major revision.
