# Supplementary material for: Agricultural land degradation consequences as a migration driver in Egypt
Source: PLoS One. 2026 Jul 17;21(7):e0353721. doi: 10.1371/journal.pone.0353721 (PMC13379038; doi:10.1371/journal.pone.0353721)
Supplement: Attachment — Submitted filename: 2Response-to-Reviewer-Comments-Form-PONE-D-25-21257.docx [file pone.0353721.s008.docx]

**Response to Reviewer Comments Form**

**FOR Plos One**

**Name of author(s):** Muhammad Hussien, Abdelrahman Ali, Yanwen Tan, Mohamed Abdelhameed

**Manuscript ID:** PONE-D-25-21257

**Title: “**Agricultural Land Degradation Consequences as a Migration Driver in Egypt”

| ***Comments reviewer I*** | ***Author’s response*** |
| --- | --- |
| Conceptualization of the paper begins with push-pull theory of Everett Lee (1966) and basis of which is “laws of migration” Revenstein (1885). Therefore, it would be better if we acknowledge their prioneering contribution of the knowledgebase. | We sincerely thank the reviewer for this valuable observation. We agree that the conceptualization of migration studies is deeply rooted in Everett Lee’s Push-Pull Theory (1966) and Ravenstein’s “Laws of Migration” (1885). We have therefore revised the introduction to explicitly acknowledge their pioneering contributions and to situate our study within this historical foundation. See the revised version of the manuscript. |
| Page 10 - “Figure 3. Migration intention under the agriculture land degredation” is not well connected with protection motivation theory (PMT). It needs more explanation in the text. | We thank the reviewer for pointing this out. We agree that Figure 3 (“Migration intention under the agricultural land degradation”) required a stronger linkage to the Protection Motivation Theory (PMT). We revised the manuscript by adding explanatory text directly before and after Figure 3 to clarify how PMT constructs (threat appraisal and coping appraisal) are reflected in the figure. This ensures that the theoretical framework and empirical findings are conceptually aligned. |
| Page 10 – Section 2.1 Study area and sampling strategy: The paper is silent about sample size (1782) how it has been calculated and whether it is representative or not for a particular population. It needs to be well established that the method of calculation and its representativeness. | We thank the reviewer for raising this point. In the revised manuscript, we clarified that the sample size of 1,782 households was determined using Cochran’s formula (1977) at a 95% confidence level and 5% margin of error, adjusted for Fayoum’s rural population. A cluster sampling approach was applied across the 12 villages of Itsa district, grouped into four clusters. Within each cluster, households were selected through systematic random sampling, with one adult respondent interviewed per household. This ensured statistical representativeness of the rural population. |
| In the same page, line 254-56 is not clear. | We thank the reviewer for pointing out this unclear sentence. We have revised it to improve clarity and readability. The revised version corrects the grammar and removes the confusing expression, while retaining the intended meaning about Fayoum’s significance as a migration-exporting governorate and its poverty level. |
| Page 11, line 277-78 – it says, “we selected the respondents randomly among the twelve rural villages in Itsa district”. It is not clear that whether it is individual survey or household survey. How was the sample from 12 villages randomly selected? Is this a cluster sampling? Or are these all 12 villages put together in a single sampling unit? They have to be clearly spelled out how the sample is random. | We thank the reviewer for this important observation. We have clarified in the revised manuscript that the survey was household-based and that one adult respondent was randomly selected from each sampled household. The 12 villages in Itsa district were treated as clusters, and within each village, households were selected using systematic random sampling from official village household lists provided by local authorities. This approach ensured both randomness and proportional representation of the selected villages. |
| Section 3.3. “Model fitting and pseudo R square”: It would be better to bring this section at the begging of the section 3 just before table 1. Then, go for the parameter estimates. | We thank the reviewer for this constructive suggestion. We agree that placing the subsection “Model fitting and pseudo R square” at the beginning of Section 3 improves the logical flow of results. Accordingly, we have moved this subsection to appear immediately before Table 1, followed by the parameter estimates. |
| It is confusing that table 1 displays both coefficients (b) and odds ratios (exp(b)) and level of significance is indicated in coefficients, however, the analysis has been made using exp(b). So, be clear and consistent between text and table. | We appreciate the reviewer’s careful reading of Table 2. We agree that the presentation was confusing because both coefficients (b) and odds ratios (Exp(b)) were reported, while the analysis and discussion were based on odds ratios. To ensure clarity and consistency, we have revised Table 2 to present only the odds ratios (Exp(b)) along with their corresponding significance levels. The text discussion has also been aligned to reference odds ratios consistently. |
| Section 3.2.5 Drivers and Barriers of migration: This is main heading, whereas in the subsection B, it is “obstacles”. Be consistent.   - Author has to establish those questions included to inform drivers and barriers are valid and they would well represent the drivers and barriers. - Analysis of drivers and barriers of migration is weak. Authors need to find out the appropriate advance statistical method for measuring the drivers and barriers. They should be measured against socio-economic variables so that variation in drivers and barriers could be informed. Indexing into a single composite variable for bother drivers and obstacles may one option. And, retreatment of three level of scales – agree, neutral, disagree – by making it binary may be possible for using binary logistic regression. If not, it is not exactly ordinal variable so author may use multinomial using “neutral” as reference category. OR author needs to find out an appropriate method how they can be measured statistically. | **We thank the reviewer for this comprehensive feedback. In response, we made three major revisions:**   - Terminology Consistency: We standardized the terminology by using “Drivers and Barriers of Migration” consistently in both the main heading and subsections. - We added a new section, 3.3.3. In this section, we constructed a new composite variable for both drivers and obstacles, and then we assessed the impact of socioeconomic variables on these new variables. Please see section 3.3.3 and tables 5 and 6. |
| Analysis and findings are not well connected with theoretical framework. Analysis has to be guided by the theoretical framework. For this, authors need to rework. | We appreciate the reviewer’s valuable comment. In the revised manuscript, the Results and Discussion section has been restructured to ensure stronger alignment between the empirical analysis and the Protection Motivation Theory (PMT). |
| Conclusion is quite a bit long. However, it too is not connected with theoretical framework. It has to be well discussed whether the established theoretical foundation is sufficiently explain the migration intention or not. | We thank the reviewer for this valuable comment. In the revised manuscript, we shortened the Conclusion to make it more concise and focused. Importantly, we also restructured it to explicitly connect the findings with the Protection Motivation Theory (PMT). The revised conclusion now discusses how threat appraisal (land degradation, livelihood risks) and coping appraisal (education, remittances, assets) together explain migration intentions. It further highlights that PMT provides a suitable and sufficient theoretical foundation for understanding environmentally induced migration in the Fayoum context, while also pointing to areas for future research. |
| • In conclusion, the title of the paper does not seem to be well proven and the paper needs major revision. | We appreciate your suggestion. We made every effort to improve the manuscript overall. |
|  |  |
| ***Comments reviewer II*** | ***Author’s response*** |
| Title: Consider making it more specific by including “Egypt” as the country case study | We appreciate the reviewer’s valuable comment. Therefore, we modified the title. See the revised version of the manuscript. |
| **Abstract**: Remove redundancy (e.g., repeating “migration willingness”) and clarify the  novel contribution. | Many thanks for your suggestion, we considered that in the revised version. |
| **Introduction:** Better delineate the knowledge gap the paper addresses. Please clarify better how this study extends existing literature beyond identifying the problem.  Eg….While existing research has explored the relationship between economic factors and migration, few studies have specifically analyzed how ALD influences migrants'  decisions, particularly in terms of migration timing. [Reference these works] Most prior  research has focused on general migration drivers, whereas our study sheds light on the  more specific…[Reference these works] | We agree with your suggestion; therefore, we have considered it in the revised version, as shown in the modified introduction section. See below:-  The novelty of the current work could be highlighted through the following points: (1) our focus is to analyze how the interaction between socioeconomic characteristics and environmental change leads to migration intention in developing countries based on primary data. We apply a construct framework, a combination of the principles of the Protection Motivation Theory (PMT) and multinomial logistic model, to predict the willingness to migrate under the potential ALD, which plays a significant role in increasing migration streams in developing countries. PMT is a novel and creative theoretical lens for migration; we consider complementing PMT with the livelihoods vulnerability framework and/or Push-Pull models to enhance explanatory power. While existing research has explored the relationship between economic factors and migration [23], few studies have specifically analyzed how ALD influences migrants' decisions, particularly in terms of migration timing [1,3,4,8]. Most prior research has focused on general migration drivers [24,25], whereas our study sheds light on the more specific aspects of the role of climate change to determine when the migrants will move from one region to another based on the degree of soil fertility and water availability in their origin rural area. (2) At the same time, we used the constructed framework to develop evidence-based recommendations about the specific push regions grounded on the degree of access to climate change mitigation and adaptation tools in rural areas in Egypt, which could contribute to future migration-reduction interventions. The result of the current study could best inform policymakers' and international donors' understanding of the environmental migration dynamics to design a strategy for addressing environmentally induced migration resulting from soil and land degradation in developing countries. |
| **Literature Review:** Whilst the literature engages with a broad set of sources from multiple geographies, it is too descriptive. More analytical synthesis is needed—compare, contrast, and critique existing works. Include more regionally comparative insights, especially from other North African or Middle Eastern contexts. | We thank the reviewer for this valuable comment. Therefore, we revised this section, highlighting the comparisons and critiques of the existing literature. Please check the revised version of the manuscript. |
| **5. Theoretical Framework:** PMT is a novel and creative theoretical lens for migration, but Better justify why PMT is appropriate in this migration context; originally used in health behavior studies. Consider complementing PMT with Livelihoods Vulnerability Framework or Push-Pull models to enhance explanatory power. | We thank the reviewer for this constructive suggestion. We agree with you; therefore, we rewrote this section considering your suggestion. Please see the modified version. |
| **6. Methodology:** More detail is needed on how variables were operationalized (e.g., how is "migration intention" coded?). Consider adding a table summarizing variable  definitions and expected relationships. Explain potential biases and limitations, e.g., is Itsa District representative of broader Egypt? Include details on ethical clearance and  informed consent. | We agree with you; therefore, we rewrote the methodology section, considering your suggestion.  We also added the table summarizing the variable  definitions and expected relationships.  We agree that Itsa district isn’t representative of broader Egypt, we explained that in the modified manuscript.  Please check the modified manuscript. |
| **Results and Discussion:** Well-structured and includes strong descriptive and inferential statistics. | Many thanks for your support. We appreciate that. |
| **Writing and Language:** The manuscript needs significant language polishing. Some sentences are overly long or grammatically incorrect. Examples: “the capital Cairo (40.2%) and international destinations (29.4%) were the most desired.” → “Among those willing to migrate, Cairo (40.2%) and international destinations (29.4%) were preferred.” Use of commas, conjunctions, and passive voice should be corrected throughout. | We apologize for the grammatical mistakes. We checked the language across the whole manuscript. Please see the modified manuscript. |
| **Policy and Practical Implications:** The discussion on policy implications is promising but too brief and generic. Expand on Targeted adaptation strategies, Links to SDGs,  especially SDG 13 (Climate Action) and SDG 10 (Reduced Inequalities), Role of government, NGOs, and international donors in mitigating ALD. | We sincerely appreciate this suggestion; therefore, we modified this section according to your suggestion, considering the role of public-private partnership and linking with the SDGs 10 and 13. Please see the revised version. |
| **Conclusion:** Whilst the conclusion summarizes findings clearly, consider distilling 2–3 key takeaways for policymakers and academics. Include a brief note on future research directions, e.g., longitudinal studies or gender-specific analyses. | Many thanks for your suggestion. We added the limitations of the study and the future research direction based on the current findings. |
| ***Details about changes in the original manuscript included by the authors***  ***In addition to the reviewer's suggestion*** | |
| ***Section*** | ***Changes*** |
| **Abstract** | - We modified according to the two reviewer comments. |
| **Introduction**   - **Theoretical Framework** | - Added acknowledgment of Everett Lee (1966) and Ravenstein (1885) as the foundational migration theories. (pp 217-222) - Expanded explanation to explicitly connect Figure 3 to the PMT. (pp 257-265) |
| **Material and methods**   - **Study Area and Sampling Strategy** | - Fayoum Governorate is often described as a microcosm of Egypt due to its geographical, agricultural, and demographic characteristics. It forms a closed depression that depends mainly on the Bahr Youssef canal as its primary water source, which resembles the role of the Nile River for Egypt. Its waters discharge into Lake Qarun and Wadi El-Rayan, which resemble Egypt’s Mediterranean outlets. Within Fayoum, Itsa district was selected as the study area for several reasons: it is the largest district in terms of both area and population (708,780 people, about 20% of the governorate’s population in 2017), and many of its villages are well known as sources of migration, such as Tatoun (to Italy) and Shidmu (to France).   To ensure representativeness, a **cluster sampling strategy** was adopted. The twelve local units of Itsa district were divided into **four geographically contiguous clusters**, each forming a sub-region within the district. Sample sizes in each cluster were calculated using **Thompson’s formula** and cross-checked with **Krejcie and Morgan’s (1970) sampling tables** to meet minimum requirements.  **Cluster 1 (Desert Hinterland):** Villages of Qalhana, Qolamsha, Qasr El-Basil, and Tatoun (population 166,878; sample size drawn = 431 > 383 minimum).  **Cluster 2 (Bahr Youssef Delta):** Villages of Gerdo, Matoul, and Abousir (population 197,868; sample size drawn = 488 > 384 minimum).  **Cluster 3 (Bahr El-Nazla and Bahr El-Banat lands):** Villages of Abogendeer and Menyet El-Heet (population 183,884; sample size drawn = 430 > 384 minimum). This cluster contains the most fertile lands.  **Cluster 4 (El-Gharq El-Soltany Basin):** Villages of El-Gharq, El-Hagar, and Anak (population 183,884; sample size drawn = 391 > 384 minimum).  In total, **1,782 households** were surveyed. One adult respondent was randomly selected from each sampled household. The overall sample size was also cross-validated using **Cochran’s formula (1977)** at a 95% confidence level and 5% margin of error, and adjusted for potential non-responses. This multi-stage approach (cluster sampling followed by systematic random household selection within each cluster) ensures that the sample adequately represents the demographic and agricultural diversity of the district, making the findings generalizable to the broader rural population of Fayoum Governorate.”   - Fayoum is one of the main exporters of both internal and international migrants in Egypt. The governorate is predominantly rural, with about 26.4% of its population living below the poverty line in 2019. (pp 269-271) |
| **Result and Discussion**  **Section: 3. Results and Discussion**  3.2.5. Drivers and Barriers of Migration  **Section: 3. Results and Discussion** | Reorganized subsection order.  Heading/Subheading Consistency: replaced “obstacles” with “barriers”  We added a paragraph before the Results to make it crystal clear that the analysis is theory-guided.  We also added a paragraph at the end of the Results " Overall, the analysis of migration intentions in Fayoum can be clearly understood within the Protection Motivation Theory (PMT) framework. Agricultural land degradation significantly increases threat appraisal by raising farmers’ perceived severity and vulnerability, as declining productivity and livelihood insecurity heighten the risk of staying. At the same time, socio-economic resources such as education, remittances, and land assets serve as coping appraisal factors, shaping households’ capacity to adapt locally or migrate successfully. The identified drivers of migration—such as environmental stress and unemployment—correspond to dimensions of threat appraisal, while the barriers—including limited resources, family obligations, and lack of skills—reflect coping constraints. Taken together, these findings demonstrate that migration decisions emerge from the interaction between threat and coping appraisals. This provides an integrated explanation of why some households choose to migrate while others remain, underscoring the value of PMT as a guiding theoretical framework for analyzing environmentally induced migration in developing-country contexts” |
| **Conclusion and recommendations** | The complex interrelationship between population dynamics, agricultural systems, and environmental change creates significant challenges for understanding the drivers of migration. Within the Protection Motivation Theory (PMT) framework, our findings show that threat appraisal is heightened by factors such as declining land productivity, inadequate rural services, and perceptions of economic insecurity, all of which increase the intention to migrate. At the same time, coping appraisal is shaped by socio-economic resources including education, remittances, and household assets, which determine whether individuals pursue migration or adapt locally. Demographic and social factors—particularly age, gender, and education—play a central role, with younger and better-educated individuals, especially women, more likely to migrate in search of autonomy and opportunity.  While absolute income levels were significant, perceptions of income adequacy exerted a more complex influence, suggesting that subjective well-being is a critical element in migration decisions. These findings confirm that PMT provides a sufficient and robust theoretical foundation for explaining migration intentions in rural Egypt, while also pointing to the value of integrating socio-economic and institutional perspectives for a fuller understanding.  Policy implications therefore include reducing threat appraisal through sustainable land and water management, poverty alleviation, and improved rural services, while strengthening coping appraisal through education, vocational training, and support for women’s entrepreneurship. Such interventions would not only address migration pressures but also contribute to inclusive and sustainable rural development. |
|  |  |

**Date: 12/10/2025**
