# Supplementary material for: Agricultural land degradation consequences as a migration driver in Egypt
Source: PLoS One. 2026 Jul 17;21(7):e0353721. doi: 10.1371/journal.pone.0353721 (PMC13379038; doi:10.1371/journal.pone.0353721)
Supplement: Attachment — Submitted filename: R2Response-to-Reviewer-Comments-Form-PONE-D-25-21257R1.docx [file pone.0353721.s009.docx]

**Response to Reviewer Comments Form**

**FOR Plos One**

**Name of author(s):** Muhammad Hussien, Abdelrahman Ali, Yanwen Tan, Mohamed Abdelhameed

**Manuscript ID:** PONE-D-25-21257**R1**

**Title: “**Agricultural Land Degradation Consequences as a Migration Driver in Egypt”

| ***Comments reviewer 3*** | ***Author’s response*** |
| --- | --- |
| - overloading words, reduce too much writing  - the ALP should be logic 1 in the analysis since it is the main determinant variable | We sincerely thank the reviewer for this valuable observation. We agree that the manuscript requires a reduction in word count. We have therefore reduced the introduction and review sections to explicitly acknowledge their pioneering contributions and to situate our study within this historical foundation. See the revised version of the manuscript. |
|  |  |
| ***Comments reviewer II*** | ***Author’s response*** |
| Conceptual and Theoretical Framework  The manuscript introduces Protection Motivation Theory (PMT) as the central theoretical framework and attempts to combine it with push–pull theory and livelihood approaches. While this is potentially innovative, the integration remains superficial. The key constructs of PMT—such as threat appraisal and coping appraisal—are not clearly operationalized into measurable variables, nor are they empirically tested within the model. As a result, PMT functions more as a descriptive narrative rather than an analytical framework. Additionally, the simultaneous use of multiple theoretical perspectives without clear differentiation leads to conceptual overlap and weakens the overall coherence of the study. A more focused and explicitly operationalized theoretical framework is necessary. | We appreciate the reviewer’s valuable comments. Many thanks for your suggestion, we considered that in the revised version.  We just mentioned the push–pull theory and livelihood approaches to introduce the PMT. We want to draw your attention to the fact that the PMT is no longer presented merely as a descriptive narrative, but rather as the primary analytical framework for interpreting migration intention in the context of agricultural land degradation (ALD). See lines 222-227.  Specifically, the manuscript now explicitly operationalizes the two core dimensions of PMT (threat appraisal and coping appraisal) within the migration context. Threat appraisal is conceptualized through farmers’ perceived severity and vulnerability to ALD, including declining agricultural productivity, income loss, livelihood insecurity, and socioeconomic stress. Coping appraisal is linked to migrants’ perceived adaptive capacity, including access to financial resources, migration networks, legal opportunities, skills, and confidence in successful relocation. The revised text further explains that migration intention emerges from the interaction between heightened threat appraisal and varying levels of coping appraisal.  To address concerns regarding conceptual overlap, the revised framework also clarifies the distinct role of each theoretical perspective. PMT serves as the central behavioral decision-making framework explaining how individuals cognitively evaluate environmental threats and adaptation responses. In contrast, Push–Pull Theory is used only to contextualize broader structural drivers of migration (e.g., environmental stress as push factors and urban/economic opportunities as pull factors), whereas the livelihoods vulnerability perspective provides background on socioeconomic sensitivity and adaptive constraints. These approaches are therefore complementary rather than competing analytical models.  Furthermore, Figures 2 and 3 were revised and explicitly linked to the PMT dimensions to demonstrate that ALD-induced environmental stress increases threat appraisal, whereas migration feasibility depends on coping appraisal. This revision improves the coherence of the theoretical framework and strengthens the analytical integration between environmental change, vulnerability, and migration decision-making. |
| Methodology and Data  The study employs a multinomial logistic regression model, which is appropriate given the categorical nature of migration intention. The sampling strategy is described as multi-stage cluster sampling within Fayoum Governorate. However, the generalizability of the findings is overstated, as the analysis is based on a single district (Itsa), which may not represent broader national dynamics . Furthermore, key variables such as agricultural land degradation are measured through self-reported perceptions rather than objective environmental indicators, raising concerns about measurement bias.  The model specification lacks sufficient rigor. There is no discussion of important assumptions such as independence of irrelevant alternatives (IIA), nor are there diagnostics for multicollinearity or robustness checks. Although the model fit statistics are reported, the explanatory power remains moderate (Nagelkerke R² ≈ 0.278) , and this limitation is not adequately discussed. | We agree with your suggestion; therefore, we have considered it in the revised version. See below:-  Several clarifications have been added to better justify the study design, acknowledge limitations, and strengthen the paper's methodological transparency.  First, regarding generalizability, the revised manuscript clarifies that the findings should be interpreted primarily within the context of Fayoum Governorate and, more specifically, *Itsa district*, rather than as fully representative of all rural areas in Egypt. Nevertheless, Fayoum was intentionally selected because it is widely considered a microcosm of Egypt in terms of agricultural dependence, rural poverty, water scarcity, and migration patterns. Within Fayoum, *Itsa district* was chosen because it is the largest in terms of population and agricultural activity, and is particularly vulnerable to downstream water shortages and land degradation. The multi-stage cluster sampling design, combined with a large sample size of 1,782 households, was intended to maximize representativeness within the selected study area. The revised text now presents the study as a context-specific case study with broader analytical relevance rather than claiming full national generalizability.  Second, the reviewer’s concern about measuring agricultural land degradation (ALD) through self-reported perceptions is well taken. The manuscript now explicitly acknowledges this limitation. The study primarily focuses on farmers’ perceived experiences of environmental stress, as migration intention is strongly influenced by subjective risk perception and livelihood insecurity, which align with the Protection Motivation Theory (PMT). Variables such as perceived changes in land productivity, cultivated area, village conditions, and living conditions over the previous ten years were therefore used as proxies for ALD-related stress. However, the revised manuscript now recognizes that the inclusion of objective environmental indicators (e.g., soil salinity measurements, remote sensing data, or land productivity records) would further strengthen future research and reduce potential measurement bias.  Third, additional clarification has been added regarding the multinomial logistic regression (MLR) specification. The manuscript now explains that the MLR model was selected because the dependent variable consists of three unordered categories of migration intention (“No”, “Maybe”, and “Yes”). Model fit statistics, including the likelihood ratio test and Nagelkerke R², were reported to assess explanatory performance. The revised discussion now explicitly acknowledges that the model's explanatory power is moderate (Nagelkerke R² ≈ 0.278), reflecting the complexity of migration decision-making, which is influenced by many unobservable psychological, institutional, and contextual factors not fully captured in cross-sectional survey data.  In response to the reviewer’s methodological concerns, the revised manuscript also adds a statement acknowledging that some diagnostic procedures, including formal testing of the Independence of Irrelevant Alternatives (IIA) assumption and robustness checks for multicollinearity, were not fully explored in the original analysis and should be considered in future studies to strengthen model reliability further. Nonetheless, efforts were made to reduce specification problems by carefully selecting explanatory variables based on theoretical relevance and prior migration literature, while avoiding excessive redundancy among predictors. |
| **Literature Review:** Whilst the literature engages with a broad set of sources from multiple geographies, it is too descriptive. More analytical synthesis is needed—compare, contrast, and critique existing works. Include more regionally comparative insights, especially from other North African or Middle Eastern contexts. | We thank the reviewer for this valuable comment. Therefore, we revised this section, highlighting the comparisons and critiques of the existing literature. Please check the revised version of the manuscript. |
| **Results and Interpretation**  The results section presents statistically significant findings regarding demographic and socioeconomic determinants of migration intention. However, the interpretation often overreaches by implying causal relationships, despite the cross-sectional design of the study. The linkage between empirical findings and PMT remains weak, as coefficients are not clearly mapped onto theoretical constructs. Additionally, the analysis lacks depth, as it does not explore interaction effects or subgroup heterogeneity (e.g., gender, land ownership, or income levels), which could provide more nuanced insights into migration dynamics. | We thank the reviewer for this constructive suggestion. We agree with you; therefore, we have adjusted this section in accordance with your suggestion. Please see the modified version.  Several revisions have been made to improve analytical precision, avoid causal overinterpretation, and strengthen the theoretical integration of the findings.  First, the revised manuscript now avoids causal language throughout the Results and Discussion section. Because the study is based on cross-sectional survey data, the findings are interpreted as statistical associations rather than causal relationships. Terms such as “associated with,” “linked to,” and “related to” are now consistently used instead of implying direct causation. The revised text also explicitly acknowledges that migration intention is shaped by complex and dynamic socioeconomic and environmental processes that cannot be fully captured by cross-sectional analysis alone.  Second, the linkage between the empirical findings and PMT has been substantially strengthened. The revised results section now explicitly interprets the main explanatory variables through the two core PMT dimensions: threat appraisal and coping appraisal. For example, variables such as declining land productivity, worsening living conditions, inadequate income, and environmental stress are now discussed as indicators of heightened threat appraisal because they increase perceived vulnerability and severity associated with remaining in degraded rural areas. In contrast, variables such as education level, monthly income, migration networks, and employment opportunities are interpreted as coping appraisal factors because they reflect respondents’ perceived capacity to adapt successfully through migration. Similarly, barriers such as high moving costs, family obligations, and a lack of housing are discussed as coping constraints that reduce the feasibility of migration despite high threat perception.  The revised manuscript also improves the interpretation of specific coefficients within the PMT framework. For instance, younger and more educated respondents are interpreted as having higher coping appraisal because they possess greater mobility, skills, and awareness of external opportunities. Likewise, respondents experiencing declining agricultural productivity are interpreted as exhibiting stronger threat appraisal due to increased livelihood insecurity. These revisions ensure that the statistical findings are more systematically connected to the theoretical framework rather than presented as isolated empirical observations.  Third, the manuscript now explicitly acknowledges the limitations of the current analysis regarding interaction effects and subgroup heterogeneity. The reviewer’s suggestion concerning differences across gender, land ownership, and income groups is highly valuable. Due to scope and model complexity, the present study focused primarily on the main effects of socioeconomic and environmental variables. However, the revised discussion now recognizes that migration intentions may vary substantially across social groups and that interactions among variables such as gender, education, income adequacy, agricultural dependence, and land ownership could yield more nuanced insights into migration dynamics.  To partially address this concern, the revised interpretation places greater emphasis on subgroup patterns already evident in the results. For example, the analysis now highlights that women, younger respondents, highly educated individuals, and landless or low-income households exhibit different migration tendencies under environmental stress. Nevertheless, the manuscript clearly identifies the exploration of interaction effects and heterogeneity analysis as an important direction for future research, particularly through longitudinal or mixed-method approaches.  Finally, the discussion section has been revised to provide a more balanced interpretation of the model’s explanatory capacity. The moderate pseudo R² values are now explicitly acknowledged as expected in migration studies, where decisions are influenced by many unobservable institutional, psychological, cultural, and contextual factors beyond those included in the survey. |
| Writing, Structure, and Presentation  The manuscript suffers from issues related to language clarity, redundancy, and organization. Several sections, particularly the literature review are overly descriptive and repetitive, with limited critical synthesis. Transitions between sections (e.g., theory to methods, results to discussion) are not always smooth, affecting the overall readability. There are also frequent grammatical errors and stylistic inconsistencies that require professional language editing. Figures, particularly those illustrating the conceptual framework, are informative but visually dense and could be simplified for better clarity. | We agree with you. We apologize for the grammatical mistakes. We checked the language across the whole manuscript.  Therefore, we have revised the language for clarity, reduced redundancy, and improved organization. Please check the modified manuscript. |
| Ethical and Reporting Issues  The ethics statement provided in the manuscript is insufficient. The statement that the data were analyzed anonymously does not meet standard reporting requirements, as it lacks information on institutional ethical approval and consent procedures . This needs to be addressed to ensure compliance with journal guidelines. | Many thanks for your comment. We followed the national legislation, which indicates that we don't need ethical approval for this type of research because we didn't include clinical trials, human material, or human tissues. This is according to the (The Constitution of The Arab Republic of Egypt 1971), Part 3, Article 43, (Page 5 in the attached file). "Art.43: Any medical or scientific experiment may not be performed on any person without his free consent."  Downloaded from the official link of the World Intellectual Property Organization (WIPO). <https://www.wipo.int/wipolex/en/text/189854> |
| Conclusion and Recommendation  In conclusion, the manuscript addresses an important and policy-relevant issue and is supported by a substantial primary dataset. However, significant revisions are required to strengthen the theoretical grounding, improve methodological transparency, and enhance analytical rigor. The authors should focus on properly operationalizing PMT, refining the empirical strategy, incorporating robustness checks, and improving the clarity and coherence of the writing. | We thank the reviewer for this valuable comment. Therefore, we revised this section. We have substantially revised the paper to strengthen its theoretical grounding, improve methodological transparency, and enhance the study's analytical rigor.  First, the revised manuscript clarifies the role of the Protection Motivation Theory (PMT) as the central analytical framework rather than a descriptive narrative. The two principal PMT dimensions—threat appraisal and coping appraisal—have now been explicitly operationalized and systematically linked to the empirical analysis. Threat appraisal is represented through respondents’ perceptions of declining land productivity, livelihood insecurity, worsening living conditions, and environmental stress associated with agricultural land degradation. Coping appraisal is reflected through socioeconomic resources such as education, income adequacy, agricultural assets, migration opportunities, and adaptive capacity. The revised results and conclusions now consistently interpret migration intention through the interaction between these PMT dimensions.  Second, in response to concerns about analytical rigor, the revised manuscript provides a more balanced interpretation of the multinomial logistic regression results and explicitly avoids causal claims given the study's cross-sectional design. The moderate explanatory power of the models is now openly discussed as consistent with the multidimensional nature of migration decision-making. Furthermore, the manuscript acknowledges that formal robustness checks, including diagnostics for multicollinearity and tests of the Independence of Irrelevant Alternatives (IIA) assumption, were not fully explored and should be incorporated into future research to strengthen model validation.  The revised manuscript also improves the linkage between empirical findings and PMT by interpreting demographic, economic, and agricultural variables within the framework of threat and coping appraisal. For example, declining land productivity and inadequate income are now interpreted as factors that increase threat appraisal, while education, income adequacy, and access to livelihood opportunities are interpreted as factors that strengthen coping appraisal and migration feasibility. In addition, the revised discussion acknowledges the importance of subgroup heterogeneity and interaction effects across gender, education, income, and land ownership categories, which represent important directions for future research. |
|  |  |

**Date: 11/6/2026**
