# Supplementary material for: Agricultural land degradation consequences as a migration driver in Egypt
Source: PLoS One. 2026 Jul 17;21(7):e0353721. doi: 10.1371/journal.pone.0353721 (PMC13379038; doi:10.1371/journal.pone.0353721)
Supplement: Peer review history

# Peer review: Decision Letter 0

**DOI:** 10.1371/journal.pone.0353721.r001

**Authors:** Gouranga Dasvarma

6 Sep 2025

PONE-D-25-21257

Agricultural Land Degradation Consequences as a Migration Driver in Developing Countries

PLOS ONE

Dear Dr. Ali,

Thank you for submitting your manuscript to PLOS ONE. After careful consideration, we feel that it has merit but does not fully meet PLOS ONE’s publication criteria as it currently stands. Therefore, we invite you to submit a revised version of the manuscript that addresses the points raised during the review process.

Please submit your revised manuscript by Oct 21 2025 11:59PM. If you will need more time than this to complete your revisions, please reply to this message or contact the journal office at plosone@plos.org. When you're ready to submit your revision, log on to https://www.editorialmanager.com/pone/ and select the 'Submissions Needing Revision' folder to locate your manuscript file.

Please include the following items when submitting your revised manuscript:

- A rebuttal letter that responds to each point raised by the academic editor and reviewer(s). You should upload this letter as a separate file labelled 'Response to Reviewers'.
- A marked-up copy of your manuscript that highlights changes made to the original version. You should upload this as a separate file labelled 'Revised Manuscript with Track Changes'.
- An unmarked version of your revised paper without tracked changes. You should upload this as a separate file labelled 'Manuscript'.
- Please also have your manuscript edited for English before submitting the revision.

If you would like to make changes to your financial disclosure, please include your updated statement in your cover letter. Guidelines for resubmitting your figure files are available below the reviewer comments at the end of this letter.

If applicable, we recommend that you deposit your laboratory protocols in protocols.io to enhance the reproducibility of your results. Protocols.io assigns your protocol its own identifier (DOI) so that it can be cited independently in the future. For instructions see: https://journals.plos.org/plosone/s/submission-guidelines#loc-laboratory-protocols. Additionally, PLOS ONE offers an option for publishing peer-reviewed Lab Protocol articles, which describe protocols hosted on protocols.io. Read more information on sharing protocols at https://plos.org/protocols?utm_medium=editorial-email&utm_source=authorletters&utm_campaign=protocols.

We look forward to receiving your revised manuscript.

Kind regards,

Gouranga Lal Dasvarma, PhD

Academic Editor

PLOS ONE

Journal Requirements:

When submitting your revision, we need you to address these additional requirements.

1. Please ensure that your manuscript meets PLOS ONE's style requirements, including those for file naming. The PLOS ONE style templates can be found at https://journals.plos.org/plosone/s/file?id=wjVg/PLOSOne_formatting_sample_main_body.pdf and https://journals.plos.org/plosone/s/file?id=ba62/PLOSOne_formatting_sample_title_authors_affiliations.pdf

2. Please include your full ethics statement in the ‘Methods’ section of your manuscript file. In your statement, please include the full name of the IRB or ethics committee who approved or waived your study, as well as whether or not you obtained informed written or verbal consent. If consent was waived for your study, please include this information in your statement as well.

3. Please provide a complete Data Availability Statement in the submission form, ensuring you include all necessary access information or a reason for why you are unable to make your data freely accessible. If your research concerns only data provided within your submission, please write "All data are in the manuscript and/or supporting information files" as your Data Availability Statement.

4. Thank you for stating the following financial disclosure:

This work was supported by The National Natural Science Foundation of China Project: The Economic Transformation and the Development of Regional Agricultural Products Value Chain of ASEAN and China (71961147002).

Please state what role the funders took in the study. If the funders had no role, please state: "The funders had no role in study design, data collection and analysis, decision to publish, or preparation of the manuscript."

If this statement is not correct you must amend it as needed.

Please include this amended Role of Funder statement in your cover letter; we will change the online submission form on your behal

5. Please note that funding information should not appear in any section or other areas of your manuscript. We will only publish funding information present in the Funding Statement section of the online submission form. Please remove any funding-related text from the manuscript.

6. Your abstract cannot contain citations. Please only include citations in the body text of the manuscript, and ensure that they remain in ascending numerical order on first mention.

7. We note that Figure 4 in your submission contain [map/satellite] images which may be copyrighted. All PLOS content is published under the Creative Commons Attribution License (CC BY 4.0), which means that the manuscript, images, and Supporting Information files will be freely available online, and any third party is permitted to access, download, copy, distribute, and use these materials in any way, even commercially, with proper attribution. For these reasons, we cannot publish previously copyrighted maps or satellite images created using proprietary data, such as Google software (Google Maps, Street View, and Earth). For more information, see our copyright guidelines: http://journals.plos.org/plosone/s/licenses-and-copyright.

We require you to either (1) present written permission from the copyright holder to publish these figures specifically under the CC BY 4.0 license, or (2) remove the figures from your submission:

a. You may seek permission from the original copyright holder of Figure 4 to publish the content specifically under the CC BY 4.0 license.

We recommend that you contact the original copyright holder with the Content Permission Form (http://journals.plos.org/plosone/s/file?id=7c09/content-permission-form.pdf) and the following text:

“I request permission for the open-access journal PLOS ONE to publish XXX under the Creative Commons Attribution License (CCAL) CC BY 4.0 (http://creativecommons.org/licenses/by/4.0/). Please be aware that this license allows unrestricted use and distribution, even commercially, by third parties. Please reply and provide explicit written permission to publish XXX under a CC BY license and complete the attached form.”

Please upload the completed Content Permission Form or other proof of granted permissions as an "Other" file with your submission.

In the figure caption of the copyrighted figure, please include the following text: “Reprinted from [ref] under a CC BY license, with permission from [name of publisher], original copyright [original copyright year].”

b. If you are unable to obtain permission from the original copyright holder to publish these figures under the CC BY 4.0 license or if the copyright holder’s requirements are incompatible with the CC BY 4.0 license, please either i) remove the figure or ii) supply a replacement figure that complies with the CC BY 4.0 license. Please check copyright information on all replacement figures and update the figure caption with source information. If applicable, please specify in the figure caption text when a figure is similar but not identical to the original image and is therefore for illustrative purposes only.

The following resources for replacing copyrighted map figures may be helpful:

USGS National Map Viewer (public domain): http://viewer.nationalmap.gov/viewer/

The Gateway to Astronaut Photography of Earth (public domain): http://eol.jsc.nasa.gov/sseop/clickmap/

Maps at the CIA (public domain): https://www.cia.gov/library/publications/the-world-factbook/index.html and https://www.cia.gov/library/publications/cia-maps-publications/index.html

NASA Earth Observatory (public domain): http://earthobservatory.nasa.gov/

Landsat: http://landsat.visibleearth.nasa.gov/

USGS EROS (Earth Resources Observatory and Science (EROS) Center) (public domain): http://eros.usgs.gov/#

Natural Earth (public domain): http://www.naturalearthdata.com/

8. Please ensure that you refer to Figure 4 in your text as, if accepted, production will need this reference to link the reader to the figure.

9. We notice that your supplementary [tables A1, A2] are included in the manuscript file. Please remove them and upload them with the file type 'Supporting Information'. Please ensure that each Supporting Information file has a legend listed in the manuscript after the references list.

10. If the reviewer comments include a recommendation to cite specific previously published works, please review and evaluate these publications to determine whether they are relevant and should be cited. There is no requirement to cite these works unless the editor has indicated otherwise.

[Note: HTML markup is below. Please do not edit.]

Reviewers' comments:

Reviewer's Responses to Questions

**Comments to the Author**

1. Is the manuscript technically sound, and do the data support the conclusions?

The manuscript must describe a technically sound piece of scientific research with data that supports the conclusions. Experiments must have been conducted rigorously, with appropriate controls, replication, and sample sizes. The conclusions must be drawn appropriately based on the data presented.

Reviewer #1: Yes

Reviewer #2: Yes

**********

2. Has the statistical analysis been performed appropriately and rigorously?

Reviewer #1: Yes

Reviewer #2: No

**********

3. Have the authors made all data underlying the findings in their manuscript fully available?

The PLOS Data policy requires authors to make all data underlying the findings described in their manuscript fully available without restriction, with rare exception (please refer to the Data Availability Statement in the manuscript PDF file). The data should be provided as part of the manuscript or its supporting information, or deposited to a public repository. For example, in addition to summary statistics, the data points behind means, medians and variance measures should be available. If there are restrictions on publicly sharing data—e.g. participant privacy or use of data from a third party—those must be specified.

Reviewer #1: No

Reviewer #2: Yes

**********

4. Is the manuscript presented in an intelligible fashion and written in standard English?

PLOS ONE does not copyedit accepted manuscripts, so the language in submitted articles must be clear, correct, and unambiguous. Any typographical or grammatical errors should be corrected at revision, so please note any specific errors here.

Reviewer #1: Yes

Reviewer #2: Yes

**********

5. Review Comments to the Author

Please use the space provided to explain your answers to the questions above. You may also include additional comments for the author, including concerns about dual publication, research ethics, or publication ethics. (Please upload your review as an attachment if it exceeds 20,000 characters)

Reviewer #1: Agricultural Land Degradation Consequences as a Migration Driver in Developing Countries

Reviewer Comments

This manuscript explores the role of Agricultural Land Degradation (ALD), especially soil salinity, as a driver of rural-urban migration in Egypt. The topic is timely and important, contributing to the growing literature on climate-induced migration in the Global South. The manuscript is based on a robust sample and applies Protection Motivation Theory (PMT) and a multinomial logistic regression model to analyze migration intentions.

However, the paper would benefit from some moderate revisions to improve conceptual clarity, methodological rigor, and academic writing. Below are detailed comments.

Title: Consider making it more specific by including “Egypt” as the country case study.

Abstract: Remove redundancy (e.g., repeating “migration willingness”) and clarify the novel contribution.

Introduction: Better delineate the knowledge gap the paper addresses. Please clarify better how this study extends existing literature beyond identifying the problem. Eg….While existing research has explored the relationship between economic factors and migration, few studies have specifically analyzed how ALD influences migrants' decisions, particularly in terms of migration timing. [Reference these works] Most prior research has focused on general migration drivers, whereas our study sheds light on the more specific…[Reference these works]

Literature Review: Whilst the literature engages with a broad set of sources from multiple geographies, it is too descriptive. More analytical synthesis is needed—compare, contrast, and critique existing works. Include more regionally comparative insights, especially from other North African or Middle Eastern contexts.

5. Theoretical Framework: PMT is a novel and creative theoretical lens for migration, but Better justify why PMT is appropriate in this migration context; originally used in health behavior studies. Consider complementing PMT with Livelihoods Vulnerability Framework or Push-Pull models to enhance explanatory power.

6. Methodology: More detail is needed on how variables were operationalized (e.g., how is "migration intention" coded?). Consider adding a table summarizing variable definitions and expected relationships. Explain potential biases and limitations, e.g., is Itsa District representative of broader Egypt? Include details on ethical clearance and informed consent.

Results and Discussion: Well-structured and includes strong descriptive and inferential statistics.

Writing and Language: The manuscript needs significant language polishing. Some sentences are overly long or grammatically incorrect. Examples: “the capital Cairo (40.2%) and international destinations (29.4%) were the most desired.” → “Among those willing to migrate, Cairo (40.2%) and international destinations (29.4%) were preferred.” Use of commas, conjunctions, and passive voice should be corrected throughout.

Policy and Practical Implications: The discussion on policy implications is promising but too brief and generic. Expand on Targeted adaptation strategies, Links to SDGs, especially SDG 13 (Climate Action) and SDG 10 (Reduced Inequalities), Role of government, NGOs, and international donors in mitigating ALD.

Conclusion: Whilst the conclusion summarizes findings clearly, consider distilling 2–3 key takeaways for policymakers and academics. Include a brief note on future research directions, e.g., longitudinal studies or gender-specific analyses.

Reviewer #2: Overall, title of the paper, claim, has not been well proven. Specifically, more work is required for methodology and analysis. Analysis and theoretical framework are not well connected, accordingly, the conclusions are not linked with theoretical framework. In conclusion, the paper needs "major revision".

**********

6. PLOS authors have the option to publish the peer review history of their article (what does this mean?). If published, this will include your full peer review and any attached files.

If you choose “no”, your identity will remain anonymous but your review may still be made public.

**Do you want your identity to be public for this peer review?**  For information about this choice, including consent withdrawal, please see our Privacy Policy.

Reviewer #1: **Yes:**  Dr Johannes Bhanye

Reviewer #2: **Yes:**  Yogendra B Gurung

**********

[NOTE: If reviewer comments were submitted as an attachment file, they will be attached to this email and accessible via the submission site. Please log into your account, locate the manuscript record, and check for the action link "View Attachments". If this link does not appear, there are no attachment files.]

While revising your submission, please upload your figure files to the Preflight Analysis and Conversion Engine (PACE) digital diagnostic tool, https://pacev2.apexcovantage.com/. PACE helps ensure that figures meet PLOS requirements. To use PACE, you must first register as a user. Registration is free. Then, login and navigate to the UPLOAD tab, where you will find detailed instructions on how to use the tool. If you encounter any issues or have any questions when using PACE, please email PLOS at figures@plos.org. Please note that Supporting Information files do not need this step.

Attachment Submitted filename: Reviewers Comments.pdf

Attachment Submitted filename: PONE-D-25-21257 - Review.docx

# Author comment: Author response to Decision Letter 1

**DOI:** 10.1371/journal.pone.0353721.r002

14 Oct 2025

We attached file. Please check

Attachment Submitted filename: 2Response-to-Reviewer-Comments-Form-PONE-D-25-21257.docx

# Peer review: Decision Letter 1

**DOI:** 10.1371/journal.pone.0353721.r003

**Authors:** Gouranga Dasvarma, Gouranga Dasvarma

28 Apr 2026

PONE-D-25-21257R1

Agricultural Land Degradation Consequences as a Migration Driver in Egypt

PLOS One

Dear Dr. Ali,

Thank you for submitting your manuscript to PLOS ONE. After careful consideration, we feel that it has merit but does not fully meet PLOS ONE’s publication criteria as it currently stands. Therefore, we invite you to submit a revised version of the manuscript that addresses the points raised during the review process.

Please submit your revised manuscript by Jun 12 2026 11:59PM. If you will need more time than this to complete your revisions, please reply to this message or contact the journal office at plosone@plos.org. When you're ready to submit your revision, log on to https://www.editorialmanager.com/pone/ and select the 'Submissions Needing Revision' folder to locate your manuscript file.

Please include the following items when submitting your revised manuscript:

- A letter that responds to each point raised by the academic editor and reviewer(s). You should upload this letter as a separate file labeled 'Response to Reviewers'.
- A marked-up copy of your manuscript that highlights changes made to the original version. You should upload this as a separate file labeled 'Revised Manuscript with Track Changes'.
- An unmarked version of your revised paper without tracked changes. You should upload this as a separate file labeled 'Manuscri

If you would like to make changes to your financial disclosure, please include your updated statement in your cover letter. Guidelines for resubmitting your figure files are available below the reviewer comments at the end of this letter.

If applicable, we recommend that you deposit your laboratory protocols in protocols.io to enhance the reproducibility of your results. Protocols.io assigns your protocol its own identifier (DOI) so that it can be cited independently in the future. For instructions see: https://journals.plos.org/plosone/s/submission-guidelines#loc-laboratory-protocols. Additionally, PLOS ONE offers an option for publishing peer-reviewed Lab Protocol articles, which describe protocols hosted on protocols.io. Read more information on sharing protocols at https://plos.org/protocols?utm_medium=editorial-email&utm_source=authorletters&utm_campaign=protocols.

As the corresponding author, your ORCID iD is verified in the submission system and will appear in the published article. PLOS supports the use of ORCID, and we encourage all coauthors to register for an ORCID iD and use it as well. Please encourage your coauthors to verify their ORCID iD within the submission system before final acceptance, as unverified ORCID iDs will not appear in the published article. *Only*  the individual author can complete the verification step; PLOS staff *cannot*  verify ORCID iDs on behalf of authors.

We look forward to receiving your revised manuscript.

Kind regards,

Gouranga Lal Dasvarma, PhD

Academic Editor

PLOS One

Journal Requirements:

If the reviewer comments include a recommendation to cite specific previously published works, please review and evaluate these publications to determine whether they are relevant and should be cited. There is no requirement to cite these works unless the editor has indicated otherwise.

[Note: HTML markup is below. Please do not edit.]

Reviewers' comments:

Reviewer's Responses to Questions

**Comments to the Author**

1. If the authors have adequately addressed your comments raised in a previous round of review and you feel that this manuscript is now acceptable for publication, you may indicate that here to bypass the “Comments to the Author” section, enter your conflict of interest statement in the “Confidential to Editor” section, and submit your "Accept" recommendation.

Reviewer #3: (No Response)

Reviewer #4: (No Response)

**********

2. Is the manuscript technically sound, and do the data support the conclusions?

The manuscript must describe a technically sound piece of scientific research with data that supports the conclusions. Experiments must have been conducted rigorously, with appropriate controls, replication, and sample sizes. The conclusions must be drawn appropriately based on the data presented.

Reviewer #3: Yes

Reviewer #4: Yes

**********

3. Has the statistical analysis been performed appropriately and rigorously?

Reviewer #3: Yes

Reviewer #4: Yes

**********

4. Have the authors made all data underlying the findings in their manuscript fully available?

The PLOS Data policy requires authors to make all data underlying the findings described in their manuscript fully available without restriction, with rare exception (please refer to the Data Availability Statement in the manuscript PDF file). The data should be provided as part of the manuscript or its supporting information, or deposited to a public repository. For example, in addition to summary statistics, the data points behind means, medians and variance measures should be available. If there are restrictions on publicly sharing data—e.g. participant privacy or use of data from a third party—those must be specified.

Reviewer #3: Yes

Reviewer #4: Yes

**********

5. Is the manuscript presented in an intelligible fashion and written in standard English?

PLOS ONE does not copyedit accepted manuscripts, so the language in submitted articles must be clear, correct, and unambiguous. Any typographical or grammatical errors should be corrected at revision, so please note any specific errors here.

Reviewer #3: Yes

Reviewer #4: Yes

**********

6. Review Comments to the Author

Please use the space provided to explain your answers to the questions above. You may also include additional comments for the author, including concerns about dual publication, research ethics, or publication ethics. (Please upload your review as an attachment if it exceeds 20,000 characters)

Reviewer #3: -Good script, except for

- overloading words, reduce too much writing

- the ALP should be logic 1 in the analysis since it is the main determinant variable

Reviewer #4: The manuscript titled “Agricultural Land Degradation Consequences as a Migration Driver in Egypt” investigates the relationship between agricultural land degradation (ALD), particularly soil salinity, and migration intentions using Protection Motivation Theory (PMT) and a multinomial logistic regression model based on primary survey data (n = 1,782). The topic is timely and policy-relevant, especially in the context of climate change, rural vulnerability, and environmental migration. However, the manuscript in its current form requires substantial revision due to conceptual ambiguity, methodological limitations, insufficient analytical depth, and issues related to writing clarity and structure.

Conceptual and Theoretical Framework

The manuscript introduces Protection Motivation Theory (PMT) as the central theoretical framework and attempts to combine it with push–pull theory and livelihood approaches. While this is potentially innovative, the integration remains superficial. The key constructs of PMT—such as threat appraisal and coping appraisal—are not clearly operationalized into measurable variables, nor are they empirically tested within the model. As a result, PMT functions more as a descriptive narrative rather than an analytical framework. Additionally, the simultaneous use of multiple theoretical perspectives without clear differentiation leads to conceptual overlap and weakens the overall coherence of the study. A more focused and explicitly operationalized theoretical framework is necessary.

Methodology and Data

The study employs a multinomial logistic regression model, which is appropriate given the categorical nature of migration intention. The sampling strategy is described as multi-stage cluster sampling within Fayoum Governorate. However, the generalizability of the findings is overstated, as the analysis is based on a single district (Itsa), which may not represent broader national dynamics . Furthermore, key variables such as agricultural land degradation are measured through self-reported perceptions rather than objective environmental indicators, raising concerns about measurement bias.

The model specification lacks sufficient rigor. There is no discussion of important assumptions such as independence of irrelevant alternatives (IIA), nor are there diagnostics for multicollinearity or robustness checks. Although the model fit statistics are reported, the explanatory power remains moderate (Nagelkerke R² ≈ 0.278) , and this limitation is not adequately discussed.

Results and Interpretation

The results section presents statistically significant findings regarding demographic and socioeconomic determinants of migration intention. However, the interpretation often overreaches by implying causal relationships, despite the cross-sectional design of the study. The linkage between empirical findings and PMT remains weak, as coefficients are not clearly mapped onto theoretical constructs. Additionally, the analysis lacks depth, as it does not explore interaction effects or subgroup heterogeneity (e.g., gender, land ownership, or income levels), which could provide more nuanced insights into migration dynamics.

Writing, Structure, and Presentation

The manuscript suffers from issues related to language clarity, redundancy, and organization. Several sections, particularly the literature review are overly descriptive and repetitive, with limited critical synthesis. Transitions between sections (e.g., theory to methods, results to discussion) are not always smooth, affecting the overall readability. There are also frequent grammatical errors and stylistic inconsistencies that require professional language editing. Figures, particularly those illustrating the conceptual framework, are informative but visually dense and could be simplified for better clarity.

Ethical and Reporting Issues

The ethics statement provided in the manuscript is insufficient. The statement that the data were analyzed anonymously does not meet standard reporting requirements, as it lacks information on institutional ethical approval and consent procedures . This needs to be addressed to ensure compliance with journal guidelines.

Conclusion and Recommendation

In conclusion, the manuscript addresses an important and policy-relevant issue and is supported by a substantial primary dataset. However, significant revisions are required to strengthen the theoretical grounding, improve methodological transparency, and enhance analytical rigor. The authors should focus on properly operationalizing PMT, refining the empirical strategy, incorporating robustness checks, and improving the clarity and coherence of the writing.

**********

7. PLOS authors have the option to publish the peer review history of their article (what does this mean?). If published, this will include your full peer review and any attached files.

If you choose “no”, your identity will remain anonymous but your review may still be made public.

**Do you want your identity to be public for this peer review?**  For information about this choice, including consent withdrawal, please see our Privacy Policy.

Reviewer #3: **Yes:**  Florence Akiakum Agweibab

Reviewer #4: No

**********

[NOTE: If reviewer comments were submitted as an attachment file, they will be attached to this email and accessible via the submission site. Please log into your account, locate the manuscript record, and check for the action link "View Attachments". If this link does not appear, there are no attachment files.]

To ensure your figures meet our technical requirements, please review our figure guidelines: https://journals.plos.org/plosone/s/figures

You may also use PLOS’s free figure tool, NAAS, to help you prepare publication quality figures: https://journals.plos.org/plosone/s/figures#loc-tools-for-figure-preparation.

NAAS will assess whether your figures meet our technical requirements by comparing each figure against our figure specifications.

Attachment Submitted filename: PONE-D-25-21257_R1_Florence.pdf

# Author comment: Author response to Decision Letter 2

**DOI:** 10.1371/journal.pone.0353721.r004

12 Jun 2026

The attached file contains the detailed responses to each reviewer's comments.

Attachment Submitted filename: R2Response-to-Reviewer-Comments-Form-PONE-D-25-21257R1.docx

# Editor report: Decision Letter 2

**DOI:** 10.1371/journal.pone.0353721.r005

**Authors:** Gouranga Dasvarma, Gouranga Dasvarma, Gouranga Dasvarma

28 Jun 2026

Agricultural Land Degradation Consequences as a Migration Driver in Egypt

PONE-D-25-21257R2

Dear Dr. Abdelrahman Ali,

We’re pleased to inform you that your manuscript has been judged scientifically suitable for publication and will be formally accepted for publication once it meets all outstanding technical requirements.

Within one week, you’ll receive an e-mail detailing the required amendments. When these have been addressed, you’ll receive a formal acceptance letter and your manuscript will be scheduled for publication.

An invoice will be generated when your article is formally accepted. Please note, if your institution has a publishing partnership with PLOS and your article meets the relevant criteria, all or part of your publication costs will be covered. Please make sure your user information is up-to-date by logging into Editorial Manager at Editorial Manager® and clicking the ‘Update My Information' link at the top of the page. For questions related to billing, please contact billing support.

If your institution or institutions have a press office, please notify them about your upcoming paper to help maximize its impact. If they’ll be preparing press materials, please inform our press team as soon as possible -- no later than 48 hours after receiving the formal acceptance. Your manuscript will remain under strict press embargo until 2 pm Eastern Time on the date of publication. For more information, please contact onepress@plos.org.

Kind regards,

Gouranga Lal Dasvarma, PhD

Academic Editor

PLOS One

Additional Editor Comments (optional):

Reviewers' comments:

# Editor report: Acceptance letter

**DOI:** 10.1371/journal.pone.0353721.r006

**Authors:** Gouranga Dasvarma, Gouranga Dasvarma, Gouranga Dasvarma

PONE-D-25-21257R2

PLOS One

Dear Dr. Ali,

I'm pleased to inform you that your manuscript has been deemed suitable for publication in PLOS One. Congratulations! Your manuscript is now being handed over to our production team.

At this stage, our production department will prepare your paper for publication. This includes ensuring the following:

* All references, tables, and figures are properly cited

* All relevant supporting information is included in the manuscript submission,

* There are no issues that prevent the paper from being properly typeset

You will receive further instructions from the production team, including instructions on how to review your proof when it is ready. Please keep in mind that we are working through a large volume of accepted articles, so please give us a few days to review your paper and let you know the next and final steps.

Lastly, if your institution or institutions have a press office, please let them know about your upcoming paper now to help maximize its impact. If they'll be preparing press materials, please inform our press team within the next 48 hours. Your manuscript will remain under strict press embargo until 2 pm Eastern Time on the date of publication. For more information, please contact onepress@plos.org.

You will receive an invoice from PLOS for your publication fee after your manuscript has reached the completed accept phase. If you receive an email requesting payment before acceptance or for any other service, this may be a phishing scheme. Learn how to identify phishing emails and protect your accounts at https://explore.plos.org/phishing.

If we can help with anything else, please email us at customercare@plos.org.

Thank you for submitting your work to PLOS ONE and supporting open access.

Kind regards,

PLOS ONE Editorial Office Staff

on behalf of

Dr. Gouranga Lal Dasvarma

Academic Editor

PLOS One
